# Superelectrophilic activation of 5-hydroxymethylfurfural and 2,5-diformylfuran: organic synthesis based on biomass-derived products

**DOI:** 10.3762/bjoc.12.202

**Published:** 2016-10-05

**Authors:** Dmitry S Ryabukhin, Dmitry N Zakusilo, Mikhail O Kompanets, Anton A.Tarakanov, Irina A Boyarskaya, Tatiana O Artamonova, Mikhail A Khohodorkovskiy, Iosyp O Opeida, Aleksander V Vasilyev

**Affiliations:** 1 Department of Chemistry, Saint Petersburg State Forest Technical University, Institutsky per., 5, Saint Petersburg, 194021, Russia; 2 Institute of Chemistry, Saint Petersburg State University, Saint Petersburg State University, Universitetskaya nab., 7/9, Saint Petersburg, 199034, Russia; 3 The All-Russia Scientific Research Institute of Fats, ul. Chernyakhovskogo, 10, Saint Petersburg, 191119, Russia; 4 L.M. Litvinenko Institute of Physico-Organic and Coal Chemistry of NASU, Kharkivs’ke Hgw, 50, Kiyv, 02160, Ukraine; 5 Institute of Nanobiotechnologies, Peter the Great St. Petersburg Polytechnic University, Polytechnicheskaya ul., 29, Saint Petersburg, 195251, Russia; 6 Department of Physical Chemistry of Combustible Minerals, L.M. Litvinenko Institute of Physical Organic and Coal Chemistry of NASU, Naukova St., 3a, Lviv, 79053, Ukraine

**Keywords:** 2,5-diformylfuran, Friedel–Crafts reaction, 5-hydroxymethylfurfural, superacids, zeolites

## Abstract

The reaction of 5-hydroxymethylfurfural (5-HMF) with arenes in superacidic trifluoromethanesulfonic acid (triflic acid, TfOH) as the solvent at room temperature for 1–24 h gives rise to 5-arylmethylfurfurals (yields of 17–91%) and 2-arylmethyl-5-(diarylmethyl)furans (yields of 10–37%). The formation of these two types of reaction products depends on the nucleophilicity of the arene. The same reactions under the action of acidic zeolites H-USY in high pressure tubes at 130 °C for 1 h result in the formation of only 5-arylmethylfurfurals (yields of 45–79%). 2,5-Diformylfuran (2,5-DFF) in the reaction with arenes under the action of AlBr_3_ at room temperature for 1 h leads to 5-(diarylmethyl)furfurals (yields of 51–90%). The reactive protonated species of 5-HMF and 2,5-DFF were characterized by NMR spectroscopy in TfOH and studied by DFT calculations. These reactions show possibilities of organic synthesis based on biomass-derived 5-HMF and 2,5-DFF.

## Introduction

Nowadays great attention is paid to the use of renewable resources for obtaining fine chemicals and fuels (see numerous reviews [1–16]). The biorefinery of renewable lignin-carbohydrate materials affords various low-molecular weight organic molecules, such as alcohols, carboxylic acids, (hetero)aromatic ketones and aldehydes, phenols, etc. These biomass-derived platform chemicals are considered as an alternative and displacement to petroleum chemistry [17–18]. Among all these compounds, the preparation and reaction of 5-hydroxymethylfurfural (5-HMF) attracts special attention (see fundamental review from 2013 [19] and recent papers [20–25]). The high functionality and reactivity of 5-HMF, due to the presence of oxymethyl and aldehyde substituents, along with the furan moiety, allows many transformations and therefore the production of new useful organic substances [26–30].

**Figure 1:**
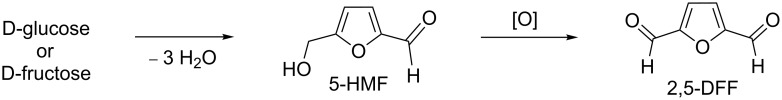
Formation of 5-HMF from D-glucose or D-fructose followed by oxidation to 2,5-DFF.

Based on our recent study on the synthesis of 5-HMF and its oxidation to 2,5-diformylfuran (2,5-DFF) [31] (Figure 1), this work is focused on developing methods of organic synthesis on the basis of electrophilic activation of these biomass-derived products.

Superelectrophilic activation is the generation of highly reactive di-, tri- (or even higher) cationic species by protonation and protosolvation of organic molecules with low nucleophilic Brønsted superacids, such as CF_3_SO_3_H (TfOH) or FSO_3_H [32]. The same activation may be achieved with strong Lewis acids (AlX_3_, X = Cl, Br) by their coordination with basic centers of organic compounds, or with acidic zeolites, possessing both Brønsted and Lewis acidity [33].

The main goal of this work was a study of reactions of 5-HMF and 2,5-DFF with arenes under electrophilic activation with Brønsted and Lewis superacids. Previously superelectrophilic activation of aldehyde groups was achieved for heteroaromatic aldehydes [34–37], substituted benzaldehydes and *o*-phthalic dicarboxaldehyde [38]. Based on these findings, one would expect the activation of an aldehyde group of 5-HMF and 2,5-DFF, and its participation in the hydroxyalkylation of arenes. However, furan carboxaldehydes in such reactions were studied in this work for the first time.

It should be noted, that reactions of arenes with hydroxymethyl and aldehyde groups of 5-HMF and 2,5-DFF may lead to various arylmethyl- and diarylmethyl-substituted furans, which are otherwise hardly available molecules [39–46] and used for the synthesis of bioactive compounds [47]. Thus, the superelectrophilic activation of 5-HMF and 2,5-DFF could be of great value for organic synthesis.

## Results and Discussion

**Scheme 1:**
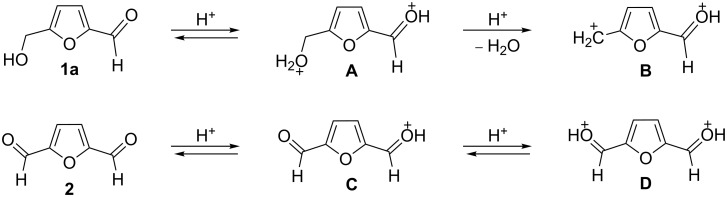
Protonation of 5-HMF (**1a**) and 2,5-DFF (**2**) leading to cationic species **A**, **B**, **C**, **D**.

The protonation of the carbonyl oxygen and hydroxy group of 5-HMF (**1a**) in strong acids gives rise to cationic species **A**, the dehydration of the latter may result in the formation of heteroaromatic benzyl-type dication **B** (Scheme 1). Protonation of the aldehyde groups in 2,5-DFF (**2**) leads to cation **C** and dication **D** (Scheme 1). All species **A**, **B**, **C**, and **D** may play a role as reactive intermediates derived from **1a** and **2** in superacids. To estimate the electrophilic properties of cations **A**, **B**, **C**, and **D** we performed quantum chemical calculations by the DFT method (Table 1). HOMO and LUMO energies, global electrophilicity index ω [48–49], charge distribution, and contribution of atomic orbitals into the LUMO were calculated. The dications **B** and **D** having high ω values of 9.6 and 8 eV, respectively, should be very reactive electrophiles. The carbon atom of the protonated aldehyde group in species **A**, **B**, **C**, and **D** bears a large positive charge and shows a great contribution in the LUMO. This indicates that this carbon atom may be an electrophilic reactive center from both charge and orbital point of view. Apart from that, the increase of positive charge on heteroaromatic carbons C1, C4 and the decrease of negative charge on atoms C2, C3, and O_furan_ upon protonation of furans **1a** and **2** reveal a significant positive charge delocalization into the furan ring in species **A**, **B**, **C**, and **D**. However, from these calculations no unambiguous answer could be obtained that reveals what cations in pairs **A** or **B**, and **C** or **D** may take part in electrophilic reactions.

**Table 1:** Selected electronic characteristics (DFT calculations) of starting furans **1a**, **2** and cationic species **A**, **B**, **C**, and **D**.

Species	*E*_HOMO_, eV	*E*_LUMO_, eV	ω,^a^ eV	q(CH_2_),^b^ e	q(C=O),**^b^** e	k(CH_2_)_LUMO_,**^с^** %	k(C=O)_LUMO_,^с^ %

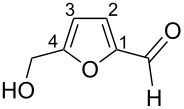 **1a**	−6.82	−2.20	2.2	−0.066	0.379	0.6	27.8
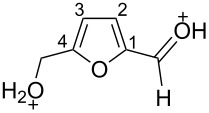 **A**	−8.93	−4.59	5.3	−0.060	0.428 (COH^+^)	6.9	26.3 (COH^+^)
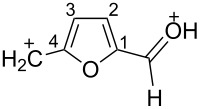 **B**	−10.43	−6.65	9.6	0.054	0.528 (COH^+^)	26.1	24.0 (COH^+^)
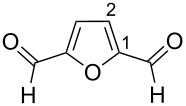 **2**	−7.43	−3.06	3.1	–	0.391	–	15.4
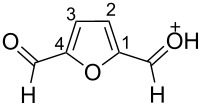 **C**	−8.57	−4.60	5.5	–	0.400 0.417 (COH^+^)	−	5.7 31.2 (COH^+^)
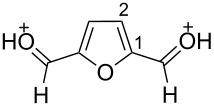 **D**	−9.78	−5.91	8.0	–	0.488 (COH^+^)	–	22.9 (COH^+^)

Species	q(C^1^),^b^ e	q(C^2^),^b^ e	q(C^3^),^b^ e	q(C^4^),^b^ e	q(O_furan_),^b^ e		

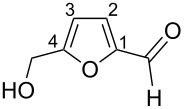 **1a**	0.152	−0.192	−0.302	0.352	−0.464		
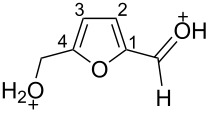 **A**	0.158	−0.097	−0.220	0.371	−0.406		
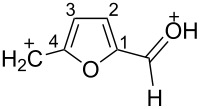 **B**	0.313	−0.162	−0.010	0.209	−0.374		
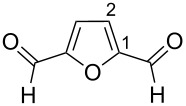 **2**	0.210	−0.209	−0.209	0.210	−0.568		
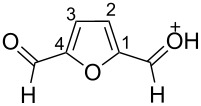 **C**	0.161	−0.106	−0.217	0.330	−0.404		
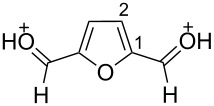 **D**	0.228	−0.119	−0.119	0.228	−0.376		

^a^Global electrophilicity index ω = (*E*_HOMO_ + *E*_LUMO_)^2^/8(*E*_LUMO_ − *E*_HOMO_). ^b^Natural charges. ^c^Contribution of atomic orbitals into the molecular orbital.

To investigate this issue in more detail we studied the protonation of furans **1a** and **2** in the superacid TfOH by NMR spectroscopy. Upon dissolving **1a** and **2** in TfOH in the NMR tube the formation of deep-red solutions was observed. ^1^H and ^13^C NMR data of the generated cationic species are collected in Table 2 (see spectral figures in Supporting Information File 1). The spectral data clearly prove that protonation of **1a** in TfOH gives rise to dication **A** (Scheme 1). Thus, in the ^13^C NMR spectrum the signal at δ 67.5 ppm belongs to the protonated hydroxymethyl group CH_2_O^+^H_2_. This means that dehydration of this group does not occur in TfOH and cation **B** is not formed. For comparison, chemical shifts of carbocationic centers in various benzyl cations (R_2_)ArC^+^ lie in a much more down-field range of ~182–270 ppm [50–57]. Comparison of ^1^H and ^13^C NMR spectra shows that signals of protons and carbons in the furan ring of **A** are substantially down-field shifted in comparison with the corresponding signals of its neutral precursor **1a**. This reveals a significant positive charge delocalization in the furan ring in **A** that is in good agreement with the DFT calculations (vide supra).

**Table 2:** ^1^H and ^13^C NMR data of furans **1a, 2** in CDCl_3_ and species **A**, **D** in TfOH at room temperature.

Species	Solvent	NMR data	

		^1^H	^13^C

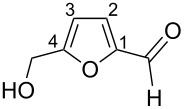 **1a**	CDCl_3_	2.84 (s, 1H, OH), 4.71 (s, 2H, CH_2_), 6.51 (d,(*J* = 3.5 Hz, 1H, H2), 7.21 (d, *J* = 3.5 Hz, 1H, H3), 9.57 (s, 1H, CHO)	57.6 (CH_2_), 109.9 (C2), 122.8 (C3), 152.4 (C4), 160.7 (C1), 177.7 (CHO)
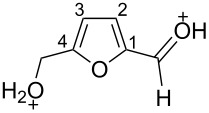 **A**	TfOH	5.85 (s, 2H, CH_2_), 7.44 (d, *J* = 4.2 Hz, 1H, H2), 8.79 (d, *J* = 4.2 Hz, 1H, H3), 9.04 (s, C=OH^+^)	67.5 (CH_2_), 121.0 (C2), 148.5 (C3), 152.8 (C4), 171.9 (C1), 175.9 (C=OH^+^)
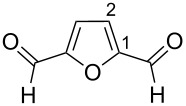 **2**	CDCl_3_	7.33 (s, 2H, H2), 9.86 (s, CHO)	119.1 (C2), 154.2 (C1), 179.2 (CHO)
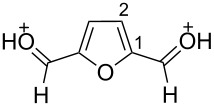 **D**	TfOH	8.48 (s, 2H), 9.84 (s, C=OH^+^)	136.8 (C2), 156.0 (C1), 186.6 (C=OH^+^)

According to the NMR data (Table 2 and Supporting Information File 1) protonation of **2** in TfOH leads to the *O*,*O*-diprotonated species **D**. Analogous to dication **A**, a down-field shift of signals of protons and carbons of the furan ring in ^1^H and ^13^C NMR spectra of **D** compared to the signals for **2** was observed, pointing out a charge delocalization in the furan moiety. Also, the down-field shift of the ^13^C signal of the protonated aldehyde group in species **D** indicates that this carbon should be a reactive electrophilic center. Contrary to that, the signal of the carbon of the protonated aldehyde group in **A** is even slightly up-field shifted compared to the same signal in **1a**. This indicates weak electrophilic properties of the protonated aldehyde group in **A**. Signals of protons bonded to aldehyde and oxymethyl groups in species **A** and **D** were not registered in ^1^H NMR due to the fast proton exchange with the superacidic medium at room temperature.

Thus, the NMR data revealed that protonation of furans **1a** and **2** in superacid TfOH resulted in the formation of dications **A** and **D**, respectively, although DFT calculations (Table 1) did not manifest that clearly.

Next, 5-HMF (**1a**), 5-(chloromethyl)furfural (**1b**) and 5-(bromomethyl)furfural (**1c**), both of which are also promising biomass-derived products [58–59], were reacted with benzene under the action of various acids (Table 3). In all cases 5-(phenylmethyl)furfural (**3a**) as Friedel–Crafts reaction product was obtained. Thus, only the hydroxymethyl or halogenomethyl group in furans **1a–c** were involved in the reactions. The aldehyde group remained intact despite the DFT calculations predicted a high electrophilicity for the carbon of the protonated aldehyde group (Table 1). The best results (the highest yield of **3a**) were achieved with TfOH at room temperature for 1 h (Table 3, entries 1, 7, and 9). Other acids were less efficient, leading to **3a** in lower yields or harsher conditions (130 °C) were required for acidic zeolites H-USY, CBV-720 and CBV-500 (Table 3, entries 3, 4, 8). Since the NMR data showed the generation of dication **A** from **1a** (Table 2), this reaction, most probably, proceeds through an S_N_2 pathway, where “pure” heteroaromatic cation **B** is not formed. At least the reaction may go through late transition state, in which the C–O bond in the CH_2_O^+^H_2_ group is rather elongated, resulting in a larger positive charge on this carbon than it has been predicted by calculations (see Table 1). It should be noted, that the yields of reaction products in Tables 3–5 are isolated yields after column chromatographic separation. The remaining materials are some oligomeric compounds.

**Table 3:** Reactions of furans **1a–c** with benzene under the action of various acids.

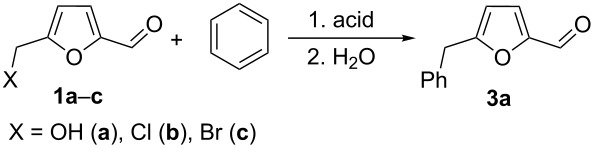					

Entry	Reaction conditions				Yield of **3a**,^a^ %

	Furan	Acid	*T*, °C	*t*, h	

1	**1a**	TfOH (20 equiv)	rt	1	91
2	**1a**	TfOH (20 equiv)	rt	1	–^b^
3	**1a**	zeolite CBV-500^c^	130	3	47
4	**1a**	zeolite CBV-720^c^	130	1	45
5	**1a**	H_2_SO_4_ (50 equiv)	rt	1	19
6	**1a**	AlCl_3_ (5 equiv)	rt	1	oligomers
7	**1b**	TfOH (20 equiv)	rt	1	75
8	**1b**	zeolite CBV-500	130	3	25
9	**1c**	TfOH (20 equiv)	rt	1	68

^a^Isolated yields. ^b^Reaction was carried out without benzene, and starting **1a** was quantitatively recovered. ^c^The ratio of **1a–c**/zeolite Brønsted acidic sites was around 2:1.

Using these conditions (TfOH, rt, 1 h), we carried out reactions of 5-HMF (**1a**) with various arenes in TfOH. Additionally we investigated the reactions under the action of zeolite CBV-720, since zeolites are considered as “green” catalysts in organic synthesis [60–66] and the data are collected in Table 4. Similarly to the reaction with benzene (Table 3), **1a** with other arenes yielded 5-arylmethylfurfurals **3b–o** in TfOH or with zeolite CBV-720. However, the use of activated arenes, such as toluene, xylenes and pseudocumene, afforded additional Friedel–Crafts products, namely furans **4a–e** (Table 4, entries 1, 2, 4–8, 11–13, and 15). These compounds were formed by hydroxyalkylation due to the interaction of species **A** with arenes (see related reactions [34–38]). The polymethylated arenes possess a sufficient π-nucleophilicity for the reaction with the protonated aldehyde group of the intermediates generated from **1a** in TfOH. Whereas less nucleophilic arenes, such as benzene (Table 3) and 1,2-dichlorobenzene (Table 4, entries 18 and 19), did not give rise to the corresponding furans **4**. Also, the reactions with anisole and veratrole led only to furans **3l**, **m** and **3n**, respectively, and no formation of compounds **4** was observed (Table 4, entries 20–23). Under the superacidic conditions the substrates anisole and veratrole may be protonated at the oxygen atoms [33], thus leading to a decreased π-nucleophilicity of these arenes. It should be noted that in the case of zeolites we explored CS_2_ as low coordinating solvent to avoid blocking zeolite acidic sites by π-donating arenes.

**Table 4:** Reactions of 5-HMF (**1a**) with arenes under the action of TfOH or acidic zeolite CBV-720.

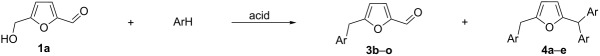									

Entry	ArH	Reaction conditions					Reaction products^a^		Total yield^a^, %
			
		ArH (equiv)	acid (equiv)	*T*, °C	*t*, h		**3**	**4**	

1	toluene	1.2	TfOH (20)	rt	1		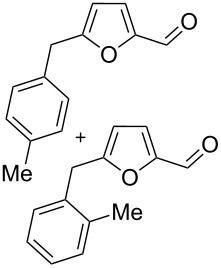 **3b** (23%), **3c** (23%)	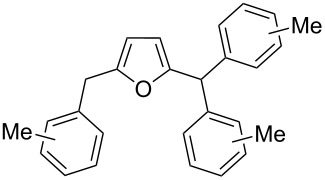 isomers-**4a** (10%)	56
2	toluene	4	TfOH (20)	rt	3		**3b** (44%), **3c** (44%)	isomers-**4a** (10%)	98
3	toluene	125	CBV-720^b^	130	1		**3b** (24%), **3c** (24%)	–	48
4	*o*-xylene	4	TfOH (20)	rt	1		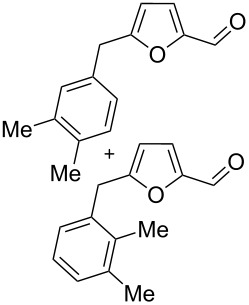 **3d** (32%), **3e** (22%)	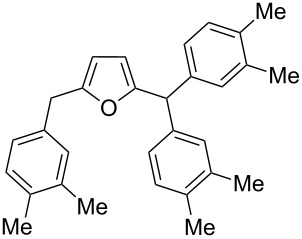 **4b** (23%)	77
5	*o*-xylene	4	TfOH (20)	rt	24		**3d** (37%), **3e** (22%)	**4b** (37%)	96
6	*m*-xylene	1.2	TfOH (20)	rt	1		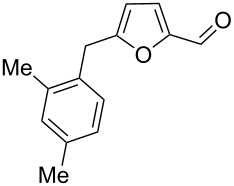 **3f** (28%)	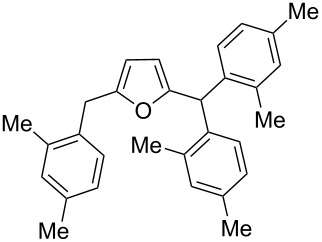 **4c** (6%)	34
7	*m*-xylene	4	TfOH (20)	rt	3		**3f** (75%)	**4c** (20%)	95
8	*m*-xylene	4	TfOH (20)	rt	24		**3f** (12%)	**4c** (32%)	44
9	*m*-xylene	4	TfOH (20)	rt	72		oligomers		–
10	*m*-xylene	2.5	CBV-720^b^, CS_2_	130	1		**3f** (79%)	–	79
11	*p*-xylene	1.2	TfOH (20)	rt	1		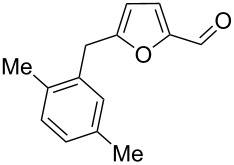 **3g** (53%)	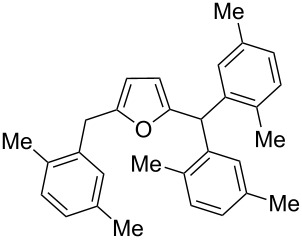 **4d** (18%)	71
12	*p*-xylene	4	TfOH (20)	rt	3		**3g** (91%)	**4d** (5%)	96
13	*p*-xylene	4	TfOH (20)	rt	24		**3g** (21%)	**4d** (17%)	38
14	*p*-xylene	4	CBV-720^b^, CS_2_	130	1		**3g** (78%)	–	78
15	pseudo- cumene	4	TfOH (20)	rt	1		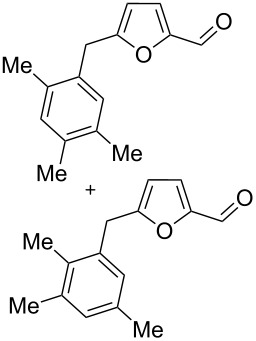 **3h** (28%), **3i** (12%)	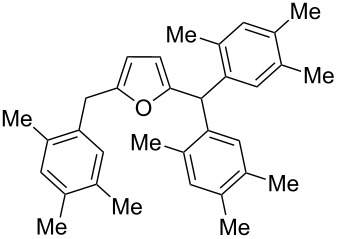 **4e** (32%)	72
16	pseudo- cumene	10	TfOH (20)	rt	24		oligomers		–
17	pseudo- cumene	4	CBV-720^b^, CS_2_	130	1		**3h** (53%), **3i** (23%)	–	76
18	1,2-dichloro-benzene	4	TfOH (20)	rt	1		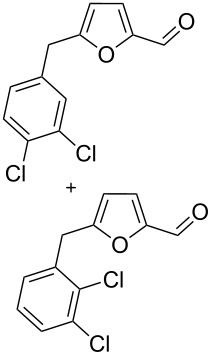 **3j** (36%), **3k** (17%)	–	53
19	1,2-dichloro-benzene	4	CBV-720^a^, CS_2_	130	1		**3j** (7%), **3k** (4%)	–	11
20	anisole	4	TfOH (20)	rt	1		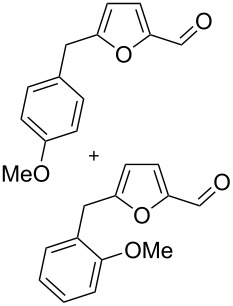 **3l** (42%), **3m** (9%)	–	51
21	anisole	3	CBV-720^b^, CS_2_	130	1		**3l** (34%), **3m** (18%)	–	52
22	veratrole	4	TfOH (20)	rt	2		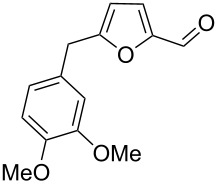 **3n** (62%)	–	62
23	veratrole	4	CBV-720^b^, CS_2_	130	1		**3n** (21%)	–	21
24	mesitylene	5	TfOH (20)	rt	2		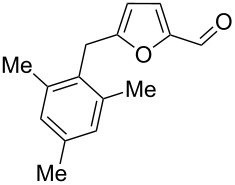 **3o** (17%)	–	17

^a^Isolated yields. ^b^The ratio of **1a**/zeolite Brønsted acidic sites was around 2:1.

Individually isolated compounds **3f** and **3g** being dissolved in TfOH at room temperature for 24 h gave rise to mixtures containing furans **4c** and **4d** along with starting **3f** and **3g**, respectively. This may prove that compounds **4** are the secondary reaction products. It is interesting to note that compounds **4** were not observed in reactions with zeolite (Table 4, entries 3, 10, 14 and 17), most likely, due to lower acidity of the zeolite compared to TfOH and spatial restrictions in zeolite cages, diminishing the contact between protonated aldehyde groups and the substrate (arene) molecules.

We also varied reaction conditions (time, amount of arene) in TfOH to check yields of compounds **3** and **4**. In general, increasing the reaction time up to 3–24 h led to an increase in the yield of furans **4** and a decreased yield of compounds **3** (compare pairs of entries in Table 4: 4 and 5, 7 and 8, 12 and 13). But during long reaction times (24–72 h) compounds **3** and **4** were completely cleaved in TfOH (Table 4, entries 9 and 16), showing complex behavior of these furans under superacidic conditions.

Concerning the reaction mechanism, according to NMR data (see Table 2) the reactive species, derived from **1a** in TfOH, is cation **A**. In zeolite cages the activation of the CH_2_OH group of **1a** takes place due to the coordination of the basic centers of this molecule with Brønsted and Lewis acidic sites of zeolite.

**Figure 2:**
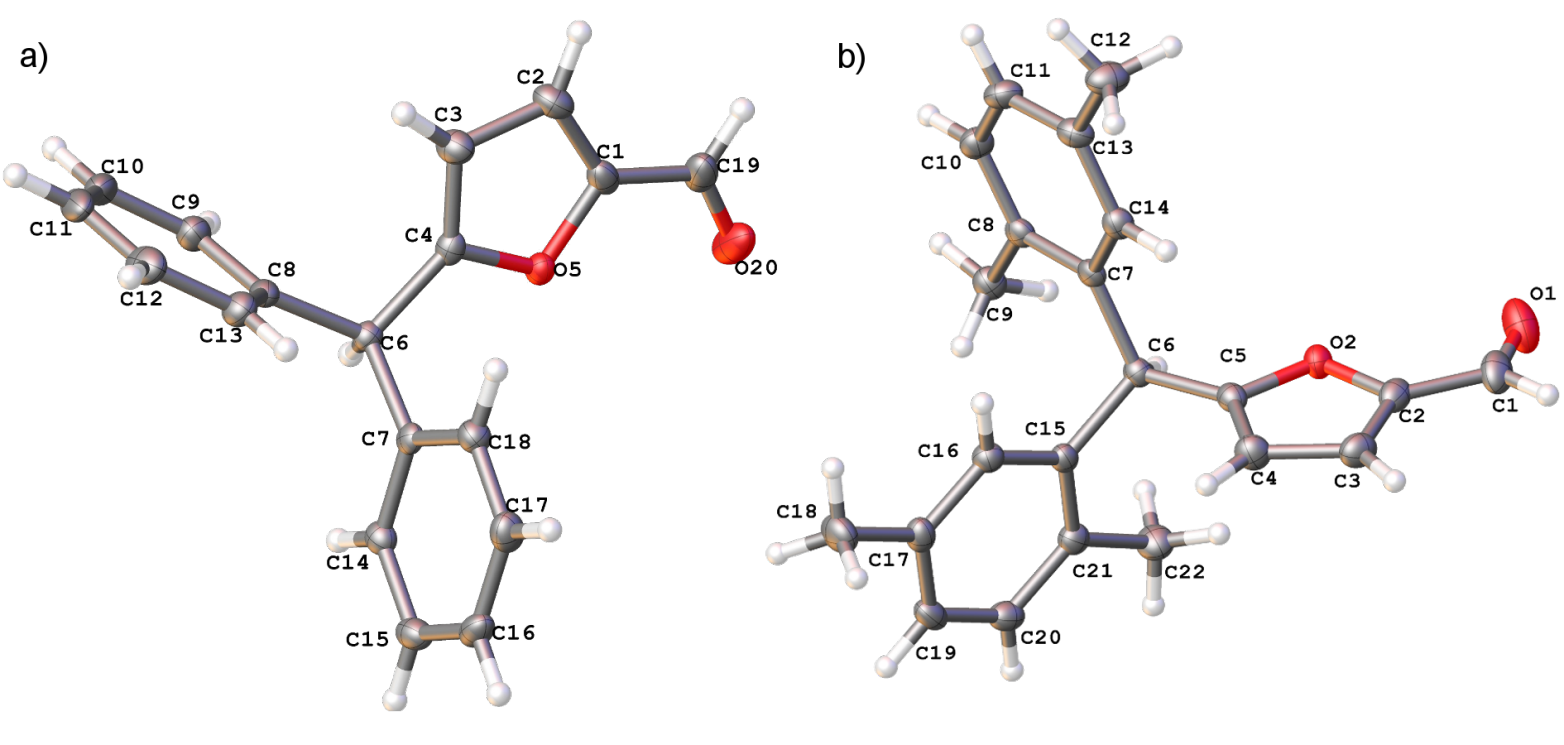
X-ray crystal structure of compounds **5a** (a), and **5c** (b) (ORTEP diagrams, ellipsoid contour of probability levels is 50%, CCDC reference numbers **5a**: 1483523, **5c**: 1483524).

2,5-DFF (**2**) reacted with arenes under electrophilic activation with formation of 5-diarylmethylfurfurals **5a–g** (Table 5, Figure 2). Contrary to compound **1a**, which was activated with the Brønsted superacid TfOH to achieve Friedel–Crafts products **3** and **4** (Table 3 and Table 4), compound **2** gave better results with strong Lewis acids AlX_3_ (X = Cl, Br) (Table 5, entries 4, 5, 9, 11, 13, and 15). The coordination of aluminum halides with the aldehyde group results in the activation of one of the aldehyde groups, catalyzing the reaction subsequently with two arene molecules resulting in the 1,1-diarylated product. The second aldehyde group does not take part in the reaction (compare with data from ref. [38]). This is due to both, an insufficient electrophilic activation by these particular acids and the low nucleophilicity of benzene. Also the reaction of **2** with benzene was promoted by zeolite CBV-500, which is more acidic than CBV-720 (see Supporting Information File 1), and the latter was not be able to give rise to **5a** (Table 5, entries 6–8).

**Table 5:** Reactions of 2,5-DFF **2** with arenes under the action of various acids.

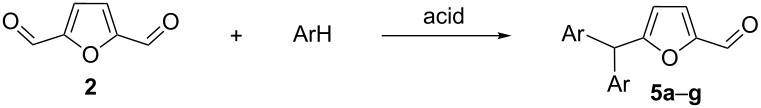							

Entry	ArH	Reaction conditions				Reaction products, **5**	
		
		acid (equiv)	*T*, °C	*t*, h		substituents R in Ar	yield^a^ (%)

1	benzene	TfOH (20)	rt	2		H	**5a** (90%)
2	benzene	H_2_SO_4_ (50)	rt	2		H	**5a** (74%)
3	benzene	FSO_3_H (20), SO_2_	−45	2		H	**5a** (84%)
4	benzene	AlCl_3_ (5)	rt	1		H	**5a** (92%)
5	benzene	AlBr_3_ (5)	rt	1		H	**5a** (98%)
6	benzene	CBV-500^b^	130	1		H	**5a** (13%)^c^
7	benzene	CBV-500^b^	130	10		H	**5a** (46%)
8	benzene	CBV-720^b^	130	10		H	–^d^
9	*o*-xylene	AlBr_3_ (5)	rt	1		3,4-Me_2_	**5b** (72%)
10	*o*-xylene	TfOH (20)	rt	1		oligomers	
11	*m*-xylene	AlBr_3_ (5)	rt	1		3,5-Me_2_	**5c** (36%)
						2,4-Me_2_	**5d** (15%)
12	*m*-xylene	TfOH (20)	rt	1		oligomers	
13	*p*-xylene	AlBr_3_ (5)	rt	1		2,5-Me_2_	**5e** (78%)
14	*p*-xylene	TfOH (20)	rt	1		2,5-Me_2_	**5e** (87%)
15	1,2-dichlorobenzene	TfOH (20)	rt	1		3,4-Cl_2_	**5f** (82%)
						2,3-Cl_2_	**5g** (16%)
16	1,2-dichlorobenzene	AlBr_3_ (5)	rt	1		–^d^	

^a^Isolated yields. ^b^The ratio of **2**/zeolite Brønsted acidic sites was around 2:1. ^c^Incomplete conversion; 55% of starting **2** was recovered. ^d^No reaction and quantitative recovery of starting **2**.

Electron-donating xylenes in the reaction with **2** showed a more complex behavior. The different isomeric xylenes led to compounds **5b–e** in the presence of AlBr_3_ (Table 5, entries 9, 11, and 13). On the other hand, in the Brønsted superacid TfOH *o*- and *m*-xylenes gave rise to complex oligomeric mixtures (Table 5, entries 10 and 12) having masses up to 1400–1500 Da according to MALDI–MS (see Supporting Information File 1). These oligomers may have formed due to the participation of both aldehyde groups of species **D** (see Scheme 1 and Table 2) in the reaction with these electron-rich arenes. Under the action of AlBr_3_ 2,5-DFF did not react with 1,2-dichlorobenzene, due to a too low nucleophilicity of the latter. However, this reaction took place in TfOH (Table 5, entries 15 and 16).

## Conclusion

We have developed a simple and effective synthesis of various arylmethyl and diarylmethyl-substituted furans by reactions of 5-HMF and 2,5-DFF with arenes under electrophilic activation by Brønsted/Lewis (super)acids or acidic zeolites H-USY.

In these reactions 5-HMF in TfOH gives rise to 5-(arylmethyl)furfurals and 2-(arylmethyl)-5-(diarylmethyl)furans. The latter compounds are formed in reactions with donating arenes. Reactions of 5-HMF with arenes under the action of acidic zeolites H-USY result in the selective formation of only 5-(arylmethyl)furfurals. 2,5-DFF in reactions with arenes under the action of AlBr_3_ leads solely to 5-(diarylmethyl)furfurals.

The electrophilic intermediates derived from the protonation of 5-HMF and 2,5-DFF were investigated by means of DFT calculations and NMR in the superacid TfOH. These reactions are a contribution to organic syntheses based on biomass-derived products 5-HMF and 2,5-DFF.

## Supporting Information

File 1 Experimental procedures, characterization of compounds, ^1^H, ^13^C, ^19^F NMR spectra, and data on DFT calculations.
