# Supplementary material for: Superelectrophilic activation of 5-hydroxymethylfurfural and 2,5-diformylfuran: organic synthesis based on biomass-derived products
Source: Beilstein J Org Chem. 2016 Oct 5;12:2125–35. doi: 10.3762/bjoc.12.202 (PMC5082471; doi:10.3762/bjoc.12.202)
Supplement: File 1 — Experimental procedures, characterization of compounds, 1H, 13C, 19F NMR spectra, and data on DFT calculations. [file Beilstein_J_Org_Chem-12-2125-s001.pdf]

Supporting Information

for

Superelectrophilic activation of 5-hydroxymethylfurfural and 2,5-diformylfuran: organic synthesis based on biomass-derived products

Dmitry S. Ryabukhin[1,2], Dmitry N. Zakusilo[1,3], Mikhail O. Kompanets[4], Anton A.Tarakanov[1], Irina A. Boyarskaya[2], Tatiana O. Artamonova[5], Mikhail A. Khohodorkovskiy[5], Iosyp O. Opeida[6] and Aleksander V. Vasilyev[1,2*]

Address: [1]Department of Chemistry, Saint Petersburg State Forest Technical University, Institutsky per., 5, Saint Petersburg, 194021, Russia, [2]Institute of Chemistry, Saint Petersburg State University, Saint Petersburg State University, Universitetskaya nab., 7/9, Saint Petersburg, 199034, Russia, [3]The All-Russia Scientific Research Institute of Fats, ul. Chernyakhovskogo, 10, Saint Petersburg, 191119, Russia, [4]L.M. Litvinenko Institute of Physico-Organic and Coal Chemistry of NASU, Kharkivs'ke Hgw, 50, Kiyv, 02160 , Ukraine, [5]Institute of Nanobiotechnologies, Peter the Great St. Petersburg Polytechnic University, Polytechnicheskaya ul., 29, Saint Petersburg, 195251, Russia and [6]Department of Physical Chemistry of Combustible Minerals, L.M. Litvinenko Institute of Physical Organic and Coal Chemistry of NASU, Naukova St., 3a, Lviv, 79053, Ukraine

Email: Aleksander V. Vasilyev - aleksvasil@mail.ru
*Corresponding author

Experimental procedures, characterization of compounds, ^{1}H, ^{13}C, ^{19}F NMR spectra, and data on DFT calculations

Contents

General information, experimental procedures, characteristics of zeolites, characterization of compounds **3a–o**, **4a–e**, **5a–g**......S2

^{1}H and ^{13}C NMR Spectra of compounds **3a–o**, **4a–e**, **5a–g**......S11

^{1}H and ^{13}C NMR Spectra of cations **A**, **D** in TfOH and their comparison with the spectra of neutral initial compounds **1a** and **2**......S33

MALDI-MS data for oligomers obtained under the reaction of 2,5-DFF with ortho- and meta-xylenes in TfOH......S37

Details of DFT-calculations of compounds **1, 2** and cations **A**, **B**, **C**, **D**......S39

General information, experimental procedures, characterization of compounds 3a–o, 4a–e, 5a–g

The NMR spectra of compounds solutions in $CDCl_3$ were recorded on a Bruker AM-500 spectrometer at 25 °C (at 500 and 125 MHz for ^1H and ^{13}C NMR spectra, respectively). The residual proton solvent peak $CDCl_3$ (δ 7.26 ppm) for ^1H NMR spectra and the carbon signal of $CDCl_3$ (δ 77.0 ppm) for ^{13}C NMR spectra were used as references. NMR spectra in TfOH were recorded on a Bruker AVANCE III 400 spectrometer (at 400 and 100 MHz for ^1H and ^{13}C NMR spectra, respectively) using CH_2Cl_2 as internal standard. High resolution mass spectra (HRMS) were measured on a Bruker maXis HRMS-ESI-QTOF mass spectrometer and a Varian 902-MS MALDI one in the positive mode equipped with a 9.4 Tesla superconducting magnet. Chromatography–mass spectrometry data were obtained with a G2570A GC/MSD Agilent Technologies 6850c with a column HP-5MS (3 m × 0.25 mm), a thickness of the stationary phase of 0.25 μm. The preparative reactions were monitored by thin layer chromatography carried out on silica gel plates (Silufol UV-254) using UV light for detection. Column chromatography was performed on silica gel Merck 60 (0.040–0.063 mm) with hexanes/ethyl acetate mixture elution. Zeolite H-USY, CBV-500 and CBV-720 were purchased from Zeolyst Int. (http://www.zeolyst.com).

X-ray crystallography. Single crystal X-ray analysis was performed with a single crystal diffractometer, Agilent Technologies (Oxford Diffraction) «Supernova». A suitable crystal was selected and kept at 100(2) K during data collection. Using Olex2 [1], the structure was solved with the ShelXS [2] structure solution program using Direct Methods and refined with the ShelXL refinement package using Least Squares minimization.

CCDC 1483523 **5a**, CCDC–1483524 **5c** contains the supplementary crystallographic data, which can be obtained free of charge at www.ccdc.cam.ac.uk/conts/retrieving.html or from the Cambridge Crystallographic Data Centre, 12 Union Road, Cambridge CB2 1EZ, UK; Fax: (internat.) + 44-1223-336-033; E-mail: deposit@ccdc.cam.ac.uk.

DFT calculations. All computations were carried out at the DFT/HF hybrid level of theory using Becke's three-parameter hybrid exchange functional in combination with the gradient-corrected correlation functional of Lee, Yang, and Parr (B3LYP) by using GAUSSIAN 2009 program packages[3]. The geometry optimizations were performed using the 6-311+G(2d,2p) basis set

[1] O. V. Dolomanov, L. J. Bourhis, R. J. Gildea, J. A. K. Howard, H. Puschmann, *J. Appl. Cryst*., **2009**, *42*, 339-341.
[2] G. M. Sheldrick, *Acta Cryst.* **2008**, *A64*, 112-126.
[3] M. J. Frisch, G. W. Trucks, H. B. Schlegel, G. E. Scuseria, M. A. Robb, J. R. Cheeseman, G. Scalmani, V. Barone, B. Mennucci, G. A. Petersson, H. Nakatsuji, M. Caricato, X. Li, H. P.

(standard 6-311 basis set added with polarization (d,p) and diffuse functions). Optimizations were performed on all degrees of freedom and solvent-phase optimized structures were verified as true minima with no imaginary frequencies. The Hessian matrix was calculated analytically for the optimized structures in order to prove the location of correct minima and to estimate the thermodynamic parameters. Solvent-phase calculations used the Polarizable Continuum Model (PCM).

5-Chloromethylfurfural (**1b**) and 5-bromomethylfurfural (**1c**) were obtained from D-fructose according to the literature procedure[4].

General procedure for the reaction of furans 1a–c, 2 with arenes in TfOH (Tables 3–5). Furan **1a–c** or **2 (**1 mmol) was added to the mixture of TfOH (2 mL) and arene [benzene (1 mL, see Tables 3 and 5) and other arenes (1.2–10 mmol, see Table 4). The reaction mixture was stirred at room temperature for the time indicated in Tables 3–5. The mixture was poured into water (30 mL), and extracted with chloroform (3 × 30 mL). The combined extracts were washed with water, saturated aqueous solution of $NaHCO_3$, water again, and dried over Na_2SO_4. The solvent was removed under reduced pressure, and the residue was subjected to chromatographic separation on silica gel using hexanes–ethyl acetate as an eluent.

In the same manner the reactions were carried out in H_2SO_4 for **1a** (entry 5, Table 3) and **2** (entry 3, Table 5) and quenched as described above.

Reaction of **2** with benzene was carried out in HSO_3F (2 mL) and SO_2 (1.5 mL) at −45 °C for 2 h (entry 4, Table 5), followed by quenching with concentrated aqueous hydrochloric acid frozen down to −80 °C (30 mL), then diluted with water (50 mL). Extraction and work-up as described above.

Yields of the obtained compounds **3a–o**, **4a–e**, **5a** are given in Tables 3–5.

Hratchian, A. F. Izmaylov, J. Bloino, G. Zheng, J. L. Sonnenberg, M. Hada, M. Ehara, K. Toyota, R. Fukuda, J. Hasegawa, M. Ishida, T. Nakajima, Y. Honda, O. Kitao, H. Nakai, T. Vreven, J. A. Montgomery, Jr., J. E. Peralta, F. Ogliaro, M. Bearpark, J. J. Heyd, E. Brothers, K. N. Kudin, V. N. Staroverov, T. Keith, R. Kobayashi, J. Normand, K. Raghavachari, A. Rendell, J. C. Burant, S. S. Iyengar, J. Tomasi, M. Cossi, N. Rega, J. M. Millam, M. Klene, J. E. Knox, J. B. Cross, V. Bakken, C. Adamo, J. Jaramillo, R. Gomperts, R. E. Stratmann, O. Yazyev, A. J. Austin, R. Cammi, C. Pomelli, J. W. Ochterski, R. L. Martin, K. Morokuma, V. G. Zakrzewski, G. A. Voth, P. Salvador, J. J. Dannenberg, S. Dapprich, A. D. Daniels, O. Farkas, J. B. Foresman, J. V. Ortiz, J. Cioslowski, D. J. Fox, *Gaussian 09, Revision C.01*, Gaussian, Inc., Wallingford CT, **2010**.

[4] N. Kumari, J.K. Olesen, C.M. Pedersen, M. Bols, *Eur. J. Org. Chem.*, **2011**, 1266-1270.

General procedure for the reaction of furans 1a, 2 with arenes under the action of AlX_3 (X = Cl, Br) (Tables 3 and 5). Furan **1a** or **2** (1 mmol) was added to the mixture of AlX_3 (X = Cl, Br) (5 mmol) and arene (5 mL). The reaction mixture was stirred at room temperature for 1 h, then poured into water (50 mL) and extracted with chloroform (3 × 30 mL). The combined extracts were washed with water, saturated aqueous solution of $NaHCO_3$, water again, and dried over Na_2SO_4. The solvent was distilled off under reduced pressure, and the residue was subjected to chromatographic separation on silica gel using hexanes–ethyl acetate as an eluent.

Yields of the obtained compounds **5a–g** are given in Table 5.

General procedure for the reactions of furans 1a,b and 2 with arenes under the action of H-USY zeolites (CBV-500 and CBV-720) (Tables 3 and 5).

H-USY zeolite, CBV-500 or CBV-720, (Si/Al = 5.2 and 13, respectively) was activated at 550 °C for 4 h under air (see estimation of acidity below in Tables S1–S3 and ref.[5]). The activated zeolite (0.45 g), furan **1a,b** or **2** (0.37 mmol) and benzene (5 mL), toluene (5 ml) or another arene (in amounts of 2.5–4 equiv in 5 mL CS_2, see Table 4) were loaded in a 15 mL high pressure glass tube. The resulting suspension was magnetically stirred at 130 °C for 1–10 h (see time in Tables 3–5). After cooling, the reaction mixture was filtered to remove the zeolite catalyst. The zeolite was boiled with MeOH (3 × 10 mL) and filtered. The solutions were combined and the solvent distilled off under reduced pressure and the residue was subjected to chromatographic separation on silica gel using hexane–ethyl acetate as an eluent.

Characteristics of zeolites

Table S1: Characteristics of zeolites CBV-500 and CBV-720 from manufacturer Zeolyst Int. (http://www.zeolyst.com).

Zeolyst Products	SiO_2/Al_2O_3 Mole Ratio	Nominal Cation Form	Na_2O Weight %	Unit Cell Size, Å	Surface Area, m^2/g
CBV 500	5.2	Ammonium	0.2	24.53	750
CBV 720	30	Hydrogen	0.03	24.28	780

[5] N. Fonseca, F. Lemos, S. Laforge, P. Magnoux, F. Ramoa Ribeiro. *Reac. Kinet. Mech. Cat.* **2010**, 100, 249-263.

Table S2: Physicochemical properties of zeolites CBV-500 and CBV-720.[5]

Catalyst	Unit cell formula	Bulk Si/Al ratio	Crystals size (μm)	Porosity (cm^3g^{-1})	
				v_{micro}	v_{meso}
CBV-500	$Na_{0.4}H_{29.6}Al_{30}Si_{162}O_{384}$, 22.7 EFAL	2.6	0.5	0.239	0.117
CBV-720	$Na_{0.3}H_{11.0}Al_{11.3}Si_{181}O_{384}$, 7.3 EFAL	13	0.5	0.317	0.190

Table S3: Zeolite sample acidities measured by pyridine desorption between 150 and 450°C.[5]

Acidity (μmol g^{-1}): T (°C)	CBV-500		CBV-720	
	Brønsted acid sites	Lewis acid sites	Brønsted acid sites	Lewis acid sites
150	672	239	271	101
250	594	160	220	69
350	423	129	75	46
450	195	110	13	26

Yields of the obtained compounds **3a–c**,**f–n**, **5a** are given in Tables 3–5.

5-(Phenylmethyl)furan-2-carbaldehyde (3a).[5] Oil. ^{1}H NMR (500 MHz, $CDCl_3$) *δ* 9.52 (s, 1H, CHO), 7.23-7.33 (m, $5H_{arom.}$), 7.15 (d, *J* = 3.0 Hz, 1H), 6.17 (d, *J* = 3.0 Hz, 1H), 4.04 (s, CH_2, 2H). ^{13}C NMR (125 MHz, $CDCl_3$) *δ* 177.2, 162.1, 152.2, 136.1, 128.9, 128.8, 127.1, 109.8, 34.9. GC-MS ($I_{rel.}$,%): 186 M^+ (100), 157 (85), 128 (70), 115 (15).

5-[(4-Methylphenyl)methyl]furan-2-carbaldehyde (3b)[6,7] and **5-[(2-methylphenyl)methyl]furan-2-carbaldehyde (3c)**[7] were obtained as inseparable oily mixture of isomers. ^{1}H NMR (500 MHz, $CDCl_3$, data for the mixture of isomers **3b** and **3c**) *δ* 9.53 (s, CHO, 2H), 7.14-7.20 (m, $10H_{arom.}$), 6.17 (d, *J* = 3.4 Hz, 1H), 6.06 (d, *J* = 3.4 Hz, 1H), 4.05 (s, CH_2, 2H), 4.02 (s, CH_2, 2H), 2.34 (s, Me, 3H), 2.29 (s, Me, 3H). ^{13}C NMR (125 MHz, $CDCl_3$, data for the mixture of isomers **3b** and **3c**) *δ* 177.1, 162.4, 161.8, 152.1, 136.6, 136.4, 134.3, 133.0, 130.5, 129.8, 129.4, 128.7, 127.4, 126.3, 123.2, 109.7, 109.6, 34.4, 32.6, 21.0, 19.3. GC-MS ($I_{rel.}$,%, for **3b**): 200 M^+ (94), 185 (42), 171 (44), 154 (25), 141 (23), 128 (100). GC-MS ($I_{rel.}$,%, for **3c**): 200 M^+ (87), 185 (54), 171 (82), 141 (12), 128 (100).

5-[(3,4-Dimethylphenyl)methyl]furan-2-carbaldehyde (3d) and **5-(2,3-dimethylphenyl)methyl)furan-2-carbaldehyde (3e)** were obtained as inseparable oily mixture of

---

[6] a) J. Cabares, L. Mavoungou-Gomes, *Bull. Soc. Chim. France*, **1986**, 401–412; b) A. Skrzyńska, A. Przydacz, L. Albrecht, *Org. Lett.*, **2015**, *17*, 5682–5685.

[7] A. Onorato, C. Pavlik, M.A. Invernale, I.D. Berghorn, G.A. Sotzing, M.D. Mortona, M.B. Smith. *Carbohydrate Research*, **2011**, *346*, 1662–1670.

isomers. **5-[(3,4-Dimethylphenyl)methyl]furan-2-carbaldehyde (3d)**. ^{1}H NMR (500 MHz, $CDCl_3$, from the spectrum of the mixture of isomers) δ 9.54 (s, CHO, 1H), 6.98-7.16 (m, 4$H_{arom.}$), 6.18 (d, J = 3.5 Hz, 1H), 3.99 (s, CH_2, 2H), 2.24 (s, 2Me, 6H). ^{13}C NMR (125 MHz, $CDCl_3$) from the spectrum of the mixture of isomers: δ 177.1, 162.1, 152.0, 136.8, 135.2, 133.4, 130.1, 129.9, 126.2, 122.9, 109.5, 34.3, 19.6, 19.2. GC-MS ($I_{rel.}$,%): 214 M^+ (100), 199 (70), 185 (52), 168 (15), 153 (16). **5-[(2,3-Dimethylphenyl)methyl]furan-2-carbaldehyde (3e)**. ^{1}H NMR (500 MHz, $CDCl_3$, from the spectrum of the mixture of isomers) δ 9.54 (s, CHO, 1H), 6.98-7.16 (m, 4$H_{arom.}$), 6.04 (d, J = 3.5 Hz, 1H), 4.07 (s, CH_2, 2H), 2.30 (s, Me, 3H), 2.18 (s, Me, 3H). ^{13}C NMR (125 MHz, $CDCl_3$, from the spectrum of the mixture for compounds) δ 177.1, 162.1, 152.1, 137.3, 135.0, 134.2, 129.1, 127.8, 125.7, 123.8, 109.6, 33.2, 20.5, 15.2. MS ($I_{rel.}$,%): 214 M^+ (100), 199 (52), 185 (68), 153 (31). HRMS (for the mixture of isomers): calcd. for $C_{14}H_{14}O_2Na$ 237.0886 [M+Na]; found 237.0874.

5-[(2,4-Dimethylphenyl)methyl]furan-2-carbaldehyde (3f). Oil. ^{1}H NMR (500 MHz, $CDCl_3$) δ 9.53 (s, CHO, 1H), 7.14 (d, J = 3.5 Hz, 1H), 7.06 (d, J = 7.6 Hz, 1$H_{arom.}$), 7.02 (br.s, 1$H_{arom.}$), 6.98 (d, J = 7.6 Hz, 1$H_{arom.}$), 6.05 (d, J = 3.5 Hz, 1H), 4.01 (s, CH_2, 2H), 2.31 (s, Me, 3H), 2.25 (s, Me, 3H). ^{13}C NMR (125 MHz, $CDCl_3$) δ 177.0, 162.1, 152.1, 137.0, 136.2, 131.3, 129.7, 128.3, 127.1, 127.0, 109.6, 32.2, 20.9, 19.3. MS ($I_{rel.}$,%): 214 M^+ (100), 199 (80), 185 (45), 168 (25), 153 (15).). HRMS (for the mixture of isomers): calcd. for $C_{14}H_{14}O_2Na$ 237.0886 [M+Na]; found 237.0897.

5-[(2,5-Dimethylphenyl)methyl]furan-2-carbaldehyde (3g). Oil. ^{1}H NMR (500 MHz, $CDCl_3$) δ 9.54 (s, CHO, 1H), 7.15 (d, J = 3.5 Hz, 1H), 7.06-7.09 (m, 1$H_{arom.}$), 6.99-7.02 (m, 2$H_{arom.}$), 6.06 (d, J = 3.5 Hz, 1H), 4.01 (s, CH_2, 2H), 2.31 (s, Me, 3H), 2.25 (s, Me, 3H). ^{13}C NMR (125 MHz, $CDCl_3$) δ 177.1, 162.0, 152.0, 135.8, 134.1, 133.2, 130.5, 130.4, 128.0, 123.2, 109.6, 32.6, 20.8, 18.9. MS ($I_{rel.}$,%): 214 M^+ (100), 199 (50), 185 (25), 168 (22), 153 (20).). HRMS (for the mixture of isomers): calcd. for $C_{14}H_{14}O_2Na$ 237.0886 [M+Na]; found 237.0881.

5-[(2,4,5-Trimethylphenyl)methyl]furan-2-carbaldehyde (3h) and **5-[(2,3,5-trimethylphenyl)methyl]furan-2-carbaldehyde (3i)** were obtained as inseparable oily mixture of isomers. **5-[(2,4,5-Trimethylphenyl)methyl]furan-2-carbaldehyde (3h).** ^{1}H NMR (500 MHz, $CDCl_3$, from the spectrum of the mixture of isomers) δ 9.53 (s, CHO, 1H), 7.15 (d, J = 3.5 Hz, 1H), 6.97 (s, 1$H_{arom.}$), 6.95 (s, 1$H_{arom.}$), 6.07 (dd, J = 3.5, 0.6 Hz, 1H), 3.99 (s, CH_2, 2H), 2.28 (s, Me, 3H), 2.23 (s, 2Me, 6H). ^{13}C NMR (125 MHz, $CDCl_3$, data for the mixture for isomers **3h** and **3i**) δ 177.15, 177.1, 162.3, 161.9, 152.1, 135.5, 135.4, 134.7, 134.4, 134.3, 133.5, 132.6, 131.9, 131.8, 131.5, 131.2, 130.0, 128.8, 128.5, 127.7, 109.6, 109.2, 32.2, 29.3, 20.7, 20.1, 19.2, 19.1, 18.7. GC-MS ($I_{rel.}$,%): 228 M^+ (100), 213 (80), 199 (25), 182 (22), 167 (18), 156 (43). **5-[(2,3,5-Trimethylphenyl)methyl]furan-2-carbaldehyde (3i).** ^{1}H NMR (500 MHz, $CDCl_3$, from the spectrum of the mixture of isomers) δ 9.53 (s, CHO, 1H), 7.12 (d, J = 3.5 Hz, 1H), 6.94-7.04 (m,

2H$_{arom.}$), 5.91 (d, J = 3.5 Hz, 1H), 4.11 (s, CH_2, 2H), 2.23 (s, Me, 3H), 2.22 (s, Me, 3H). GC-MS ($I_{rel.}$,%): 228 M^+ (100), 213 (80), 199 (25), 182 (22), 167 (18), 156 (43). HRMS (for mixture of isomers): calcd. for $C_{15}H_{17}O_2$ 229.1223 [M+H]; found 229.1224.

5-[(3,4-Dichlorophenyl)methyl]furan-2-carbaldehyde (3j). Oil. ^{1}H NMR (500 MHz, $CDCl_3$) δ 9.55 (s, CHO, 1H), 7.39 (d, J = 8.2 Hz, 1H$_{arom.}$), 7.33 (d, J = 2.0 Hz, 1H$_{arom.}$), 7.17 (d, J = 3.5 Hz, 1H), 7.09 (dd, J = 8.2, 2.0 Hz, 1H$_{arom.}$), 6.23 (d, J = 3.5 Hz, 1H), 4.01 (s, CH_2, 2H). ^{13}C NMR (125 MHz, $CDCl_3$) δ 177.2, 160.1, 152.5, 136.3, 132.8, 131.3, 130.73, 130.69, 128.2, 122.8, 110.0, 33.9. HRMS: calcd. for $C_{12}H_9Cl_2O_2$ 254.9974 [M+H]; found 254.9978.

5-[(2,3-Dichlorophenyl)methyl]furan-2-carbaldehyde (3k) was obtained as oily mixture with its isomer **(3j)**. ^{1}H NMR (500 MHz, $CDCl_3$, from spectrum of the mixture of isomers) δ 9.54 (s, CHO, 1H), 7.16-7.40 (m, 4H$_{arom.}$), 6.22-6.23 (m, 1H), 4.22 (s, CH_2, 2H). HRMS: calcd. for $C_{12}H_9Cl_2O_2$ 254.9974 [M+H]; found 254.9978.

5-[(4-Methoxyphenyl)methyl]furan-2-carbaldehyde (3l).[8] Oil. ^{1}H NMR (500 MHz, $CDCl_3$) δ 9.54 (s, CHO, 1H), 7.15-7.18 (m, 3H$_{arom.}$), 6.86-6.88 (2H$_{arom.}$), 6.15-6.16 (m, 1H), 4.00 (s, CH_2, 2H), 3.80 (s, 3H, MeO). ^{13}C NMR (125 MHz, $CDCl_3$) δ 177.1, 162.6, 157.7, 147.8,129.9, 128.6, 121.9, 114.2, 109.5, 55.3, 34.0. GC-MS ($I_{rel.}$,%): 216 M^+ (100), 187 (87), 144 (35), 128 (25), 115 (44).

5-[(2-Methoxyphenyl)methyl]furan-2-carbaldehyde (3m) was obtained as oily mixture with its isomer **(3l).** ^{1}H NMR (500 MHz, $CDCl_3$, from spectrum of the mixture of compounds) δ 9.88 (s, CHO, 1H), 6.84-7.84 (m, 5H$_{arom.}$), 6.14 (d, J = 3.4 Hz, 1H), 3.88 (s, 3H, MeO), 3.86 (s, CH_2, 2H). GC-MS ($I_{rel.}$,%): 216 M^+ (100), 187 (87), 144 (35), 128 (25), 115 (44). HRMS: calcd. for $C_{13}H_{12}O_3Na$ 239.0679 [M+Na]; found 239.0682.

5-[(3,4-Dimethoxyphenyl)methyl]furan-2-carbaldehyde (3n). Oil. ^{1}H NMR (500 MHz, $CDCl_3$) δ 9.53 (s, CHO, 1H), 7.15 (d, J = 3.4 Hz, 1H), 6.76-6.81 (m, 3H$_{arom.}$), 6.17 (d, J = 3.4 Hz, 1H), 3.99 (s, CH_2, 2H), 3.86 (s, 3H, MeO), 3.85 (s, 3H, MeO). ^{13}C NMR (125 MHz, $CDCl_3$) δ 177.2, 162.4, 152.1, 149.1, 148.1, 128.5, 123.2, 121.0, 112.1, 111.4, 109.6, 55.9, 55.8, 34.5. MS ($I_{rel.}$,%): 246 M^+ (100), 231 (15), 215 (60), 203 (8), 115 (12). HRMS: calcd. for $C_{14}H_{14}O_4Na$ 269.0784 [M+Na]; found 269.0775.

5-[(2,4,6-Trimethylphenyl)methyl]furan-2-carbaldehyde (3o). Oil. ^{1}H NMR (500 MHz, $CDCl_3$) δ 9.52 (s, CHO, 1H), 7.10 (d, J = 3.5 Hz, 1H), 6.89 (s, 2H$_{arom.}$), 5.91 (d, J = 3.5 Hz, 1H), 4.04 (s, CH_2, 2H), 2.28 (s, Me, 3H), 2.26 (s, 2Me, 6H). ^{13}C NMR (125 MHz, $CDCl_3$) δ 177.2, 160.1, 152.5, 136.3, 132.8, 131.3, 130.73, 130.69, 128.2, 122.8, 110.0, 33.9. MS ($I_{rel.}$,%): 228 M^+ (100), 213 (48),

[8] A. Skrzyńska, A. Przydacz, L. Albrecht, *Org. Lett.*, **2015**, *17*, 5682–5685.

195 (22), 182 (21), 167 (18), 156 (32). HRMS (for mixture of isomers): calcd. for $C_{15}H_{17}O_2$ 229.1223 [M+H]; found 229.1214.

Compounds *i*-**4a** were obtained as a mixture of isomers with predominance of *p*-methylphenyl substituted furan.

2-[(4-Methylphenyl)methyl]-5-[bis(4-methylphenyl)methyl]-furan (4a). Oil. ^{1}H NMR (500 MHz, $CDCl_3$, from the spectrum of the mixture of isomers) δ 7.03-7.16 (m, 12H$_{arom.}$), 5.85 (s, 1H), 5.76 (s, CH, 1H), 5.34 (s, 1H), 3.89 (s, CH_2, 2H), 2.33 (s, 3Me, 9H). ^{13}C NMR (125 MHz, $CDCl_3$, from the spectrum of the mixture of isomers) δ 155.8, 154.2, 139.3, 136.0, 129.1, 129.0, 128.6, 108.8, 106.5, 50.1, 34.1, 21.0. MS (I$_{rel.}$,%): 366 M^+ (36), 351 (12), 275 (16), 261 (100). HRMS: calcd. for $C_{27}H_{26}ONa$ 389.1876 [M+Na]; found 389.1991.

2-[(3,4-Dimethylphenyl)methyl]-5-[bis(3,4-dimethylphenyl)methyl]furan (4b). Oil. ^{1}H NMR (500 MHz, $CDCl_3$) δ 7.05-7.07 (m, 4H$_{arom.}$), 6.98-7.00 (m, 4H$_{arom.}$), 6.91 (dd, J = 7.7, 1.7 Hz, 2H$_{arom.}$), 5.88 (d, J = 2.8 Hz, 1H), 5.79 (d, J = 2.8 Hz, 1H), 5.29 (s, CH, 1H), 3.87 (s, CH_2, 2H), 2.25 s (3H, Me), 2.25 s (6H, 2Me), 2.24 s (3H, Me), 2.22 s (6H, 2Me). ^{13}C NMR (125 MHz, $CDCl_3$) δ 155.9, 154.2, 139.8, 136.4, 136.3, 135.8, 134.6, 134.3, 130.0, 129.9, 129.6, 129.5, 126.03, 126.0, 108.7, 106.4, 50.2, 34.0, 19.8, 19.6, 19.3, 19.2. MS (I$_{rel.}$,%): 408 M^+ (35), 289 (100), 246 (8), 215 (5). HRMS: calcd. for $C_{30}H_{32}ONa$ 431.2351 [M+Na]; found 431.2364.

2-[(2,4-Dimethylphenyl)methyl]-5-[bis(2,4-dimethylphenyl)methyl]furan (4c). Oil. ^{1}H NMR (500 MHz, $CDCl_3$) δ 7.06 (d, J = 7.8 Hz, 1H$_{arom.}$), 6.93-7.00 (m, 4H$_{arom.}$), 6.90 (d, J = 7.6 Hz, 2H$_{arom.}$), 6.70 (d, J = 7.8 Hz, 2H$_{arom.}$), 5.70 (d, J = 2.5 Hz, 1H), 5.54 (d, J = 2.5 Hz, 1H), 5.52 (s, CH, 1H), 3.86 (s, CH_2, 2H), 2.30 (s, 4Me, 12H), 2.18 (s, 2Me, 6H). ^{13}C NMR (125 MHz, $CDCl_3$) δ 155.0, 153.7, 137.0, 136.0, 135.9, 133.5, 131.2, 131.1, 131.0, 129.3, 128.4, 127.2, 126.6, 126.5, 126.4, 109.5, 106.6, 48.8, 31.9, 20.9, 19.2, 19.0. MS (I$_{rel.}$,%): 408 M^+ (65), 289 (100), 246 (8), 207 (35). HRMS: calcd. for $C_{30}H_{32}ONa$ 431.2351 [M+Na]; found 431.2345.

2-[(2,5-Dimethylphenyl)methyl]-5-[bis(2,5-dimethylphenyl)methyl]furan (4d). Oil. ^{1}H NMR (500 MHz, $CDCl_3$) δ 7.05 (d, J = 7.6 Hz, 3H$_{arom.}$), 6.96 (d, J = 7.6 Hz, 3H$_{arom.}$), 6.94 (s, 1H$_{arom.}$), 6.63 (s, 2H$_{arom.}$), 5.77 (d, J = 2.8 Hz, 1H), 5.58 (d, J = 2.8 Hz, 1H), 5.55 (s, CH, 1H), 3.88 (s, CH_2, 2H), 2.29 (s, Me, 3H), 2.24 (s, Me, 3H), 2.23 (s, 2Me, 6H), 2.18 (s, 2Me, 6H). ^{13}C NMR (125 MHz, $CDCl_3$) δ 154.8, 153.5, 139.6, 135.1, 133.1, 130.1, 130.0, 129.1, 127.2, 109.7, 106.8, 44.4, 32.4, 21.2, 20.9, 18.9. MS (I$_{rel.}$,%): 408 M^+ (85), 289 (100), 246 (30), 207 (90). HRMS: calcd. for $C_{30}H_{32}ONa$ 431.2351 [M+Na]; found 431.2357.

2-[(2,4,5-Trimethylphenyl)methyl]-5-[bis(2,4,5-trimethylphenyl)methyl]furan (4e). Oil. ^{1}H NMR (500 MHz, $CDCl_3$) δ 6.96-6.97 (m, 2H$_{arom.}$), 6.93 (s, 2H$_{arom.}$), 6.64 (s, 2H$_{arom.}$), 5.78 (d, J = 3.0 Hz, 1H), 5.62 (d, J = 3.0 Hz, 1H), 5.53 (s, CH, 1H), 3.89 (s, CH_2, 2H), 2.26 (s, 5Me, 15H), 2.20 (s, 2Me, 6H), 2.17 (s, 2Me, 6H). ^{13}C NMR (125 MHz, $CDCl_3$) δ 155.2, 153.7, 137.3, 134.4, 134.3,

134.0, 133.7, 133.5, 133.47, 133.3, 131.62, 131.55, 130.7, 129.8, 109.4, 106.6, 43.7, 32.0, 19.4, 19.2, 19.1, 18.7. MS ($I_{rel.}$,%): 450 M^+ (60), 317 (100), 274 (12), 235 (35), 197 (23). HRMS: calcd. for $C_{33}H_{38}ONa$ 473.2815; found 473.2839.

5-(Diphenylmethyl)furan-2-carbaldehyde (5a). Colorless solid, m.p. 91–93°C. ^{1}H NMR (500 MHz, $CDCl_3$) δ 9.55 (s, CHO, 1H), 7.30 (t, J = 7.6 Hz, 4$H_{arom.}$), 7.24 (t, J = 3.6 Hz, 2$H_{arom.}$), 7.17 (d, J = 3.5 Hz, 1H), 7.15 (d, J = 7.6 Hz, 4$H_{arom.}$), 6.14 (d, J = 3.5 Hz, 1H), 5.55 (s, CH, 1H). ^{13}C NMR (125 MHz, $CDCl_3$) δ 177.5, 163.7, 152.4, 140.2, 128.9, 128.6, 127.1, 122.1, 111.6, 51.0. MS ($I_{rel.}$,%): 262 M^+ (58), 233 (100), 215 (20), 203 (22), 185 (21). HRMS: calcd. for $C_{18}H_{15}O_2$ 263.1067 [M+H]; found 263.1071.

5-[Bis(3,4-dimethylphenyl)methyl]furan-2-carbaldehyde (5b). Oil. ^{1}H NMR (500 MHz, $CDCl_3$) δ 9.56 (s, 1H, CHO), 7.18 (d, J = 3.4 Hz, 1H), 7.07 (d, J = 7.6 Hz, 2$H_{arom.}$), 6.94 s (2$H_{arom.}$), 6.88 (d, J = 7.6 Hz, 2$H_{arom.}$), 6.16 (d, J = 3.4 Hz, 1H), 5.40 (s, 1H, CH), 2.23 (s, 6H, 2Me), 2.21 s (6H, 2Me). ^{13}C NMR (125 MHz, $CDCl_3$) δ 177.6, 164.5, 152.3, 137.9, 136.7, 135.3, 129.8, 129.8, 125.8, 121.8, 111.2, 50.4, 19.7, 19.2. MS, m/z ($I_{rel.}$, %): 318 M^+ (52), 303 (28), 289 (100), 274 (11), 259 (9), 246 (11), 231 (7), 213 (13). HRMS: calcd. for $C_{22}H_{23}O_2$ 319.1693 [M+H]; found 319.1702.

5-[Bis(3,5-dimethylphenyl)methyl]furan-2-carbaldehyde (5c) and **5-[bis(2,4-dimethylphenyl)methyl]furan-2-carbaldehyde (5d)** were obtained as inseparable oily mixture of isomers. **5-[Bis(3,5-dimethylphenyl)methyl]furan-2-carbaldehyde (5c).** ^{1}H NMR (500 MHz, $CDCl_3$, from the spectrum of the mixture of isomers) δ 9.57 (s, 1H, CHO), 7.19 (d, J = 3.6 Hz, 1H,), 6.89 s (2$H_{arom.}$), 6.77 s (4$H_{arom.}$), 6.16 (d, J = 3.6 Hz, 1H,), 5.37 (s, 1H, CH), 2.27 (s, 12H, 4 Me). ^{13}C NMR (125 MHz, $CDCl_3$, from the spectrum of the mixture of isomers) δ 177.8, 164.4, 152.4, 140.3, 138.1, 128.8, 126.4, 121.9, 111.5, 51.1, 21.3. GC-MS, m/z ($I_{rel.}$, %): 318 M^+ (87), 303 (21), 289 (100), 246 (26), 213 (21), 141 (19). **5-[Bis(2,4-dimethylphenyl)methyl]furan-2-carbaldehyde (5d).** ^{1}H NMR (500 MHz, $CDCl_3$, from the spectrum of the mixture of isomers) δ 9.56 (s, 1H, CHO), 7.17 (d, J = 3.6Hz, 1H,), 7.08-6.91 m (6$H_{arom.}$), 6.04 (d, J = 3.6 Hz, 1H,), 5.56 (s, 1H, CH), 2.26 (s, 6H, 2Me), 2.30 (s, 6H, 2Me), 2.26 (s, 6H, 2Me). ^{13}C NMR (125 MHz, $CDCl_3$, from the spectrum of the mixture of isomers) δ 177.7, 164.6, 144.0, 139.7, 138.7, 131.5, 128.8, 128.7, 127.7, 122.4, 111.9, 47.4, 20.9, 19.5. GC-MS, m/z ($I_{rel.}$, %): 318 M^+ (77), 308 (21), 289 (100), 246 (16), 213 (20). HRMS (for mixture of isomers): calcd. for $C_{22}H_{23}O_2$ 319.1693 [M+H]; found 319.1702.

5-[Bis(2,5-dimethylphenyl)methyl]furan-2-carbaldehyde (5e). Colorless solid, m.p. 110–112°C. ^{1}H NMR (500 MHz, $CDCl_3$) δ 9.57 (s, 1H, CHO), 7.19 (d, J = 3.6 Hz, 1H), 7.06 (d, J = 7.5 Hz, 2$H_{arom.}$), 6.99 (d, J = 7.5 Hz, 4$H_{arom.}$,), 5.96 (d, J = 3.6 Hz, 1H), 5.68 (s, 1H, CH), 2.23 (s, 6H, 2Me), 2.14 (s, 6H, 2Me). ^{13}C NMR (125 MHz, $CDCl_3$) δ 177.6, 164.0, 152.4, 137.9, 135.4, 133.0, 130.4, 128.8, 127.8, 126.2, 122.4, 112.3, 44.6, 21.09, 18.8. GC-MS, m/z ($I_{rel.}$, %): 318 M^+ (100),

308 (26), 289 (50), 246 (16), 213 (25), 183 (43). HRMS: calcd. for $C_{22}H_{23}O_2$ 319.1693 [M+H]; found 319.1685.

5-[Bis(3,4-dichlorophenyl)methyl]furan-2-carbaldehyde (5f) and **5-[Bis(2,3-dichlorophenyl)methyl]furan-2-carbaldehyde (5g)** were obtained as inseparable oily mixture of isomers. **5-[Bis(3,4-dichlorophenyl)methyl]furan-2-carbaldehyde (5f).** ^{1}H NMR (500 MHz, $CDCl_3$, from the spectrum of the mixture of isomers) δ 9.59 (s, 1H, CHO), 7.42 (d, 2$H_{arom.}$ J = 8.4, 3.6 Hz), 7.22-7.17 (m, 2$H_{arom.}$), 7.22-7.17 (m, 1H, CH), 6.98 (d.d, 2$H_{arom.}$ J = 8.3, 2.1 Hz), 6.20 (d, 1H, J = 3.6 Hz), 5.44 (s, 1H, CH). ^{13}C NMR (125 MHz, $CDCl_3$, from the spectrum of the mixture of isomers) δ 177.5, 160.6, 152.9, 139.3, 133.2, 132.1, 130.9, 130.5, 127.9, 121.9, 111.9, 49.3. GC-MS, *m/z* ($I_{rel.}$, %): 400 M^+ (45), 371 (100), 306 (35), 253 (38), 236 (37), 200 (12), 162 (22). **5-[Bis(2,3-dichlorophenyl)methyl]furan-2-carbaldehyde (5g)** ^{1}H NMR (500 MHz, $CDCl_3$, from the spectrum of the mixture of isomers) δ 9.59 (s, 1H, CHO), 7.42-7.40 (m, 2$H_{arom.}$), 7.22-7.17 (m, 2$H_{arom.}$), 7.22-7.17 (m, 1H, CH), 6.90 (d.d, 2$H_{arom.}$ J = 7.8, 3.6 Hz), 6.14 (d, 1H, J = 3.6 Hz), 5.96 (s, 1H, CH). ^{13}C NMR (125 MHz, $CDCl_3$, from the spectrum of the mixture of isomers) δ 177.5, 139.2, 138.6, 130.8, 130.7, 129.9, 128.2, 128.0, 127.5, 122.5, 112.4, 47.8. GC-MS, *m/z* ($I_{rel.}$, %): 400 M^+ (57), 371 (100), 306 (33), 270 (24), 253 (35), 236 (52), 200 (30), 162 (34), 135 (17), 118 (17), 100 (18). HRMS (for mixture of isomers): calcd. for $C_{18}H_{11}Cl_4O_2$ 398.9508 [M+H]; found 398.9511.

^{1}H and ^{13}C NMR Spectra of compounds 3a–o, 4a–e, 5a–g

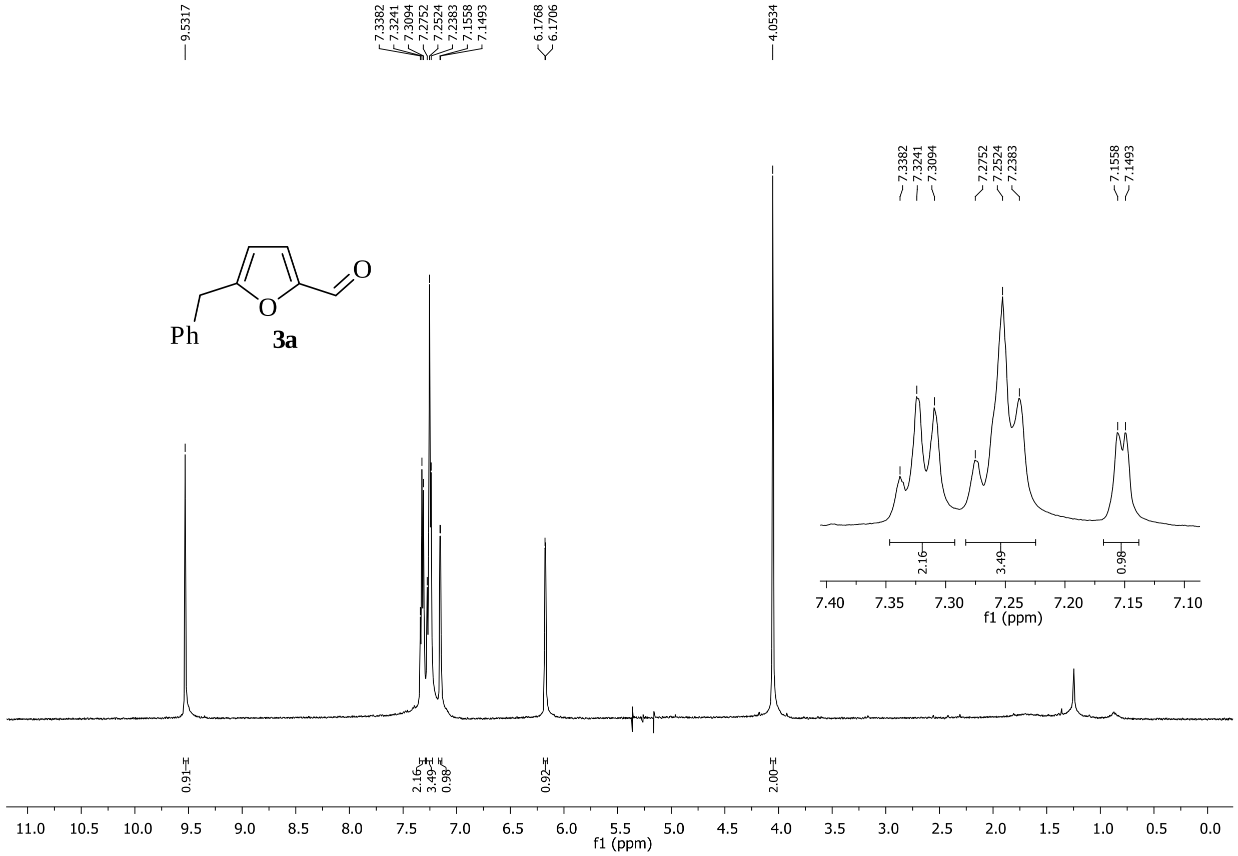

Figure S1. ^{1}H NMR spectrum of compound **3a** (500 MHz, $CDCl_3$).

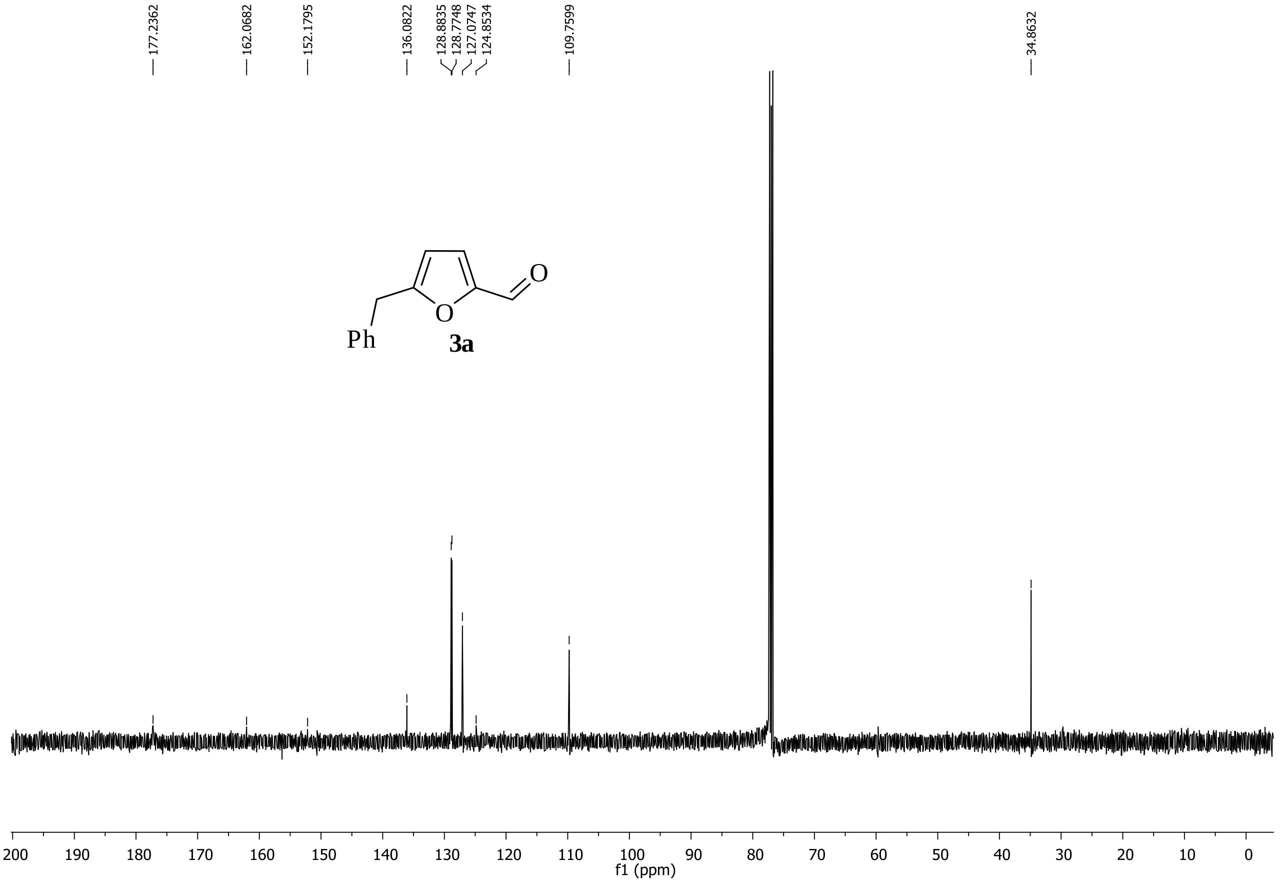

Figure S2. ^{13}C NMR spectrum of compound **3a** (125 MHz, $CDCl_3$).

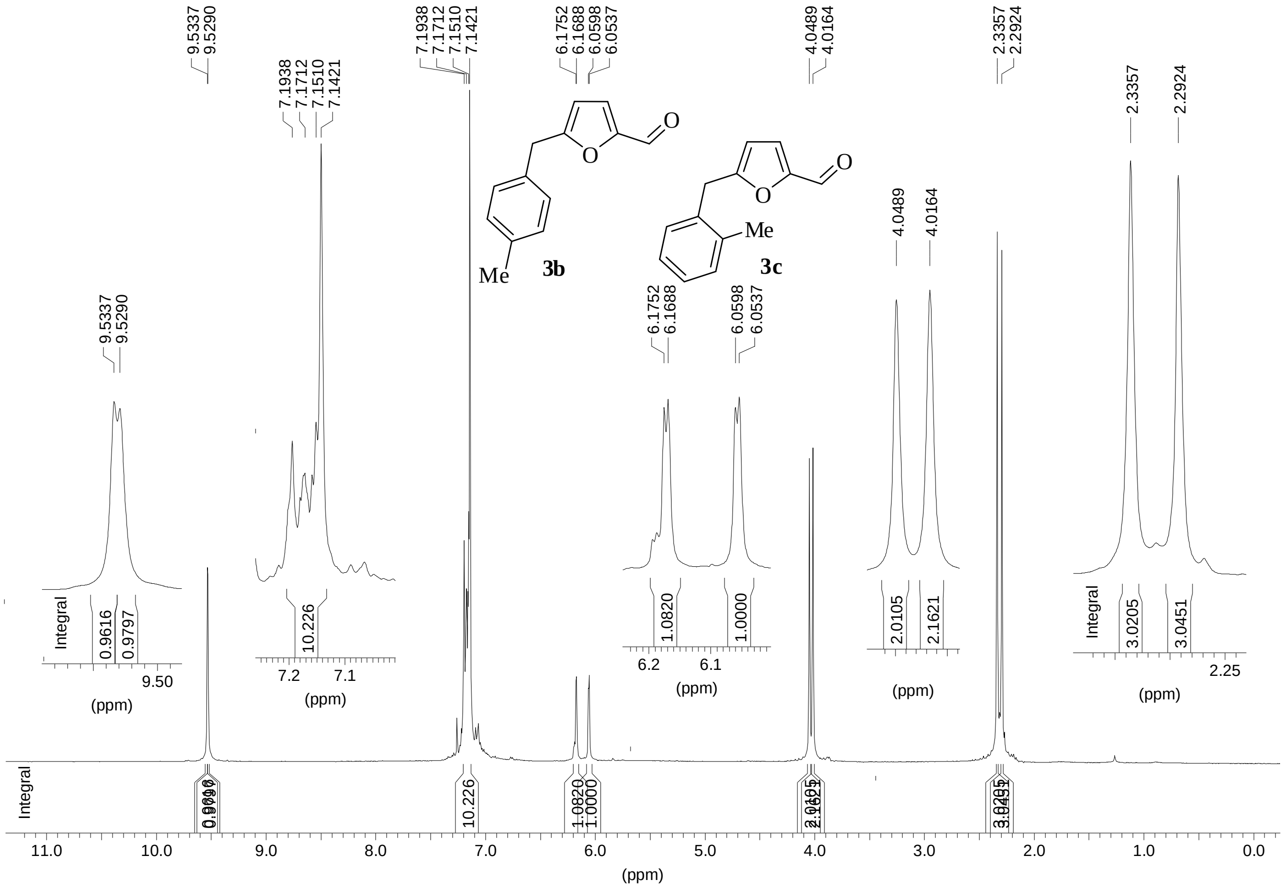

Figure S3 ^{1}H NMR spectrum of mixture of compounds **3b** and **3c** (500 MHz, $CDCl_3$).

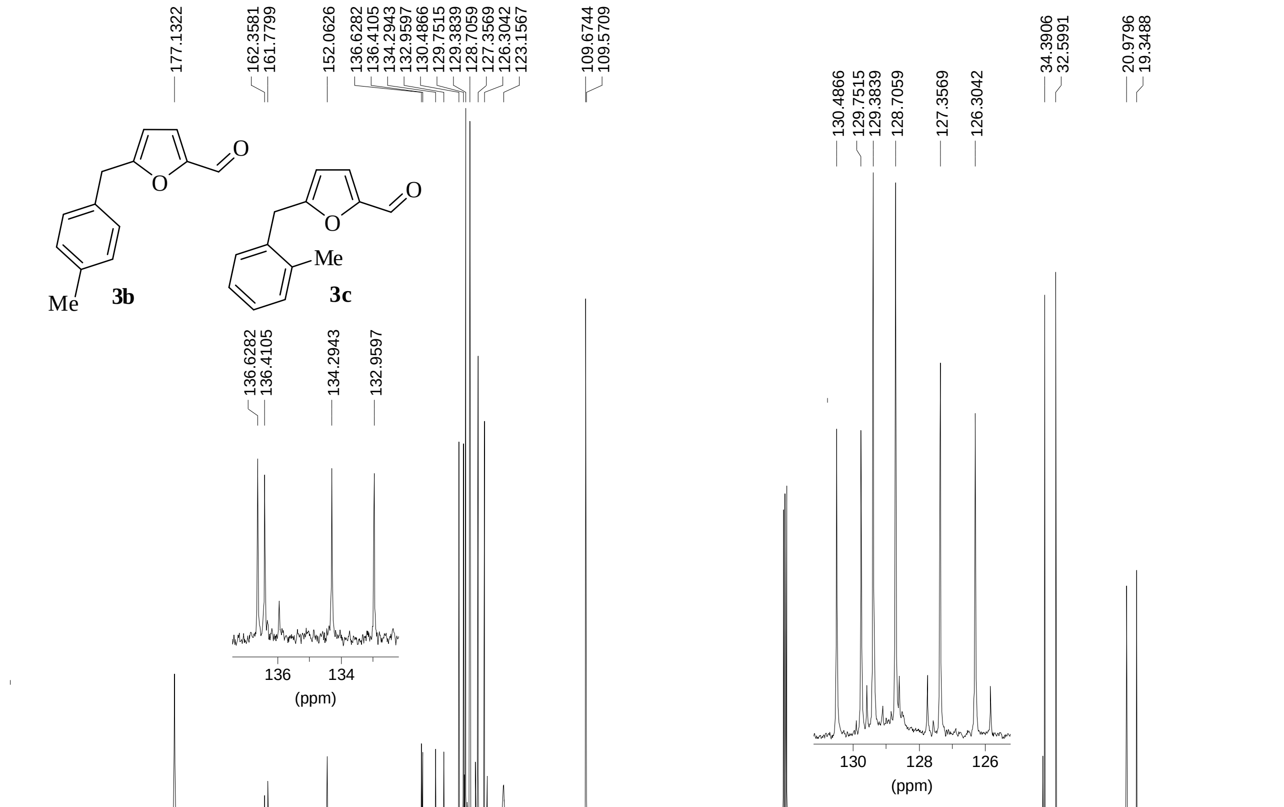

Figure S4. ^{13}C NMR spectrum of mixture of compounds **3b** and **3c** (125 MHz, $CDCl_3$).

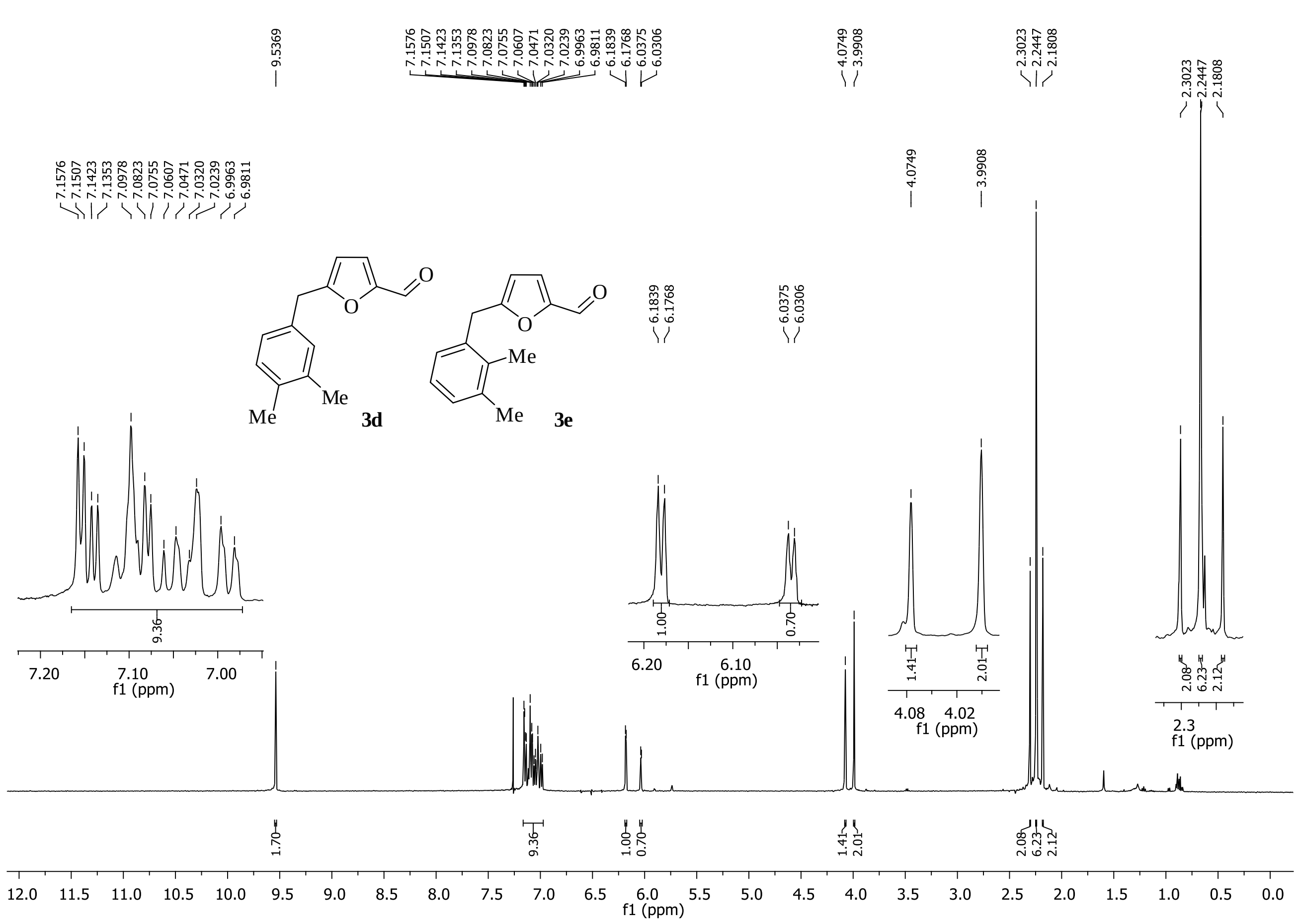

Figure S5. ^{1}H NMR spectrum of mixture of compounds **3d** and **3e** (500 MHz, $CDCl_3$).

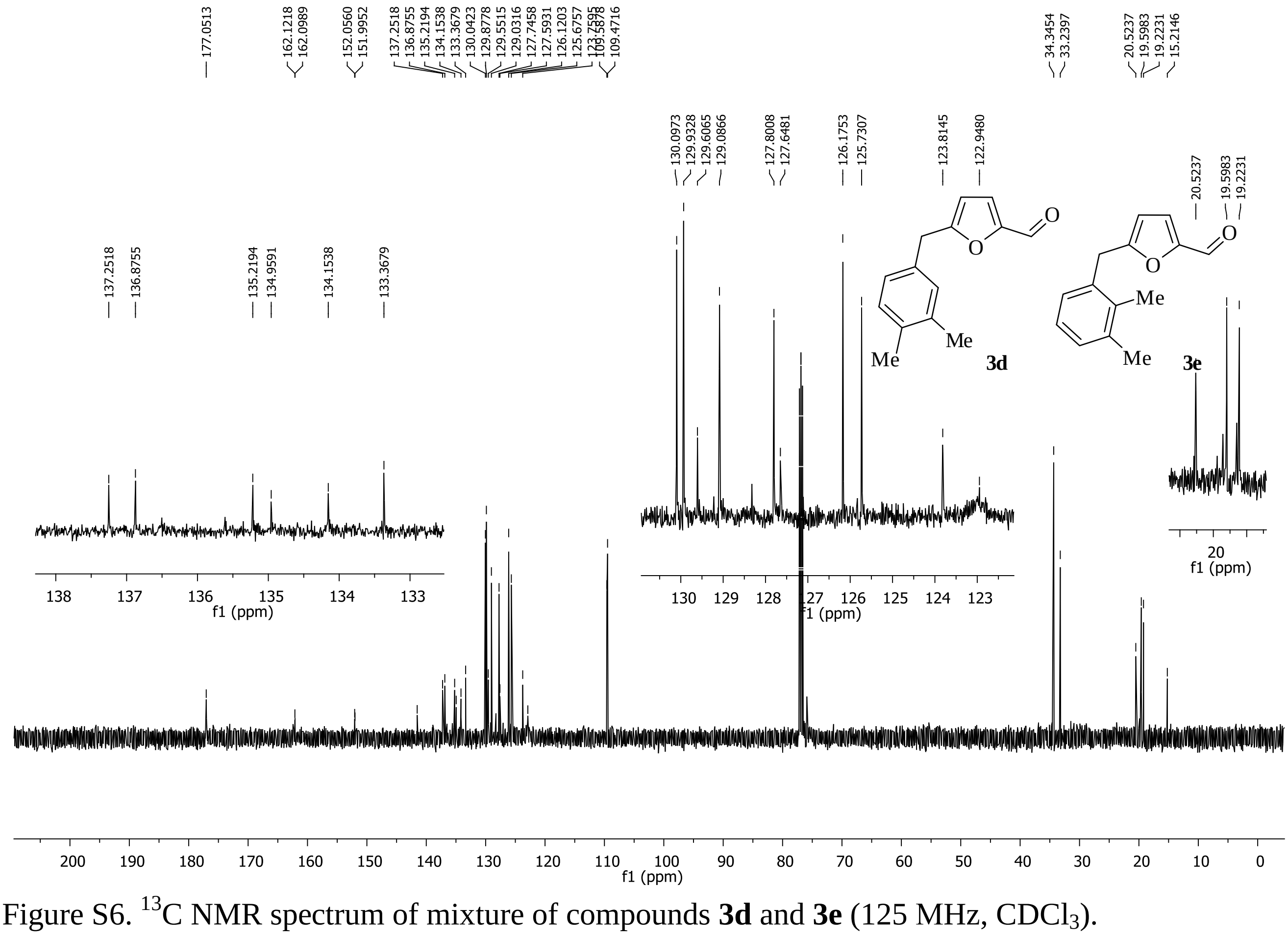

Figure S6. ^{13}C NMR spectrum of mixture of compounds **3d** and **3e** (125 MHz, $CDCl_3$).

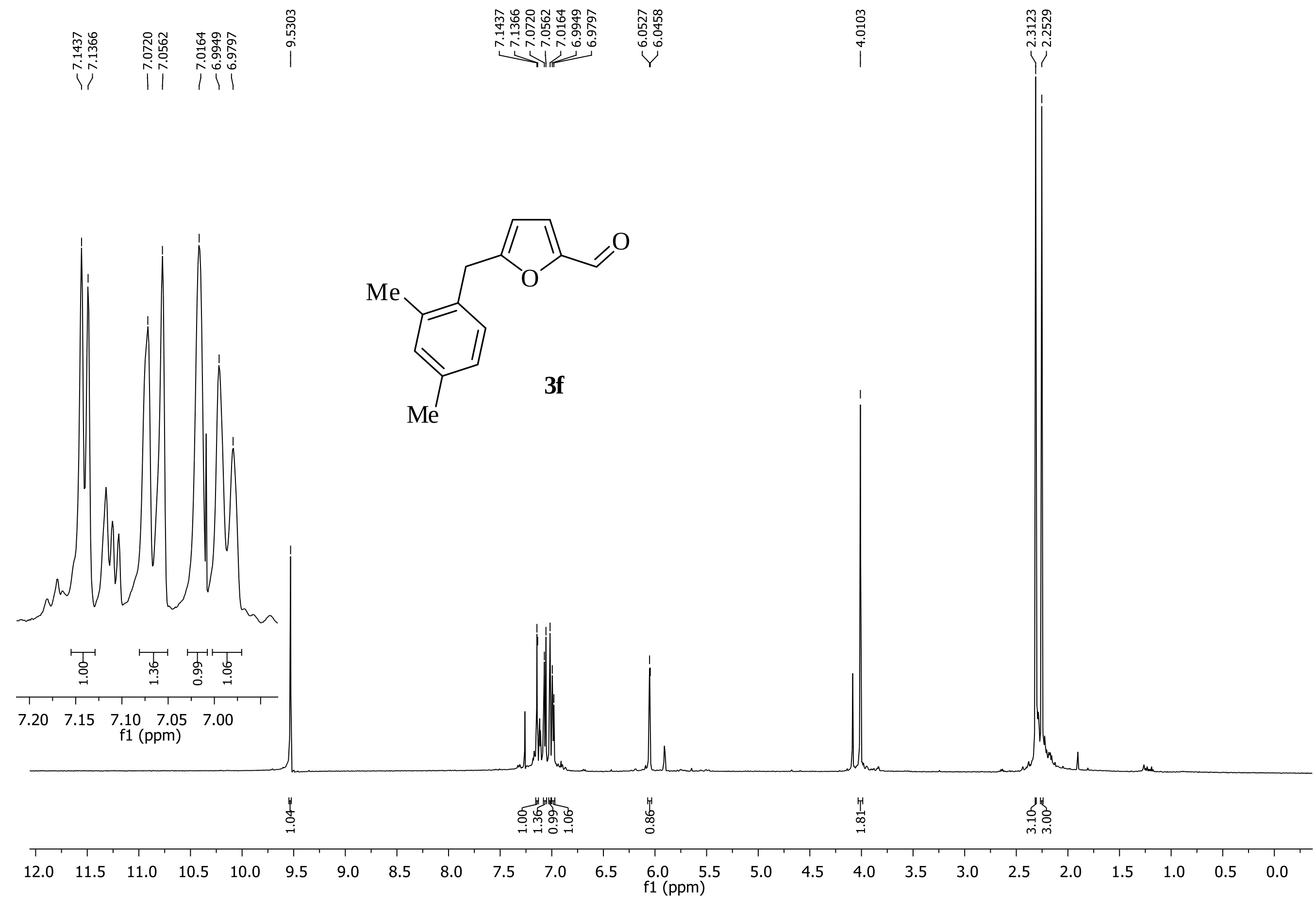

Figure S7. ^{1}H NMR spectrum of compound **3f** (500 MHz, $CDCl_3$).

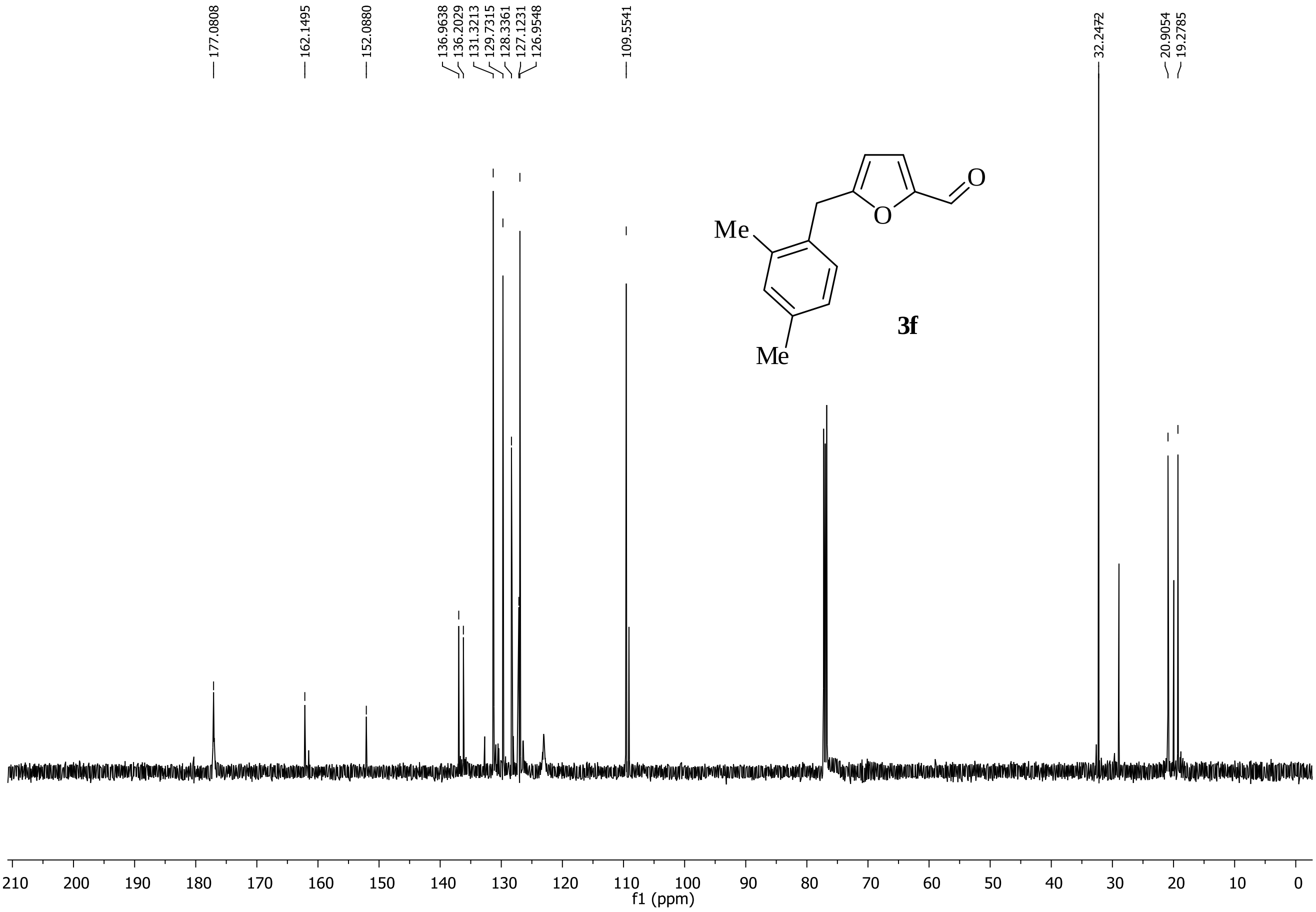

Figure S8. ^{13}C NMR spectrum of compound **3f** (125 MHz, $CDCl_3$).

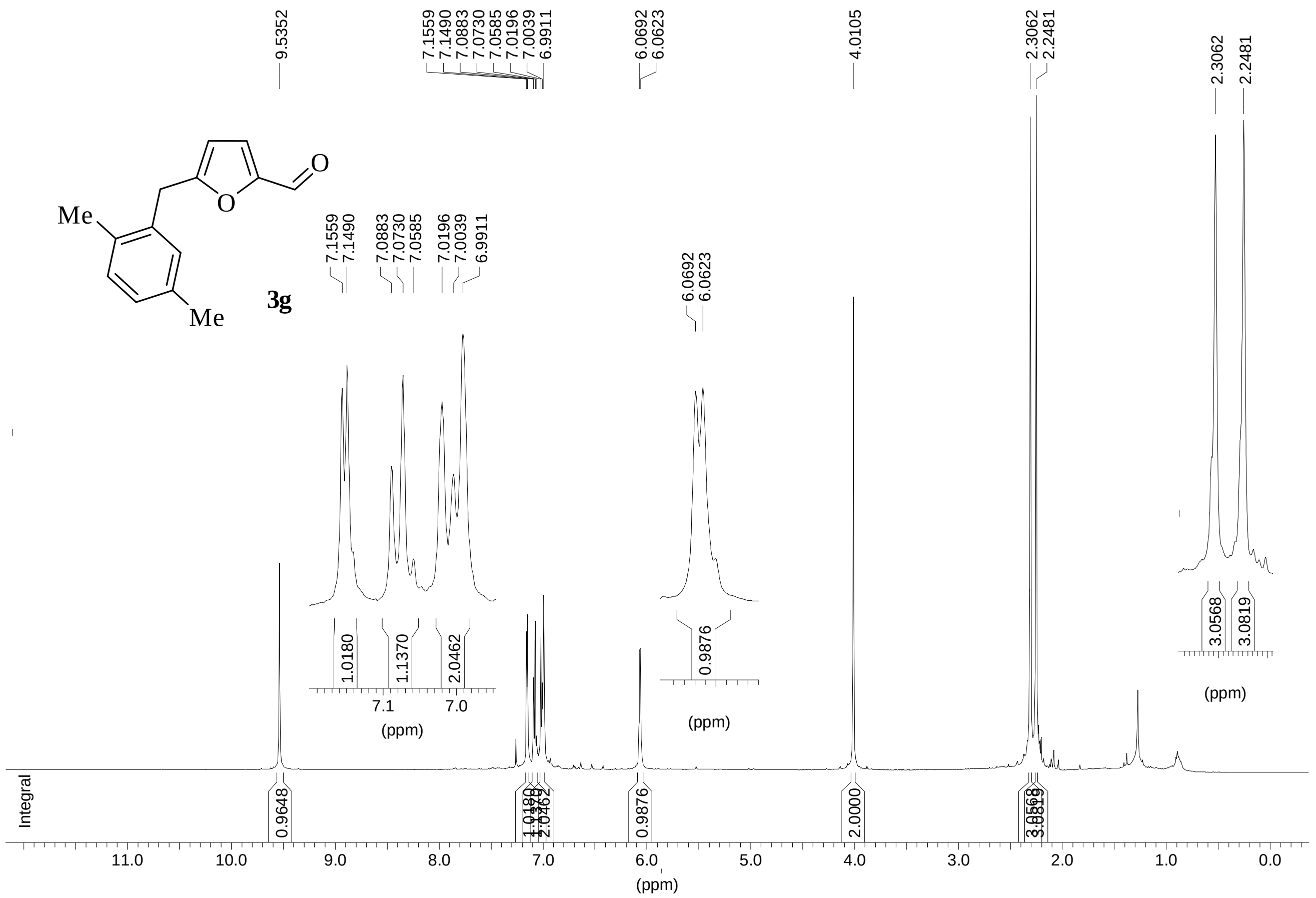

Figure S9. ^{1}H NMR spectrum of compound **3g** (500 MHz, $CDCl_3$).

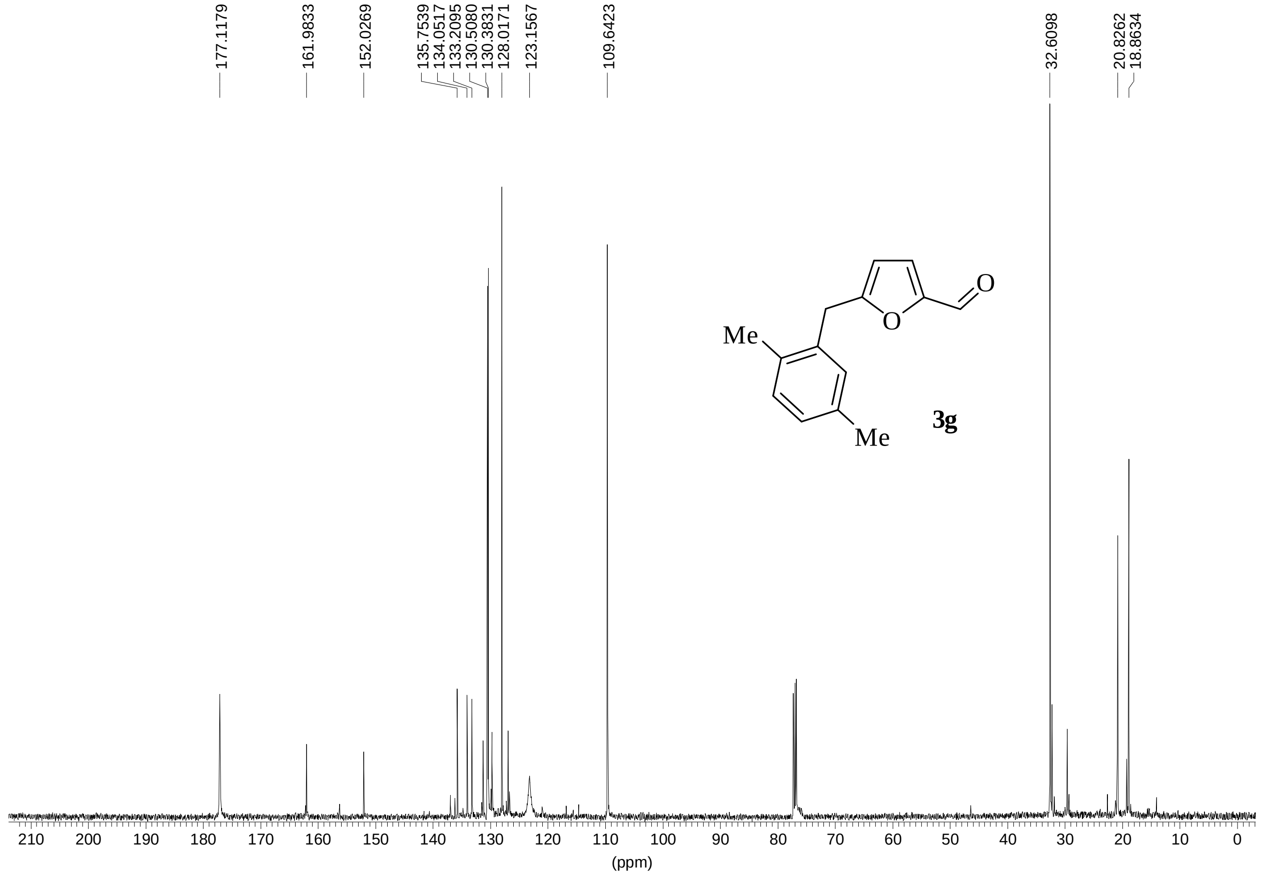

Figure S10. ^{13}C NMR spectrum of compound **3g** (125 MHz, $CDCl_3$).

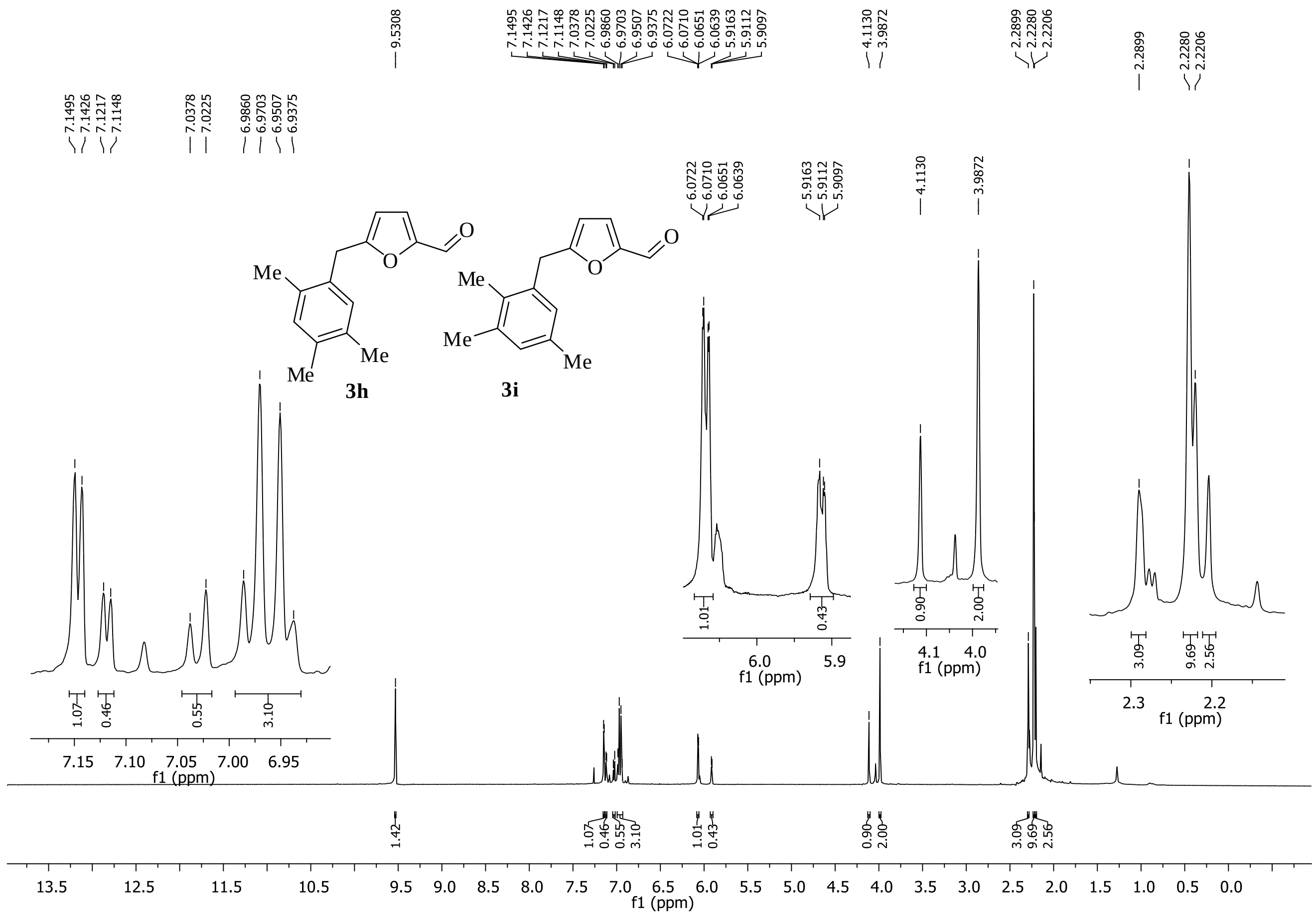

Figure S11. ^{1}H NMR spectrum of mixture of compounds **3h** and **3i** (500 MHz, $CDCl_3$).

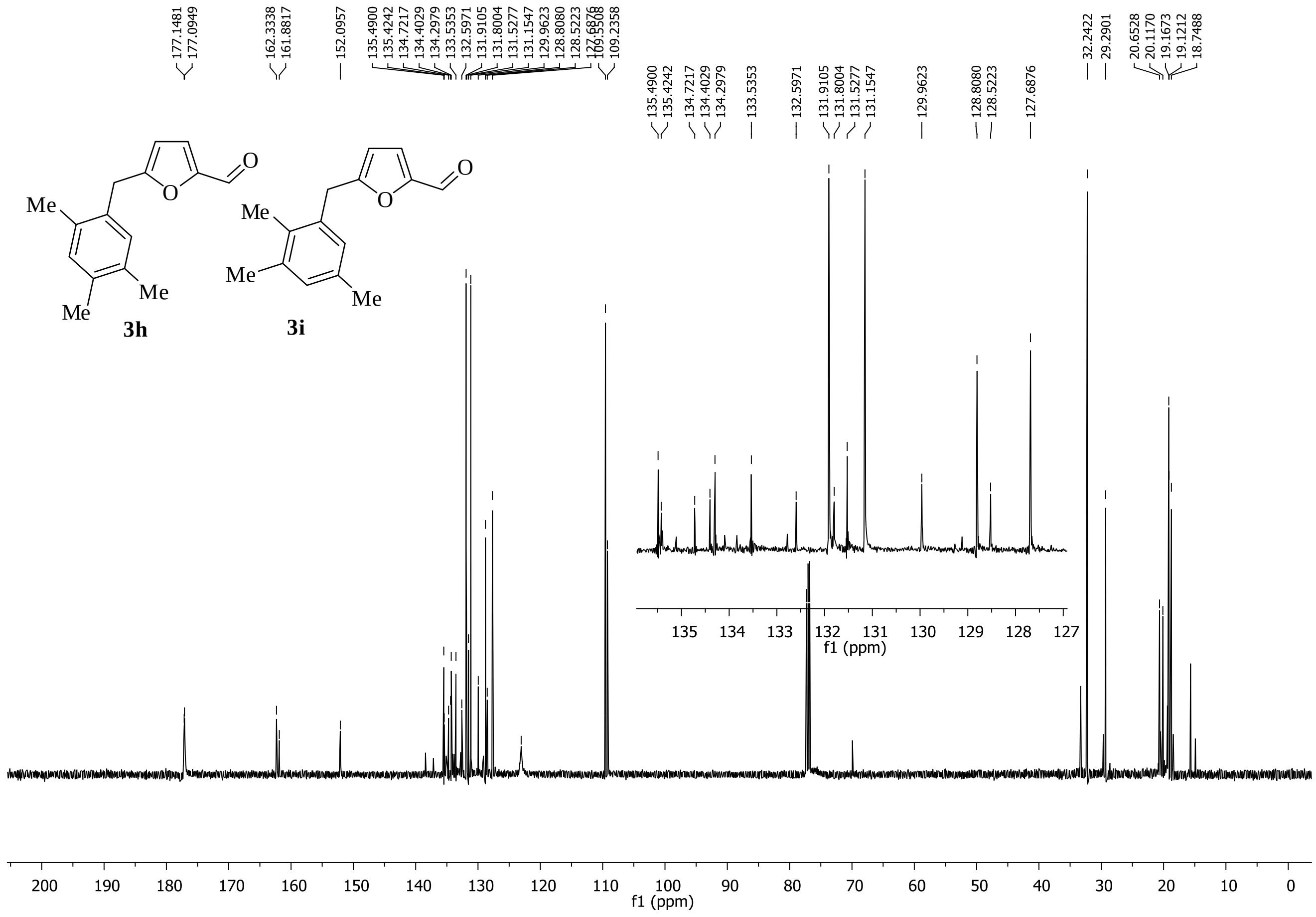

Figure S12. ^{13}C NMR spectrum of mixture of compounds **3h** and **3i** (125 MHz, $CDCl_3$)

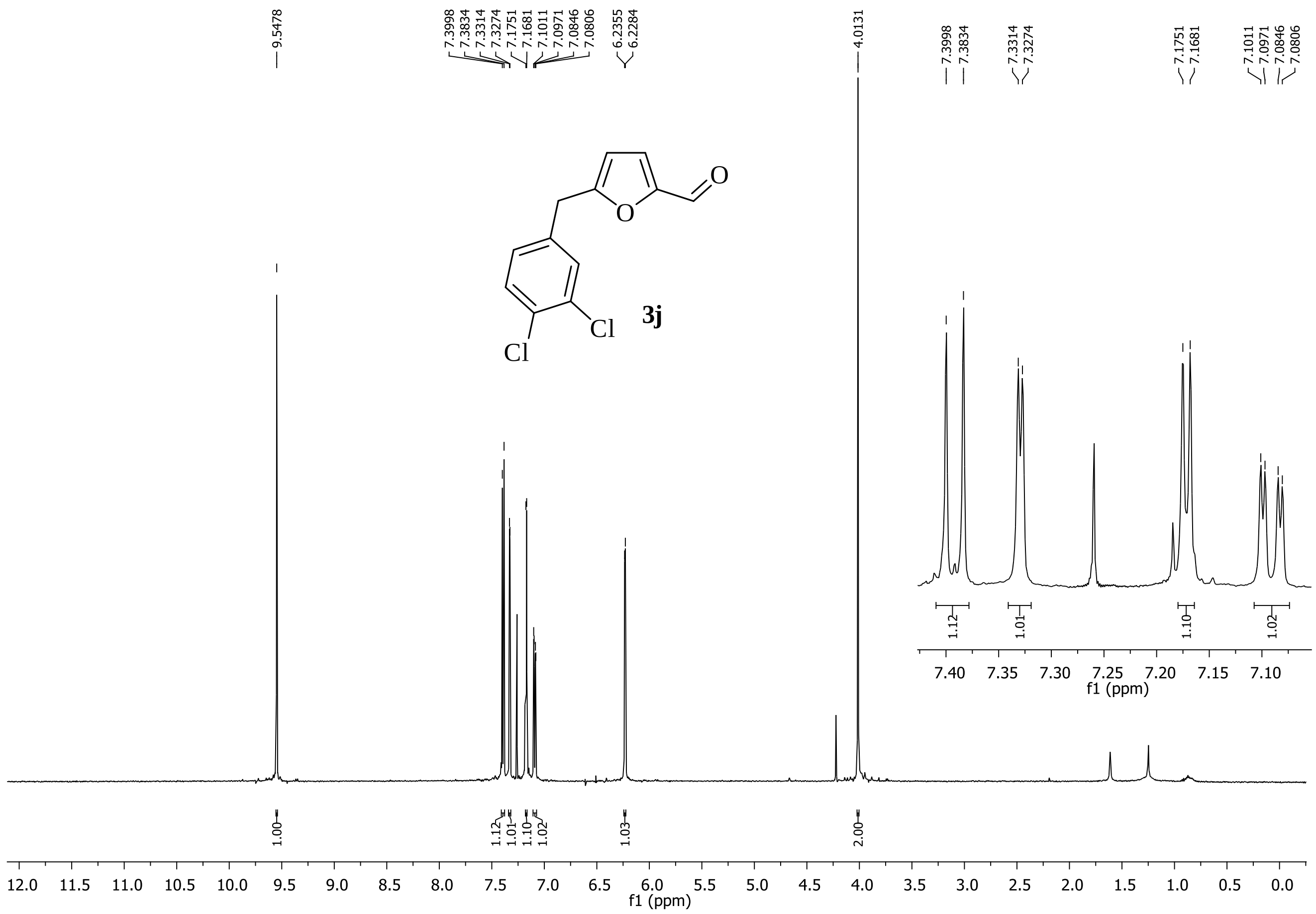

Figure S13. ^{1}H NMR spectrum of compound **3j** (500 MHz, $CDCl_3$).

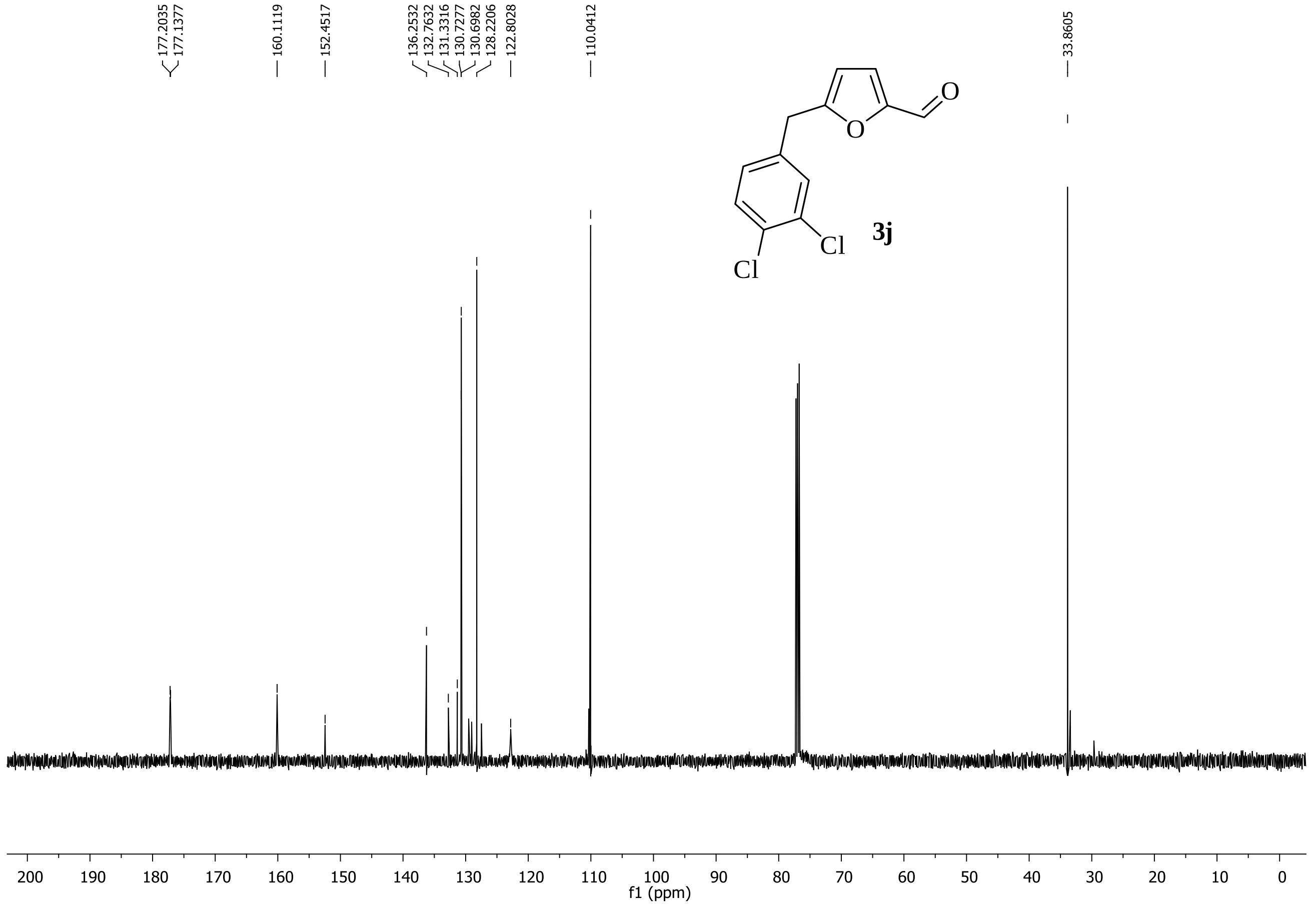

Figure S14. ^{13}C NMR spectrum of compound **3j** (125 MHz, $CDCl_3$).

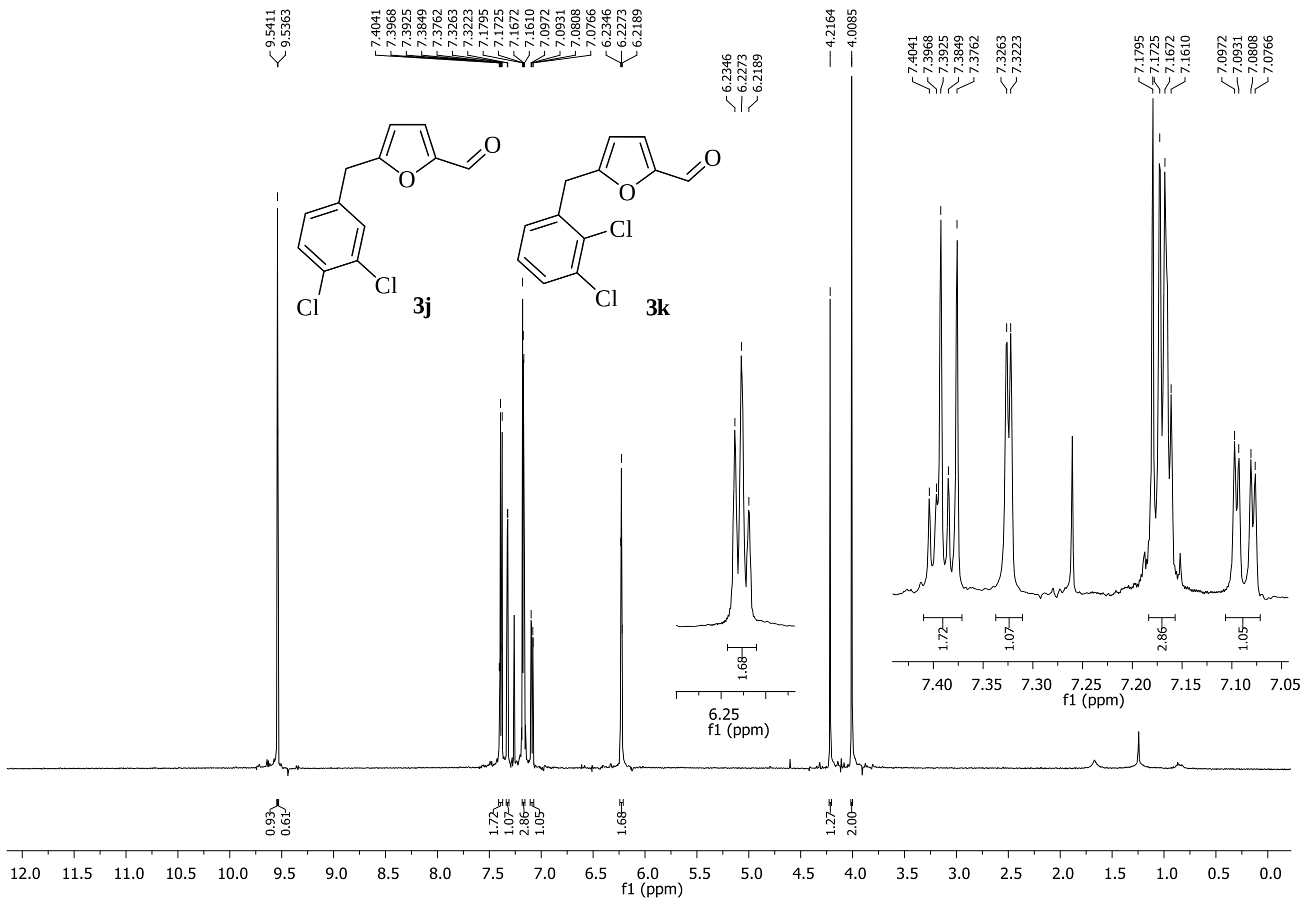

Figure S15. ^{1}H NMR spectrum of mixture of compounds **3j** and **3k** (500 MHz, $CDCl_3$).

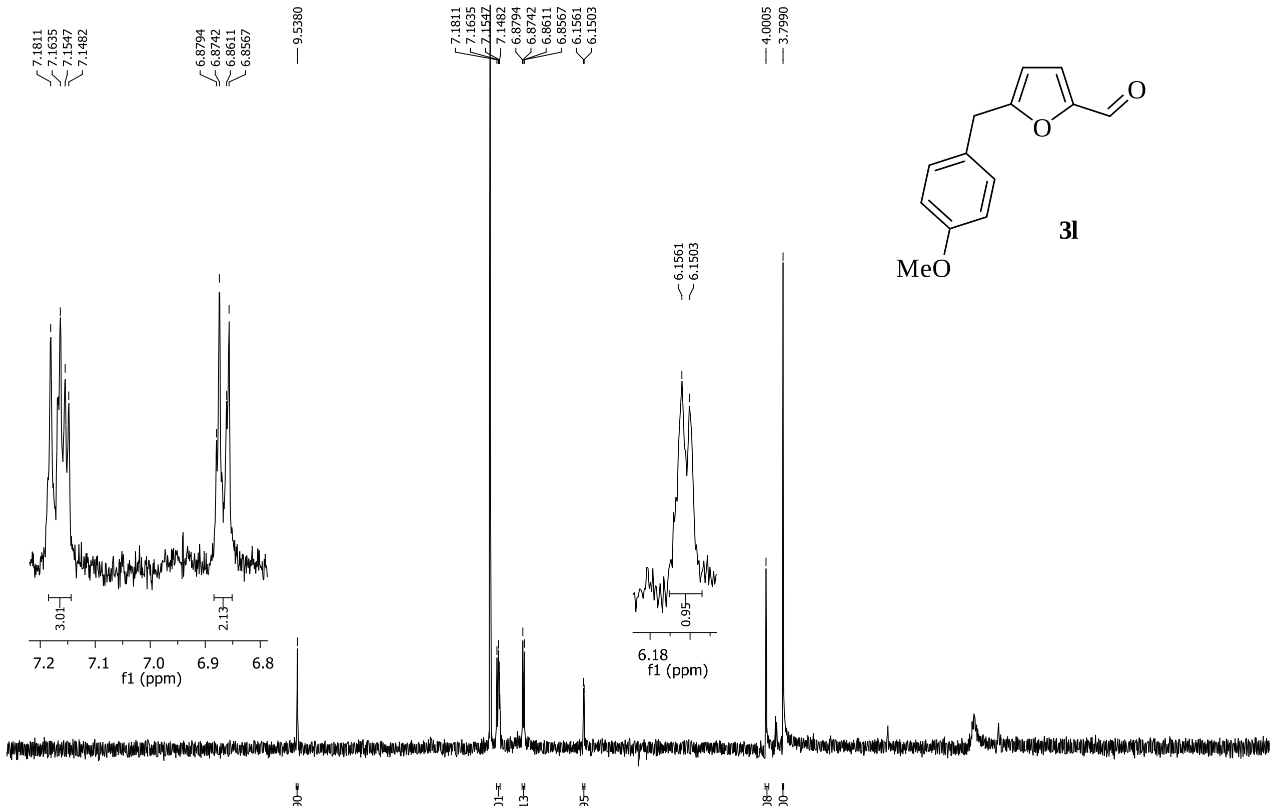

Figure S16. ^{1}H NMR spectrum of compound **3l** (500 MHz, $CDCl_3$).

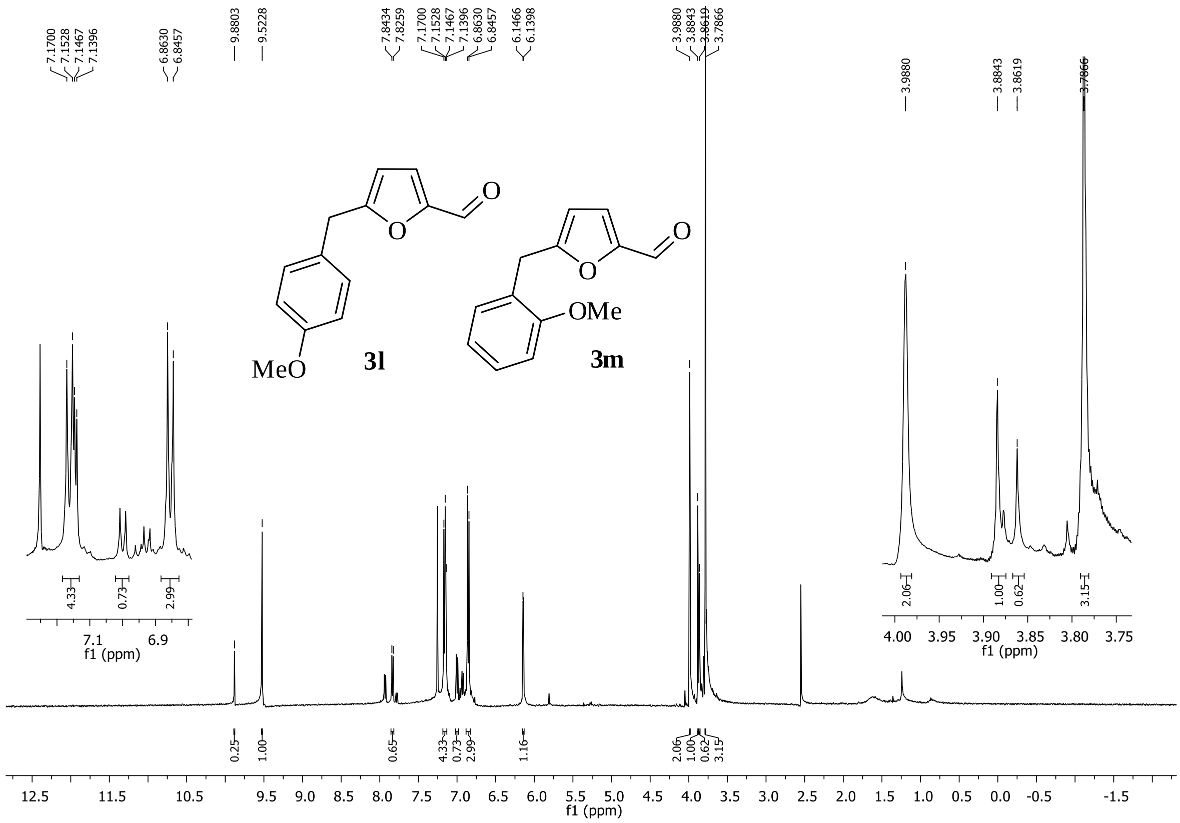

Figure S17. ^{1}H NMR spectrum of mixture of compounds **3l** and **3m** (500 MHz, $CDCl_3$).

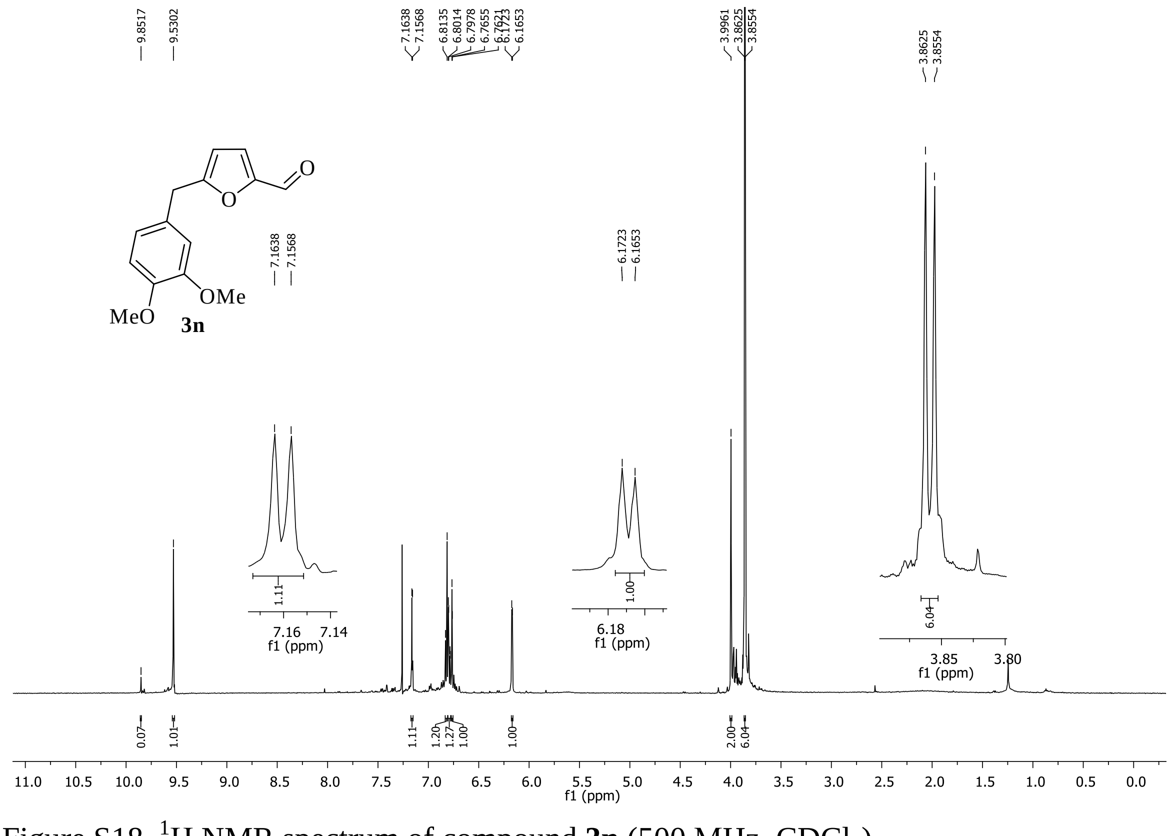

Figure S18. ^{1}H NMR spectrum of compound **3n** (500 MHz, $CDCl_3$).

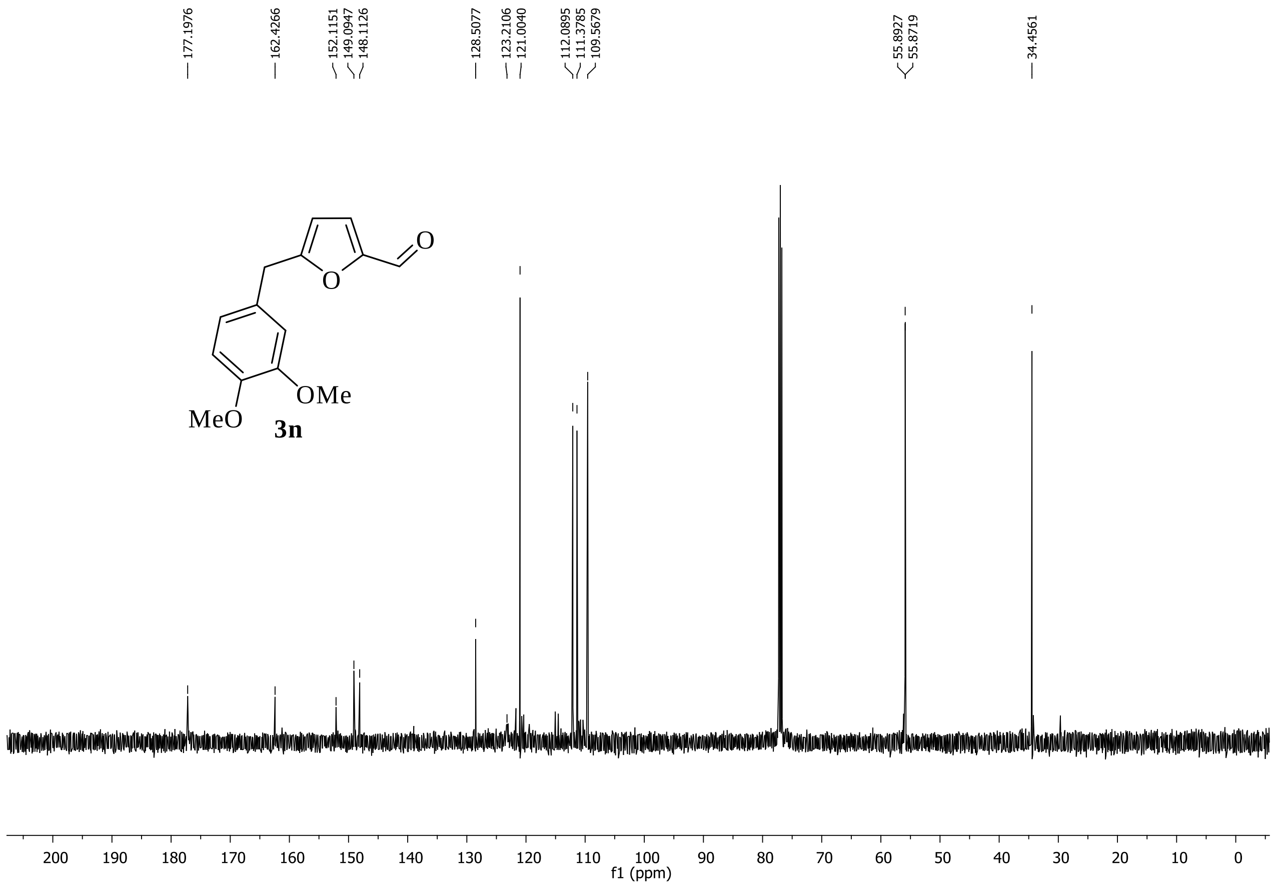

Figure S19. ^{13}C NMR spectrum of compound **3n** (125 MHz, $CDCl_3$).

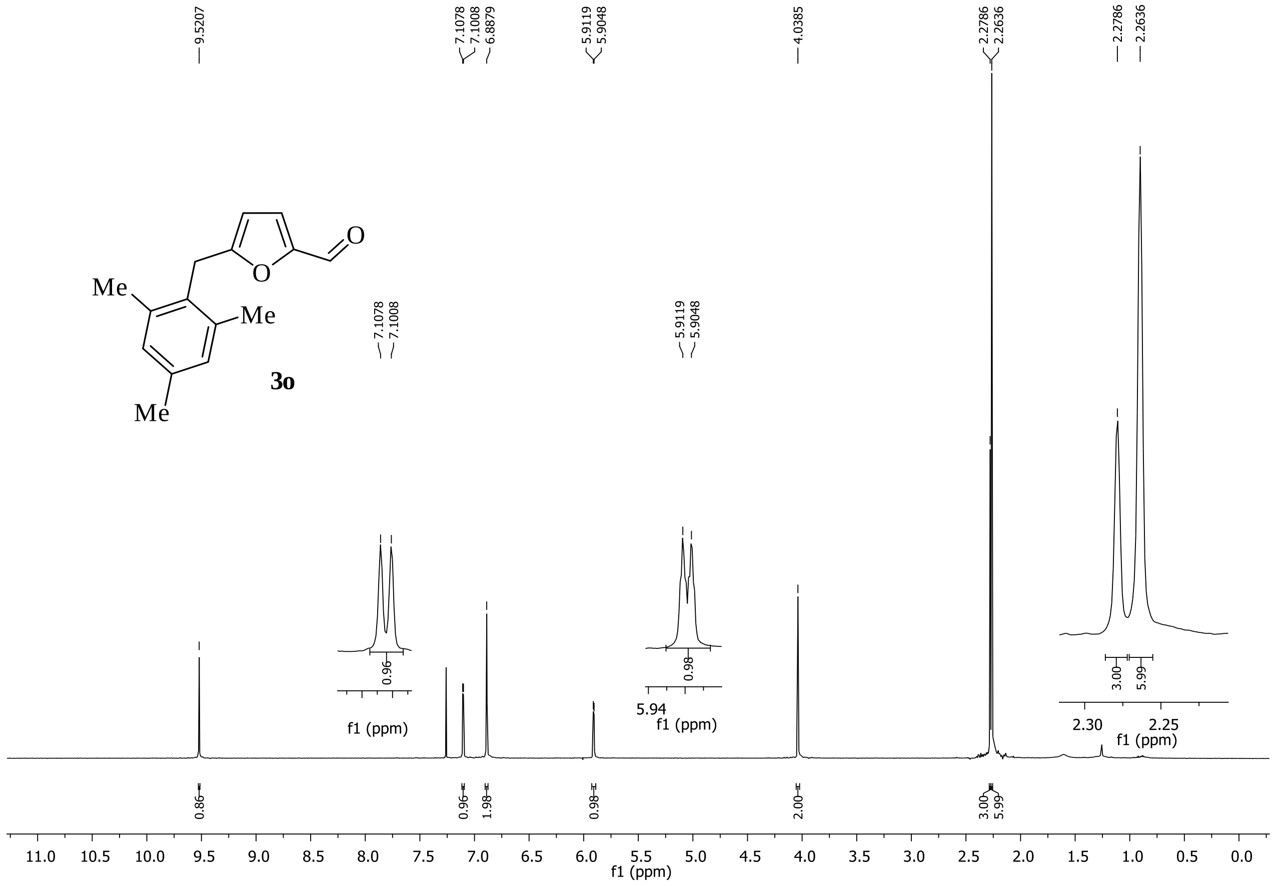

Figure S20. ^{1}H NMR spectrum of compound **3o** (500 MHz, $CDCl_3$).

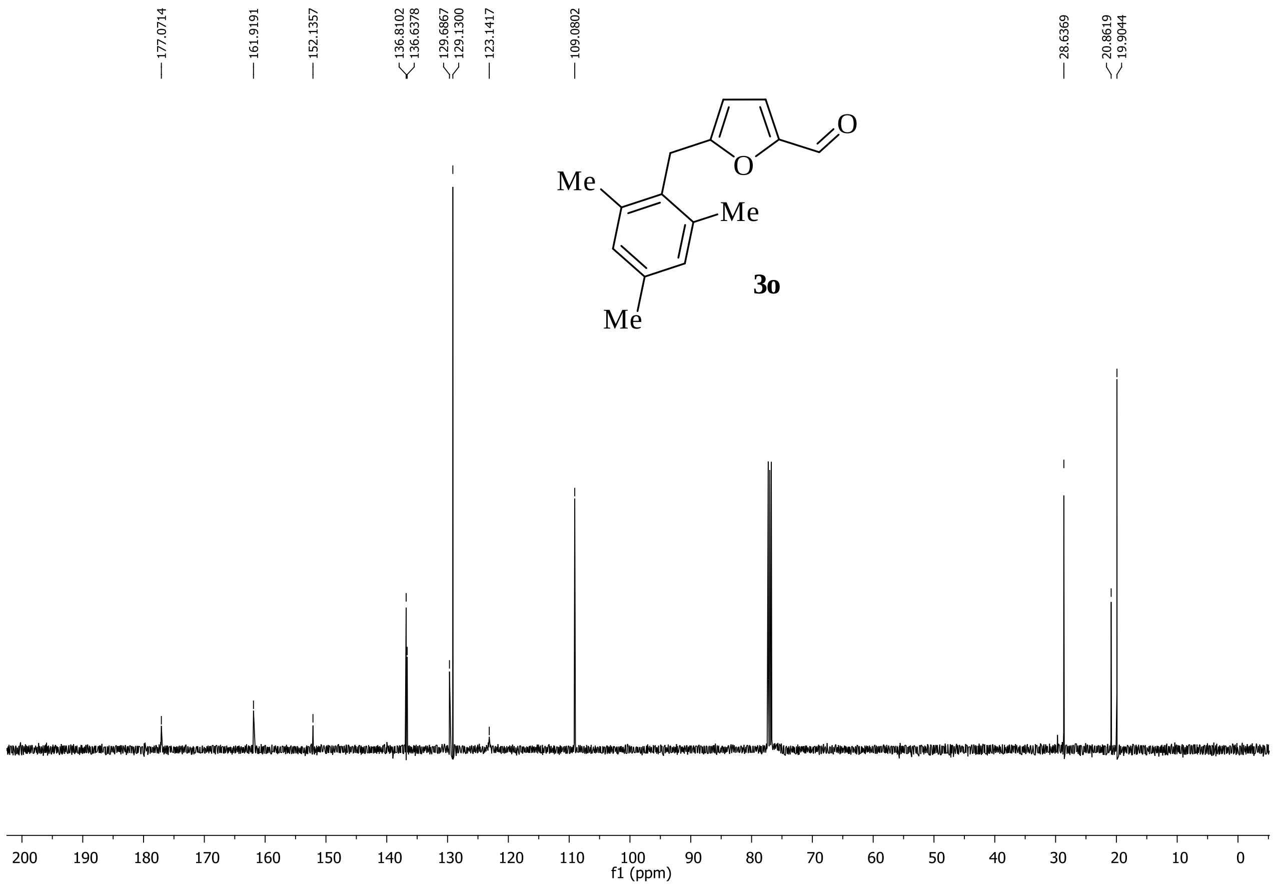

Figure S21. ^{13}C NMR spectrum of compounds **3o** (125 MHz, $CDCl_3$).

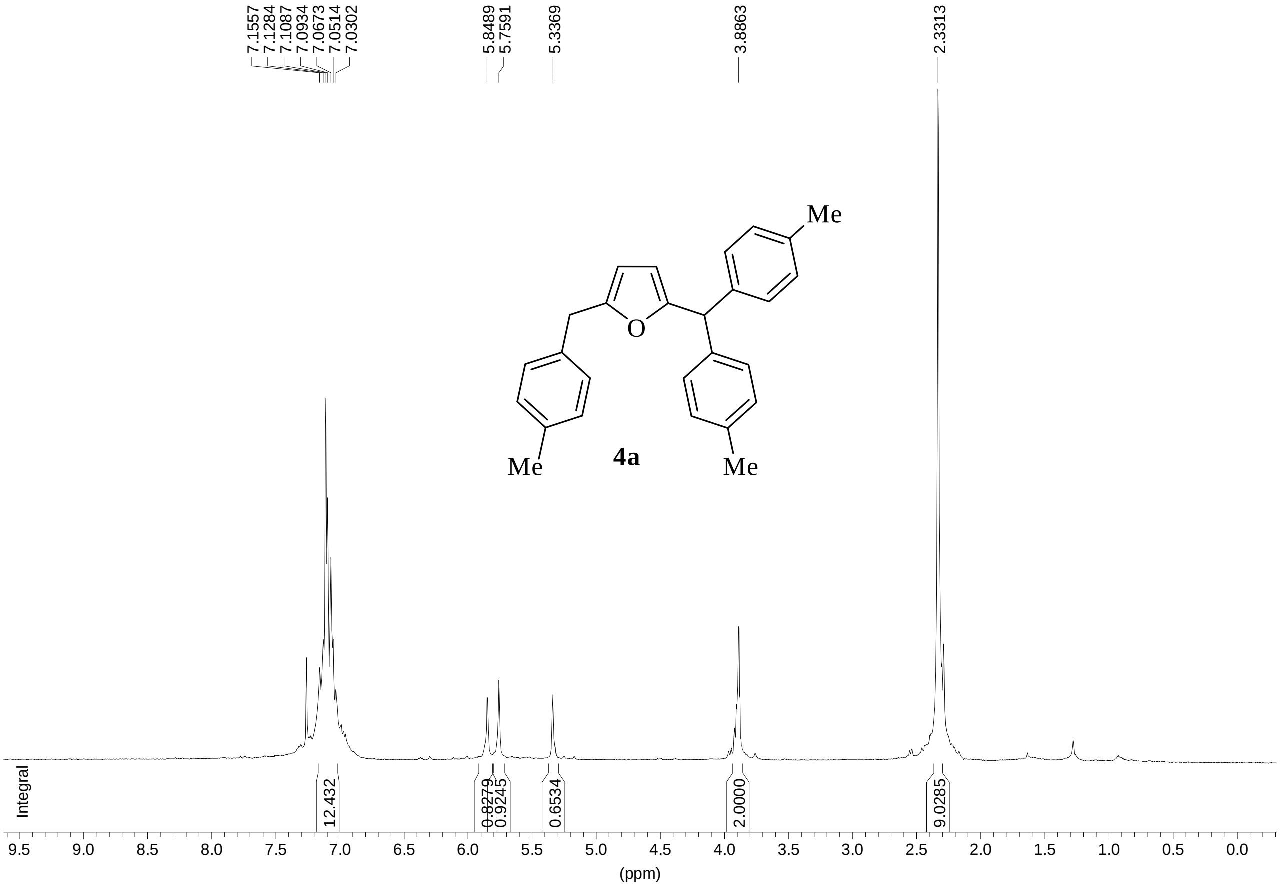

Figure S22. ^{1}H NMR spectrum of compound **4a** (500 MHz, $CDCl_3$).

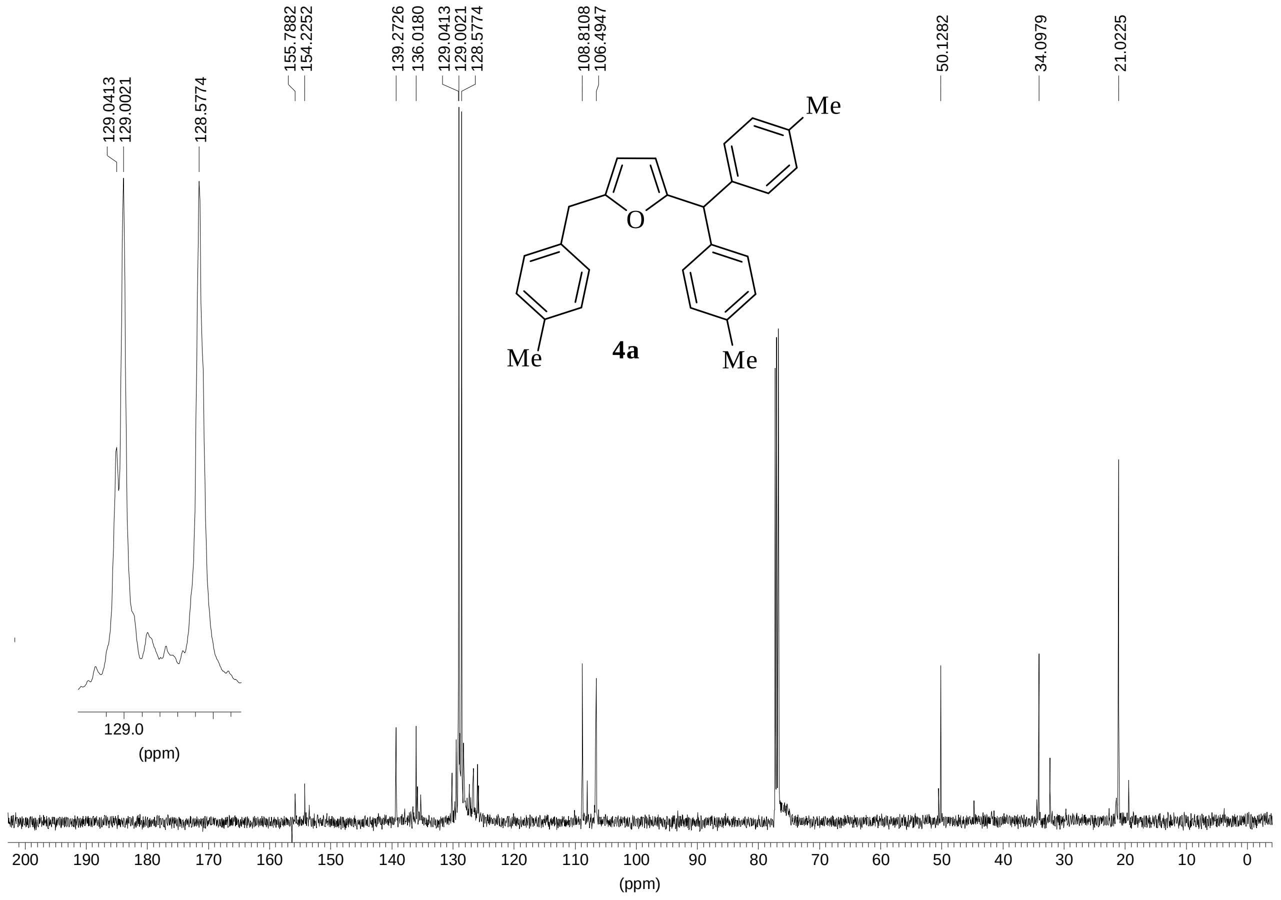

Figure S23. ^{13}C NMR spectrum of compound **4a** (125 MHz, $CDCl_3$).

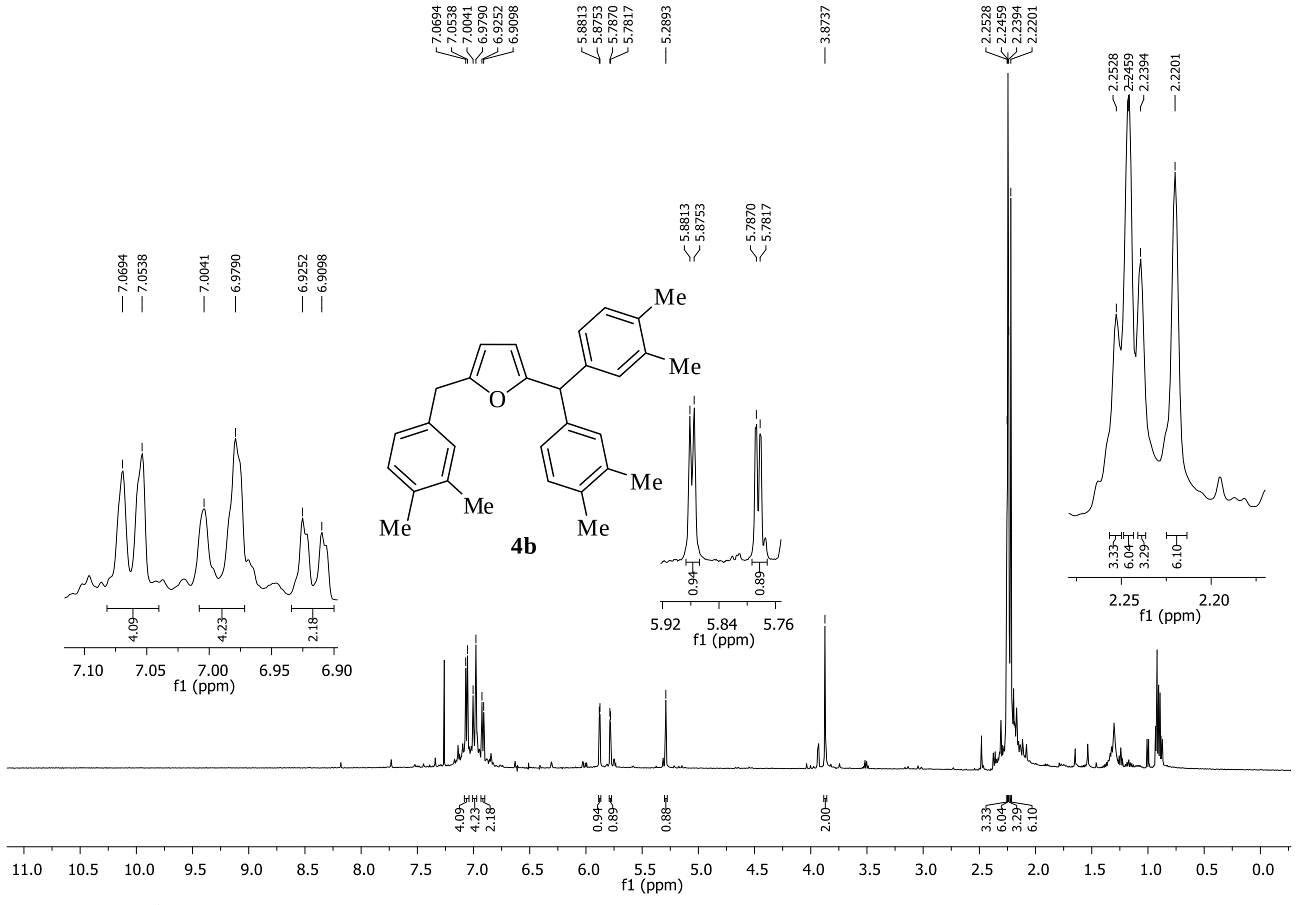

Figure S24. ^{1}H NMR spectrum of compound **4b** (500 MHz, $CDCl_3$).

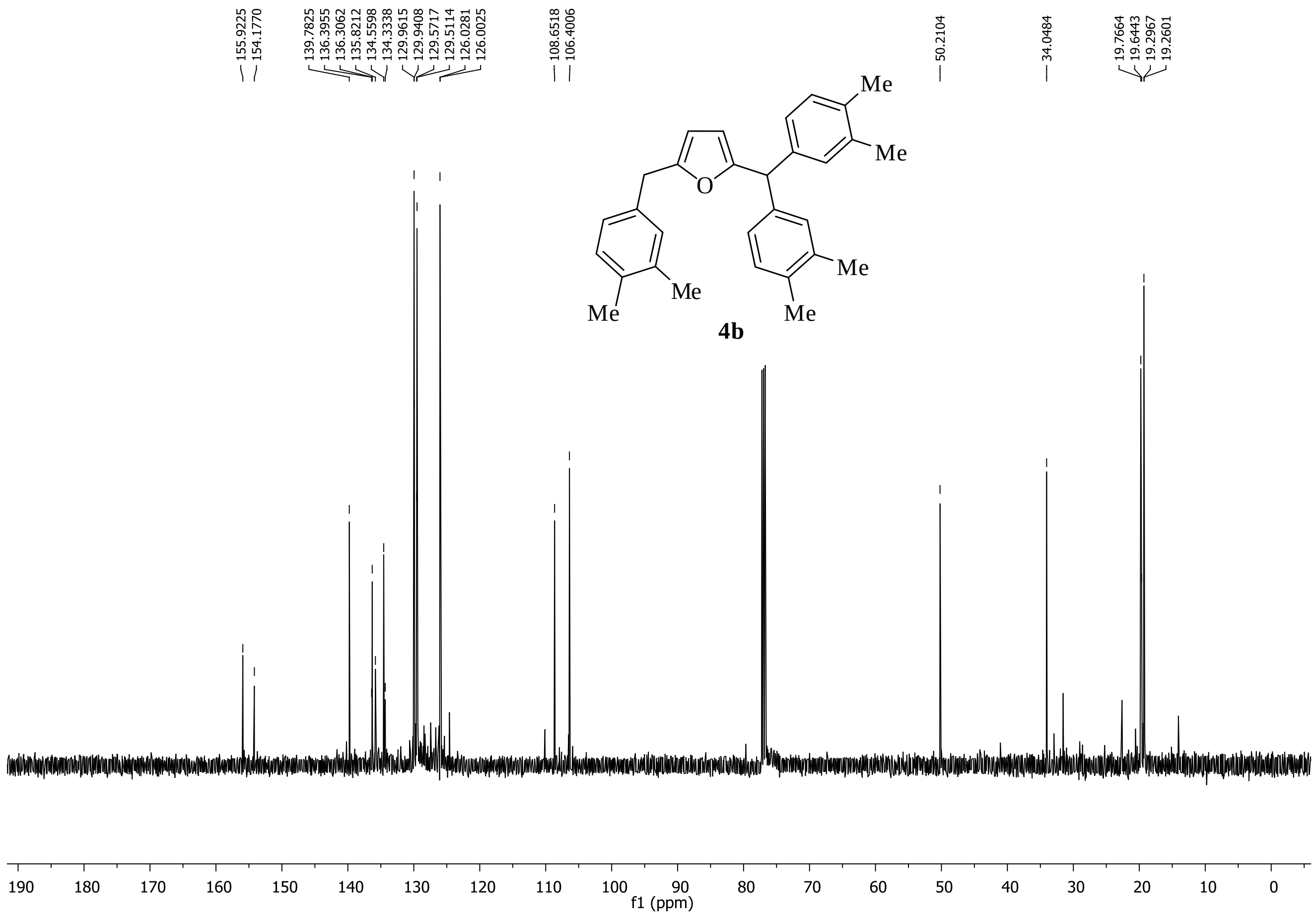

Figure S25. ^{13}C NHM spectrum of compound **4b** (125 MHz, $CDCl_3$).

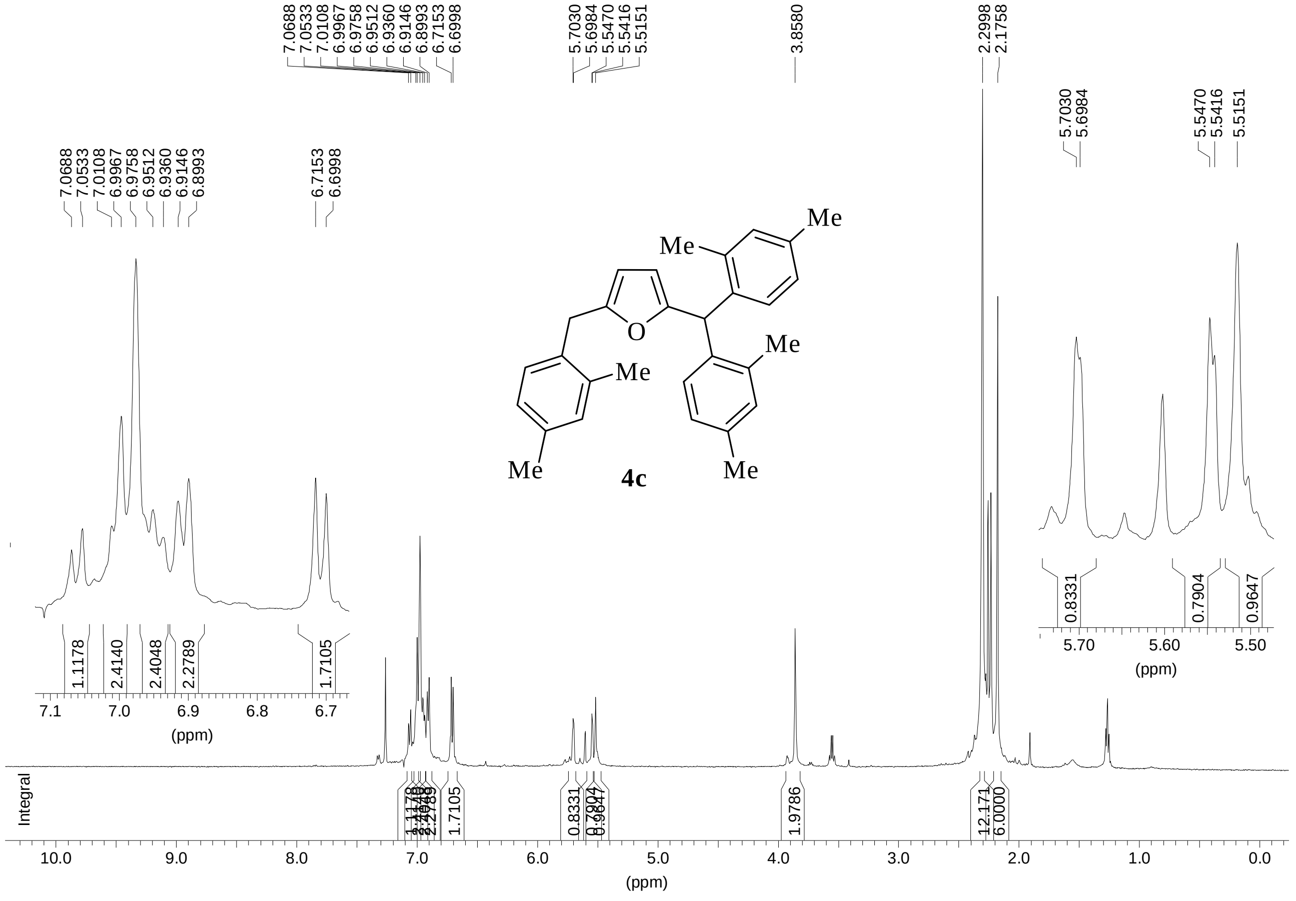

Figure S26. ^{1}H NMR spectrum of compound **4c** (500 MHz, $CDCl_3$).

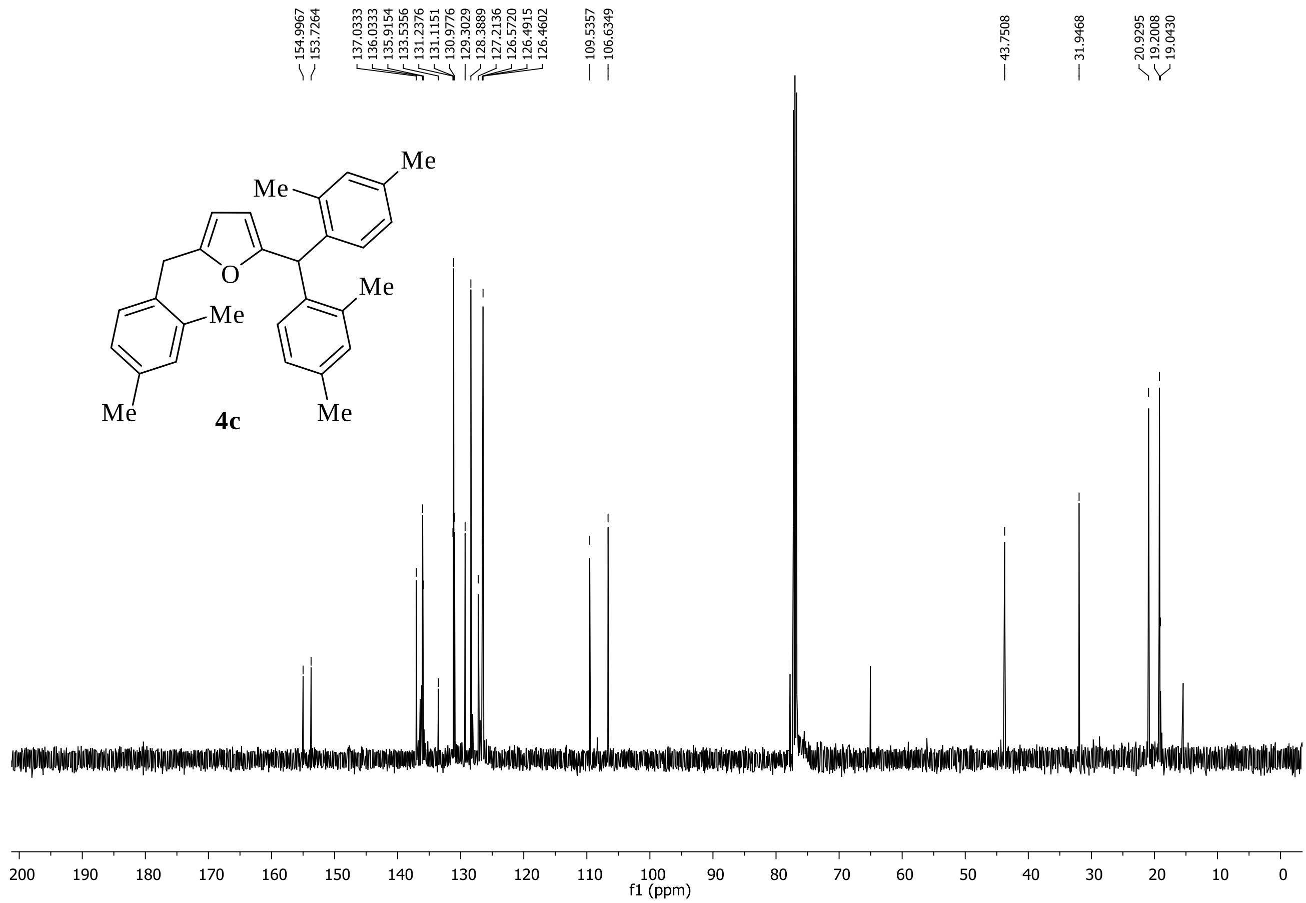

Figure S27. ^{13}C NMR spectrum of compound **4c** (125 MHz, $CDCl_3$).

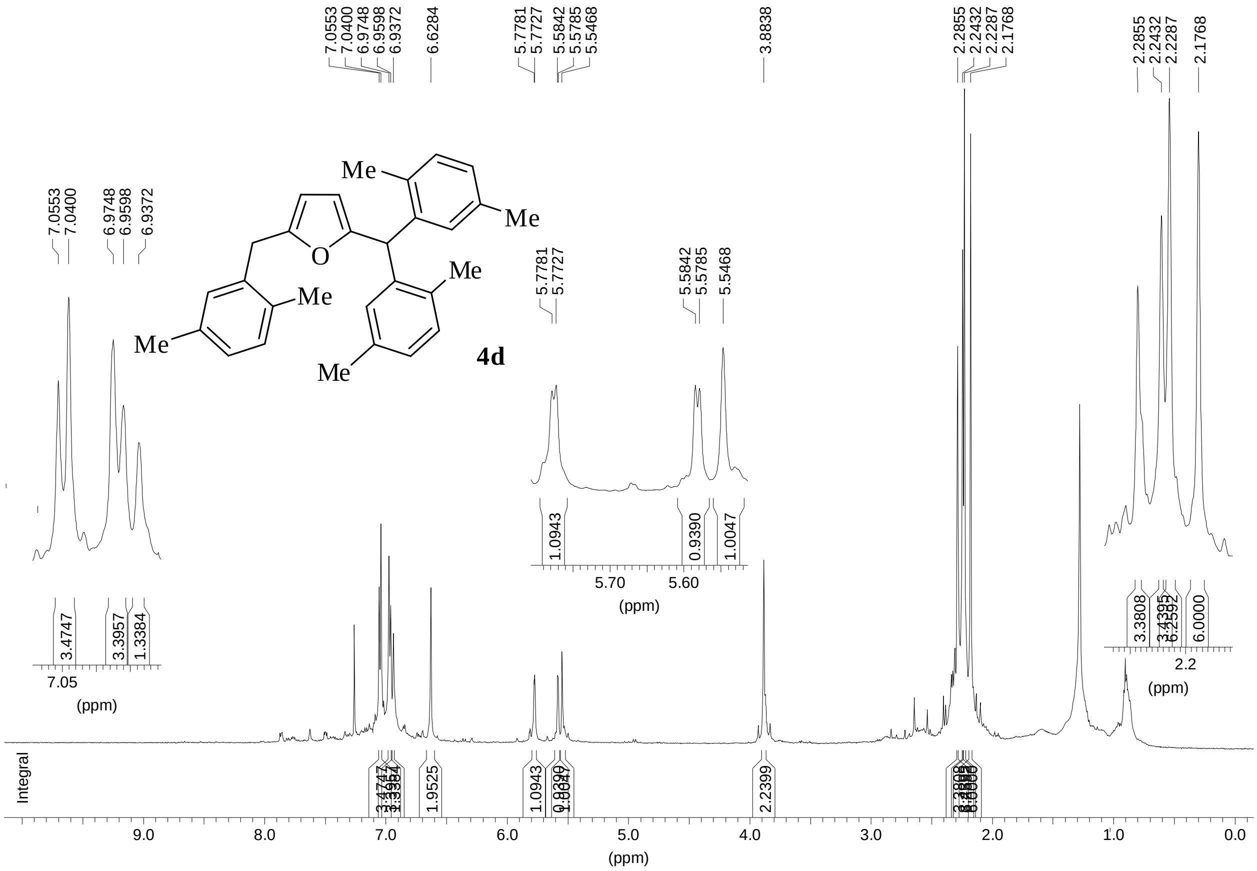

Figure S28. ^{1}H NMR spectrum of compound **4d** (500 MHz, $CDCl_3$).

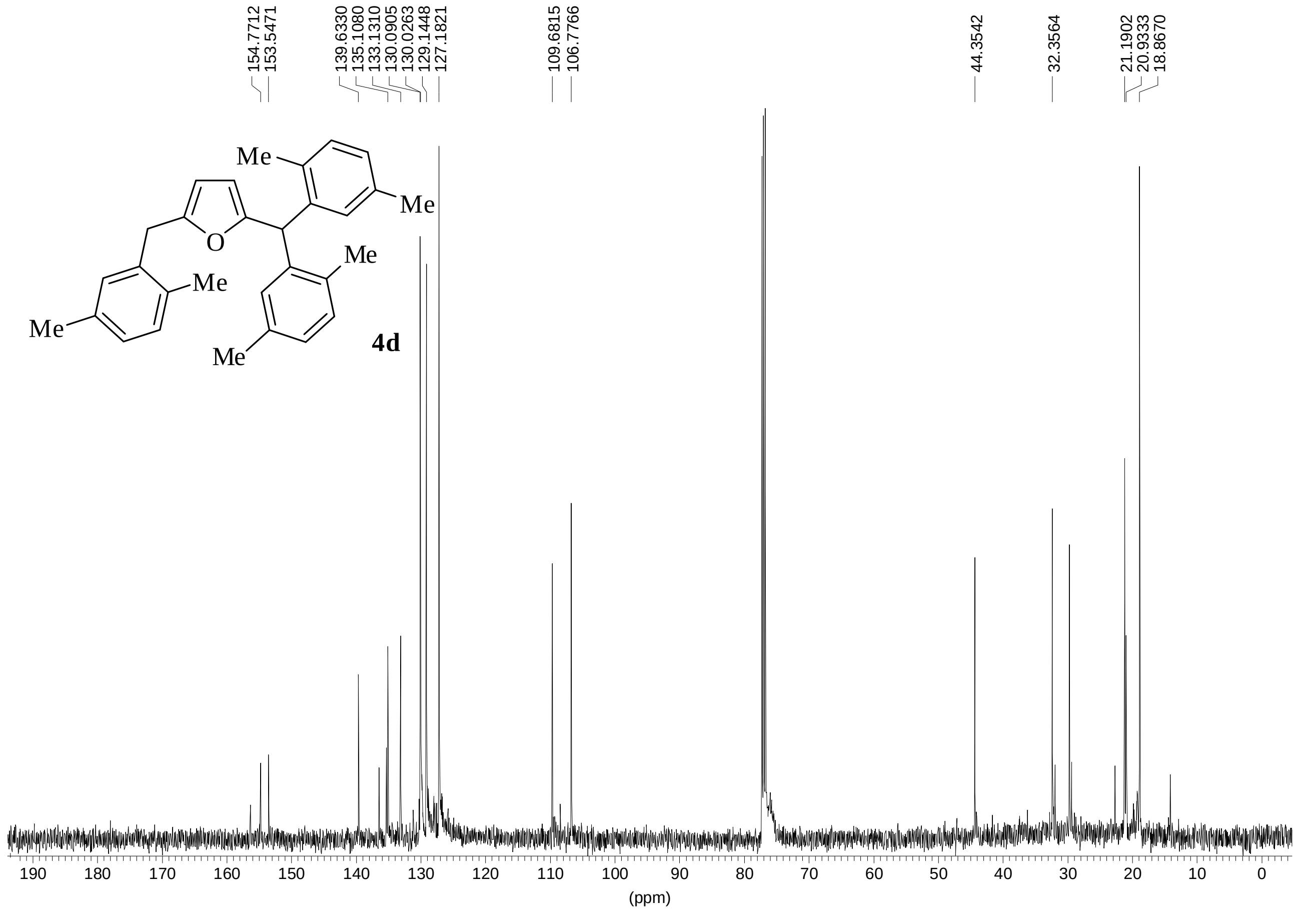

Figure S29. ^{13}C NHM spectrum of compound **4d** (125 MHz, $CDCl_3$).

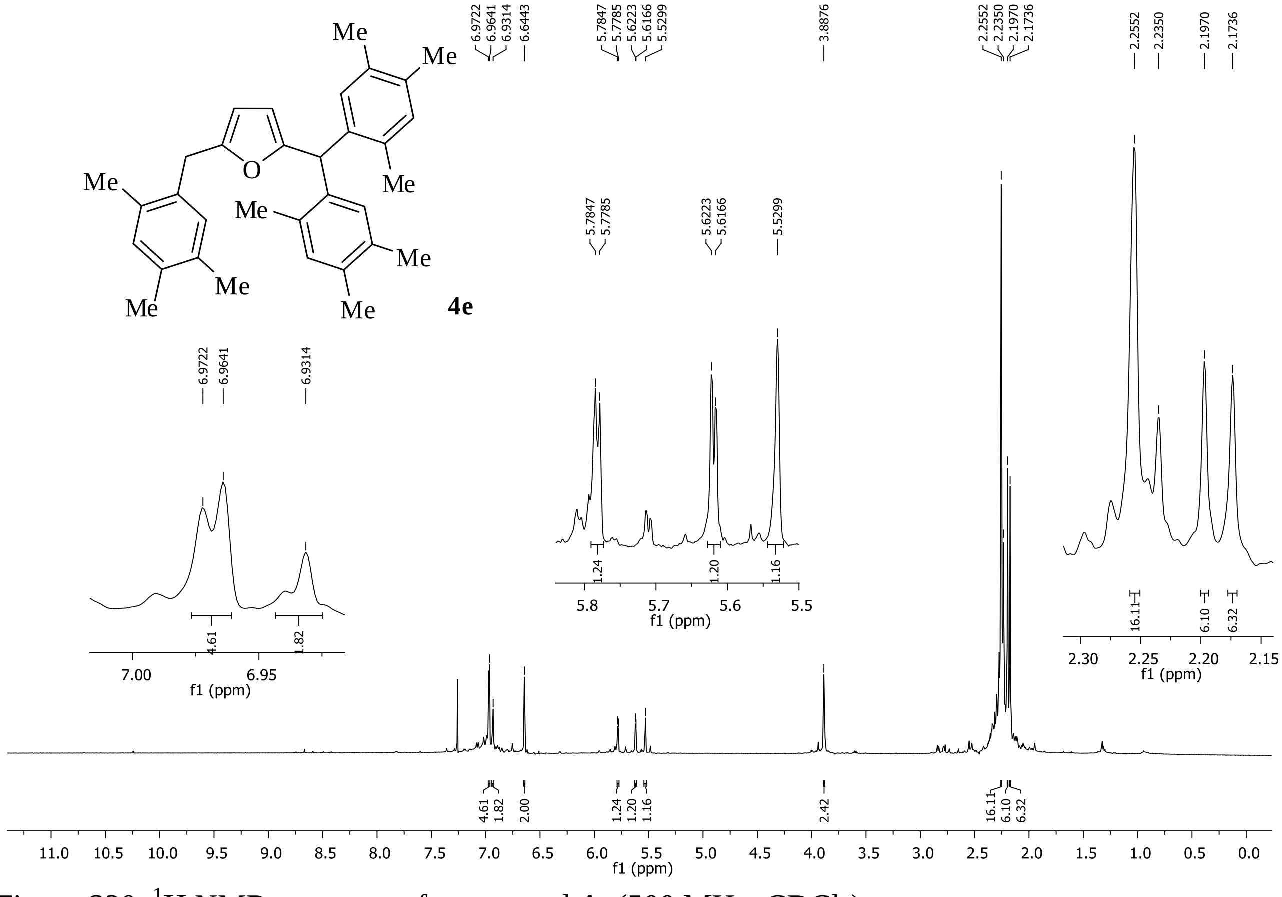

Figure S30. ^{1}H NMR spectrum of compound **4e** (500 MHz, $CDCl_3$).

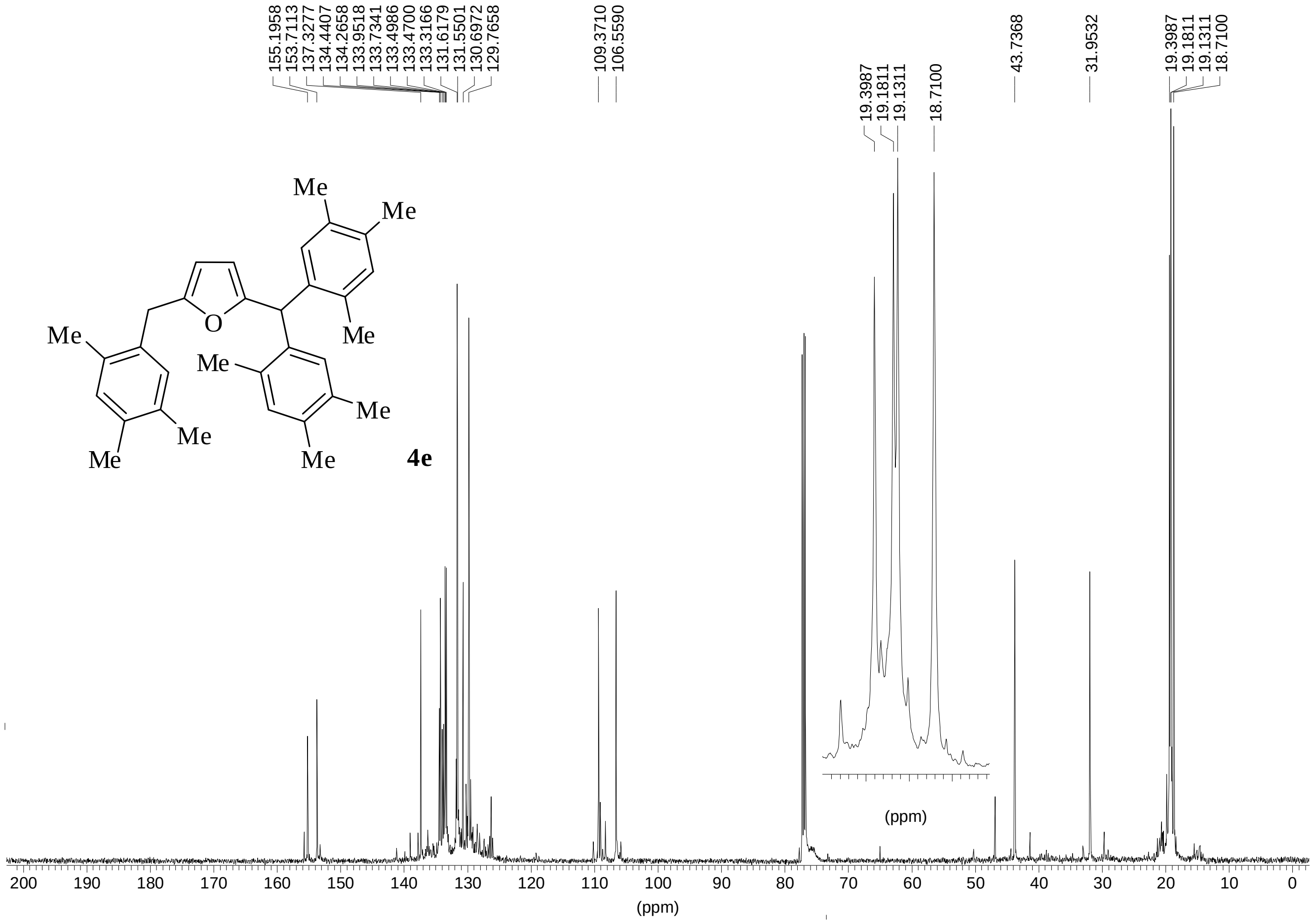

Figure S31. ^13^C NMR spectrum of compound **4e** (125 MHz, $CDCl_3$).

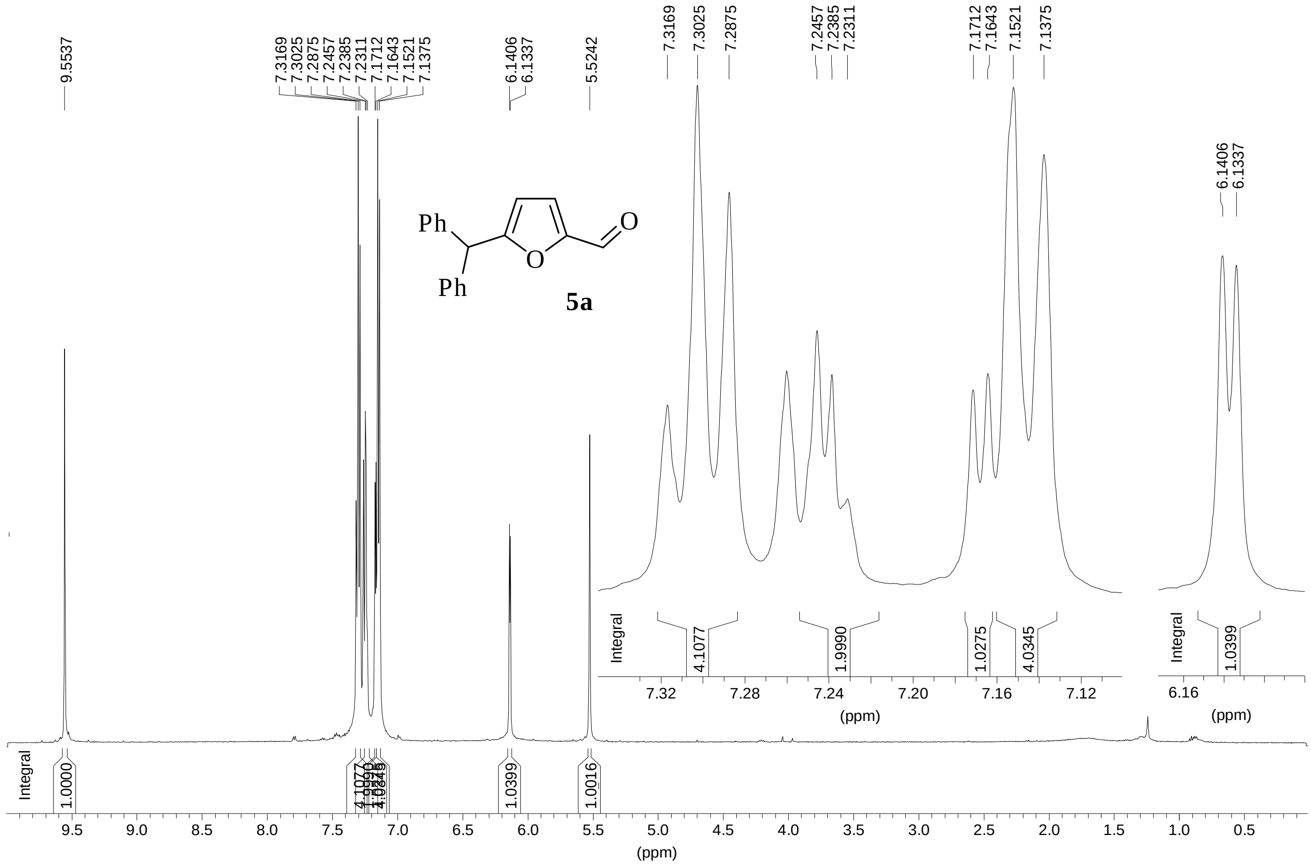

Figure S32. ^1^H NMR spectrum of compound **5a** (500 MHz, $CDCl_3$).

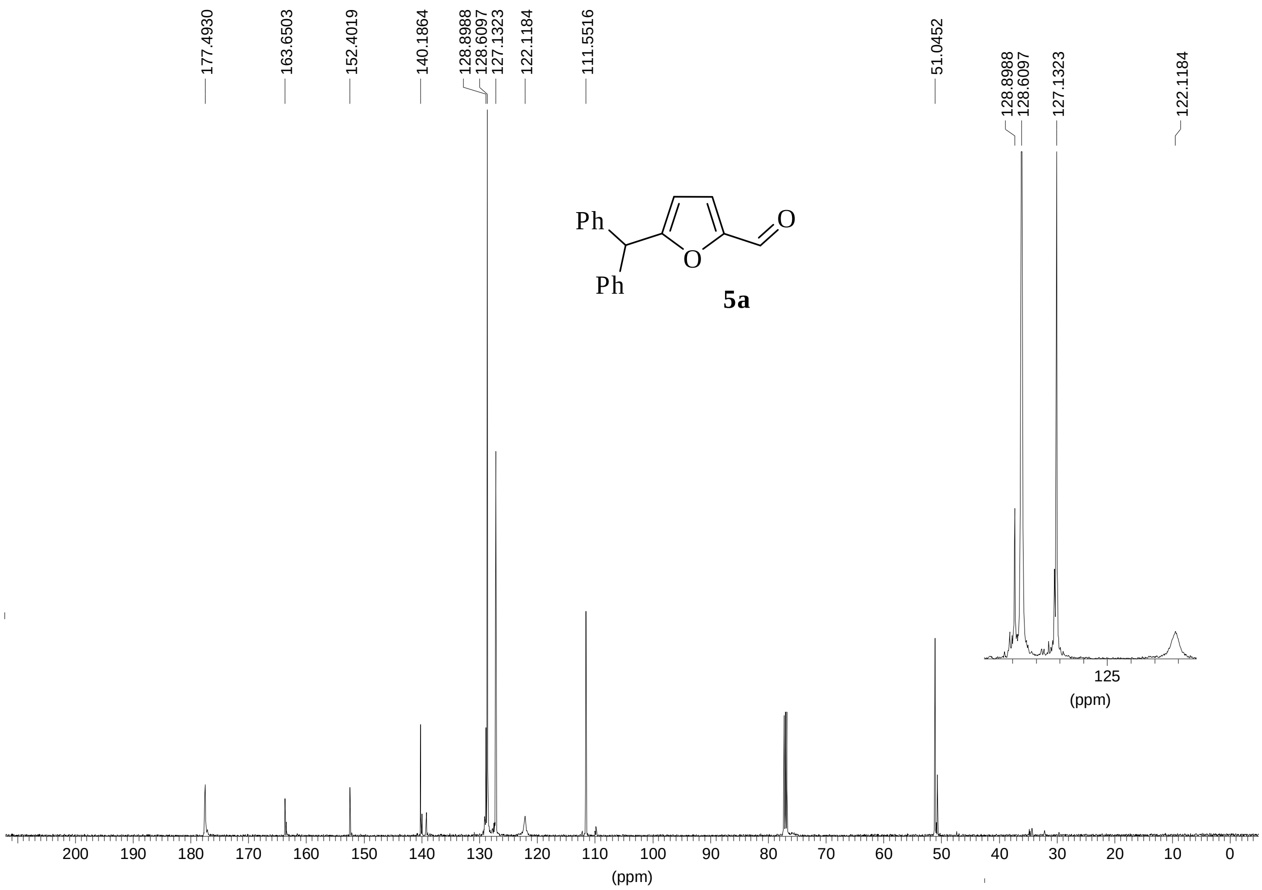

Figure S33. ^{13}C NMR spectrum of compound **5a** (125 MHz, $CDCl_3$).

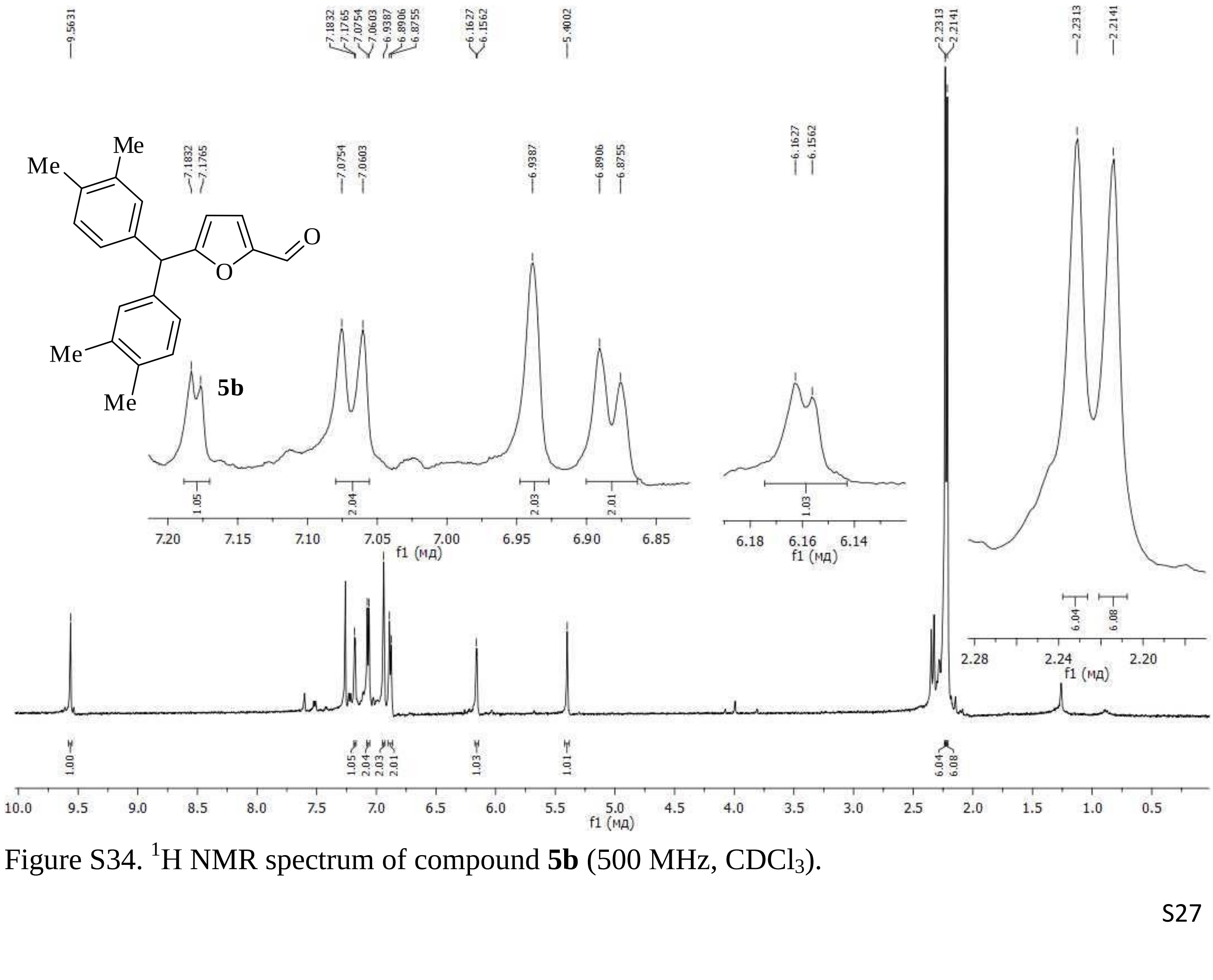

Figure S34. ^{1}H NMR spectrum of compound **5b** (500 MHz, $CDCl_3$).

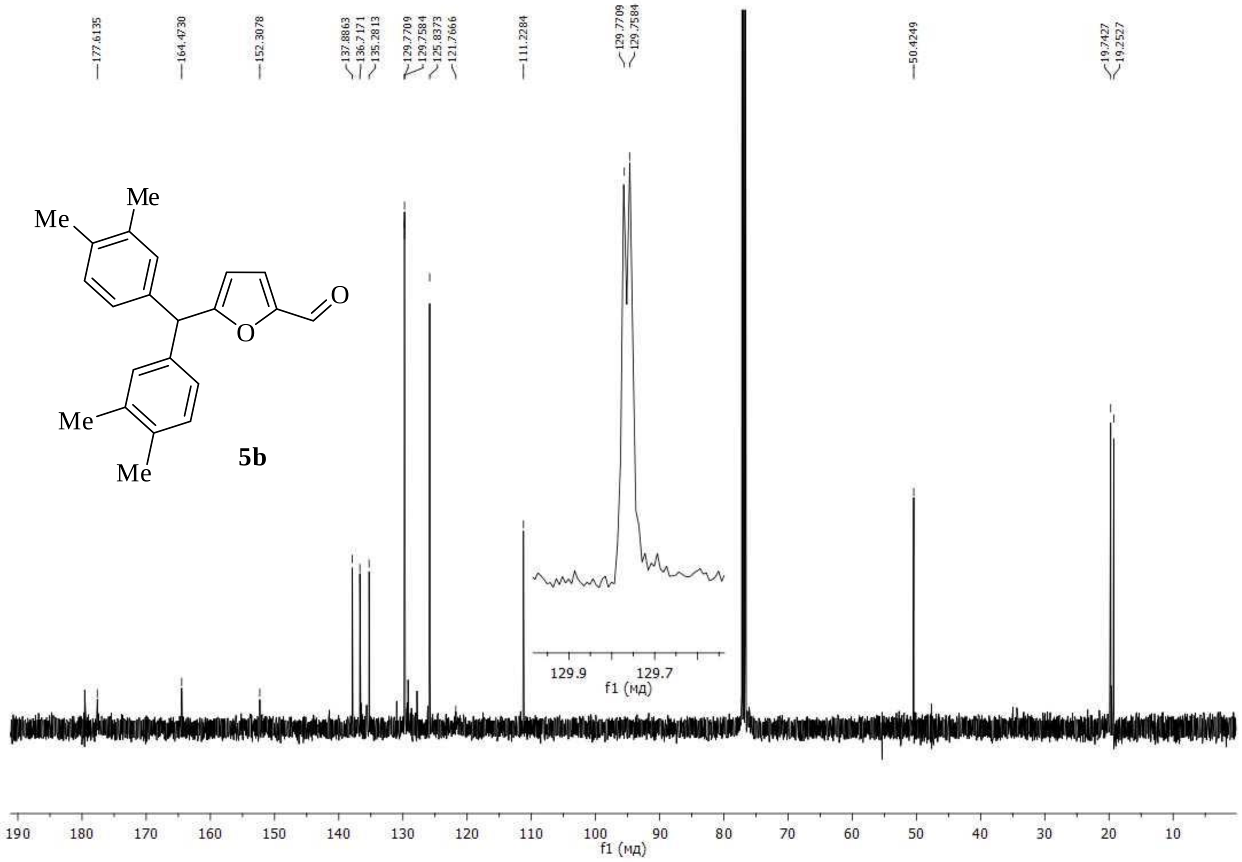

Figure S35. ^{13}C NMR spectrum of compound **5b** (125 MHz, $CDCl_3$).

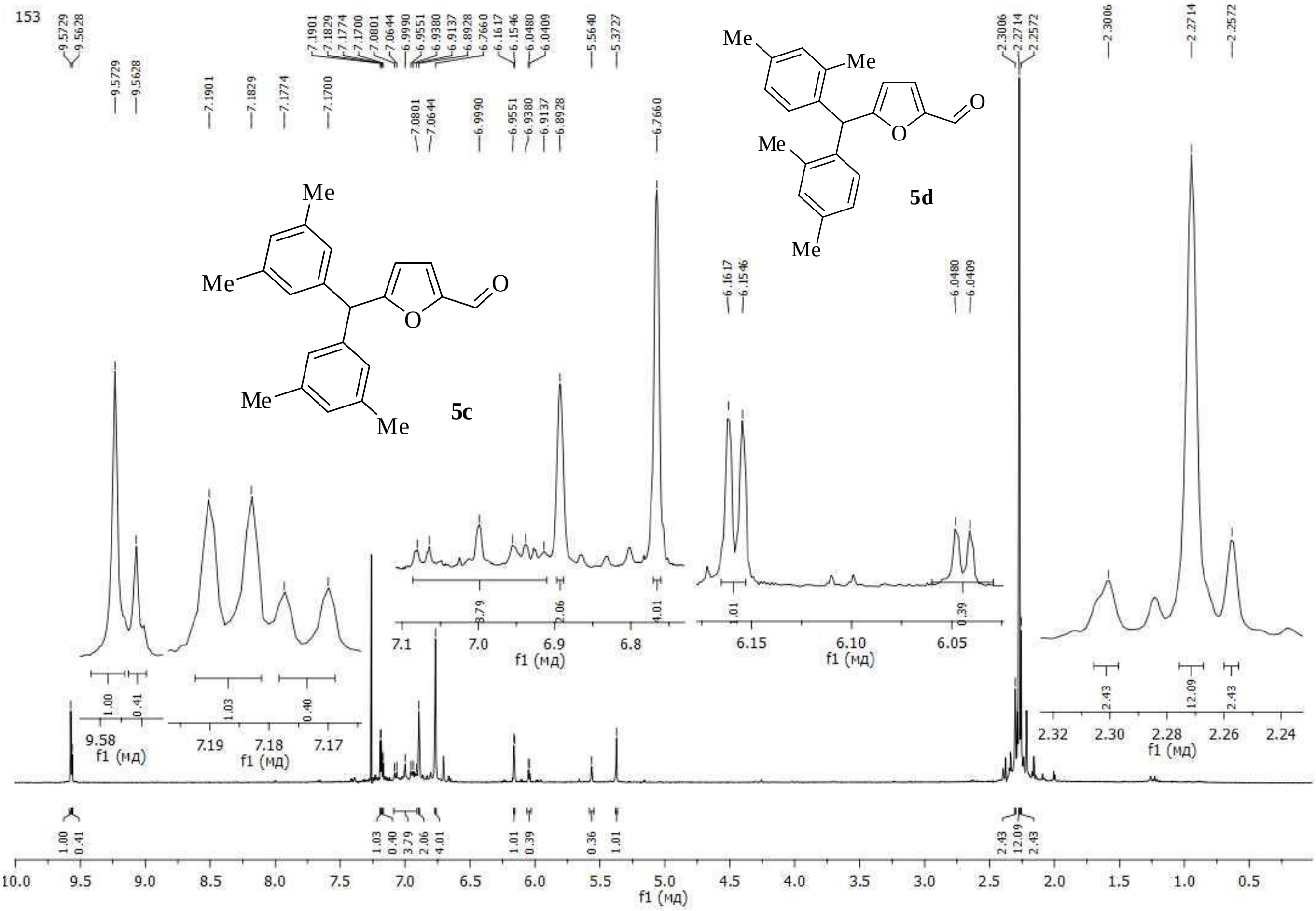

Figure S36. ^{1}H NMR spectrum of mixture of compounds **5c** and **5d** (500 MHz, $CDCl_3$).

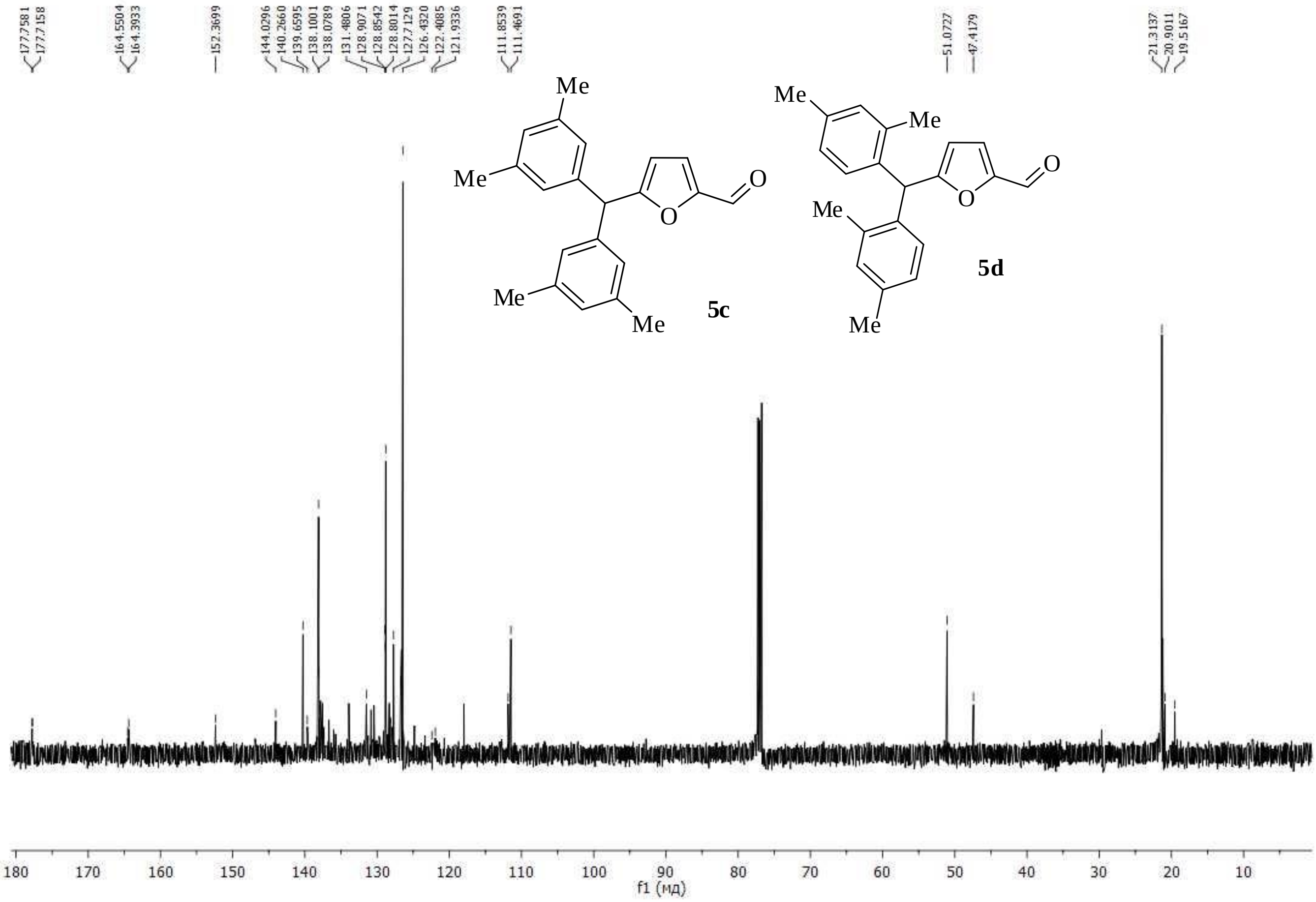

Figure S37. ^{13}C NMR spectrum of mixture of compounds **5c** and **5d** (125 MHz, $CDCl_3$).

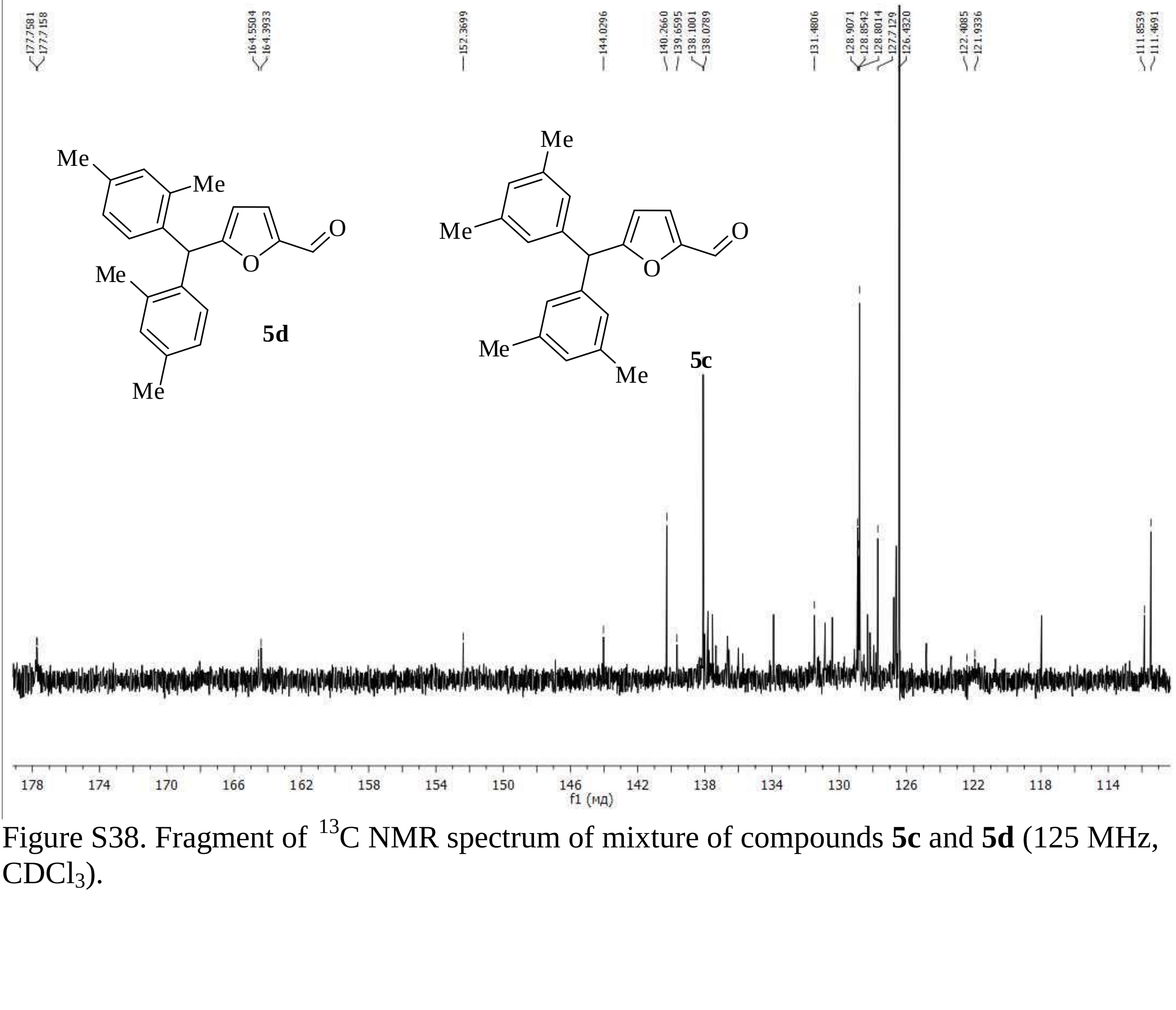

Figure S38. Fragment of ^{13}C NMR spectrum of mixture of compounds **5c** and **5d** (125 MHz, $CDCl_3$).

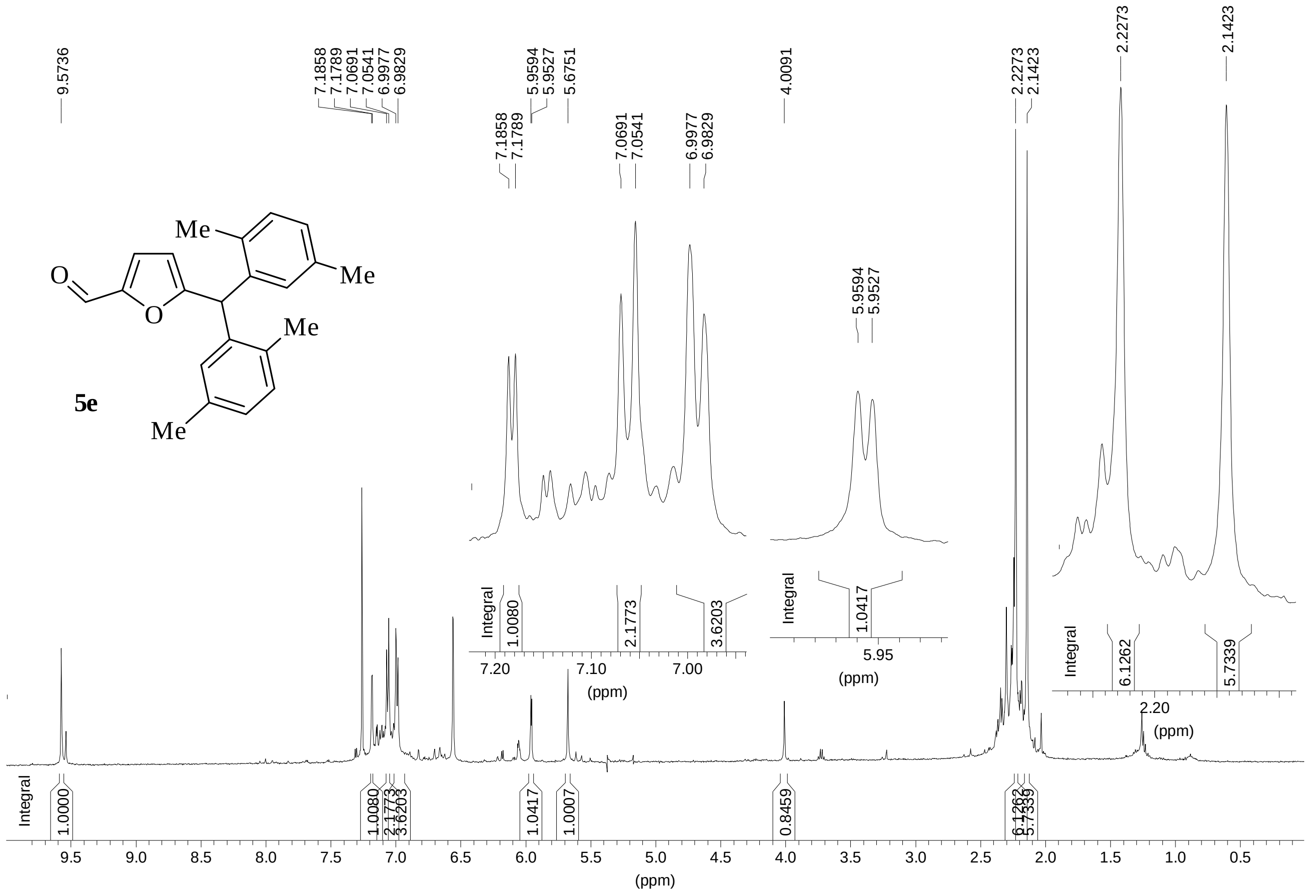

Figure S39. ^{1}H NMR spectrum of compound **5e** (500 MHz, $CDCl_3$).

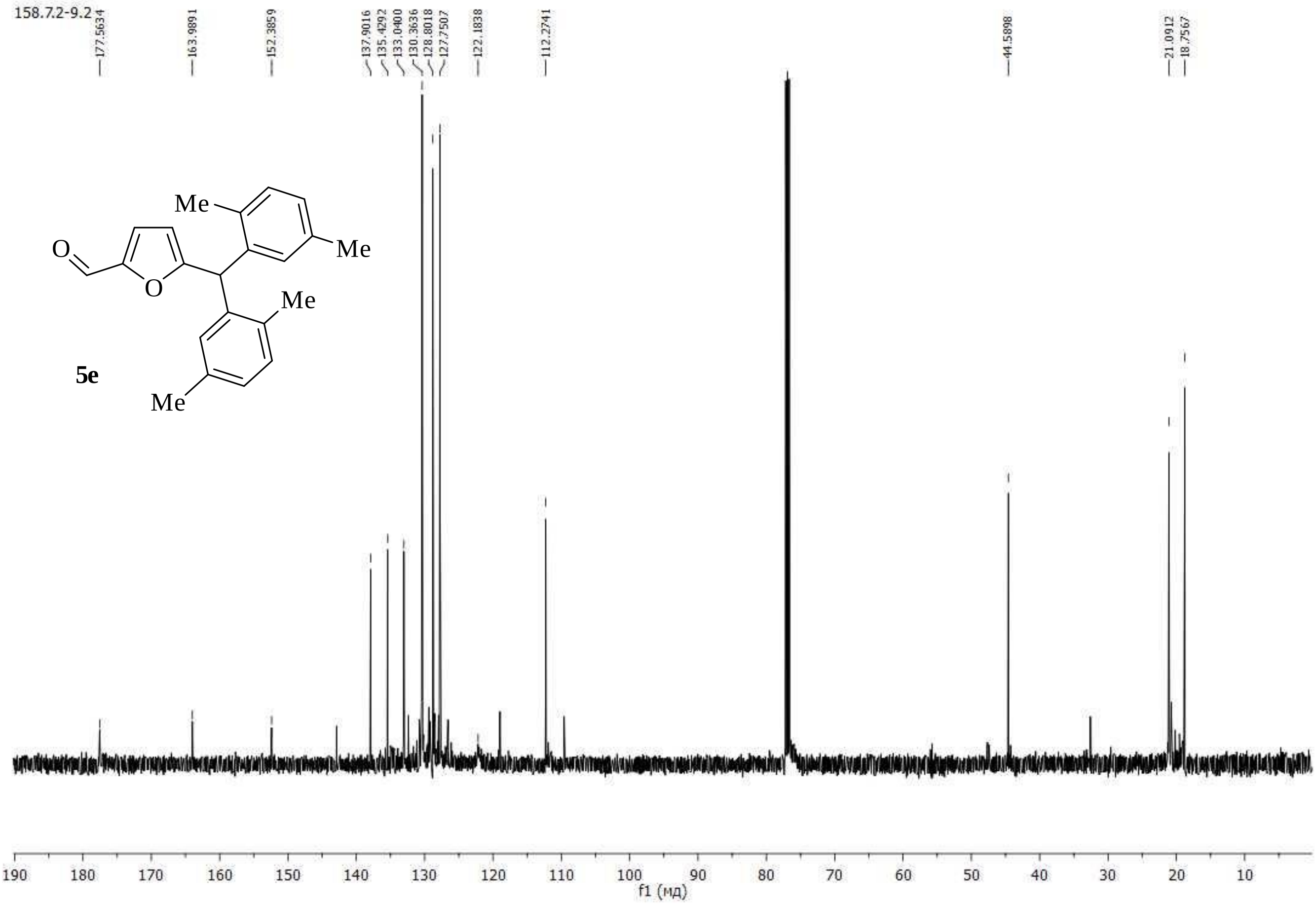

Figure S40. ^{13}C NMR spectrum of compound **5e** (125 MHz, $CDCl_3$).

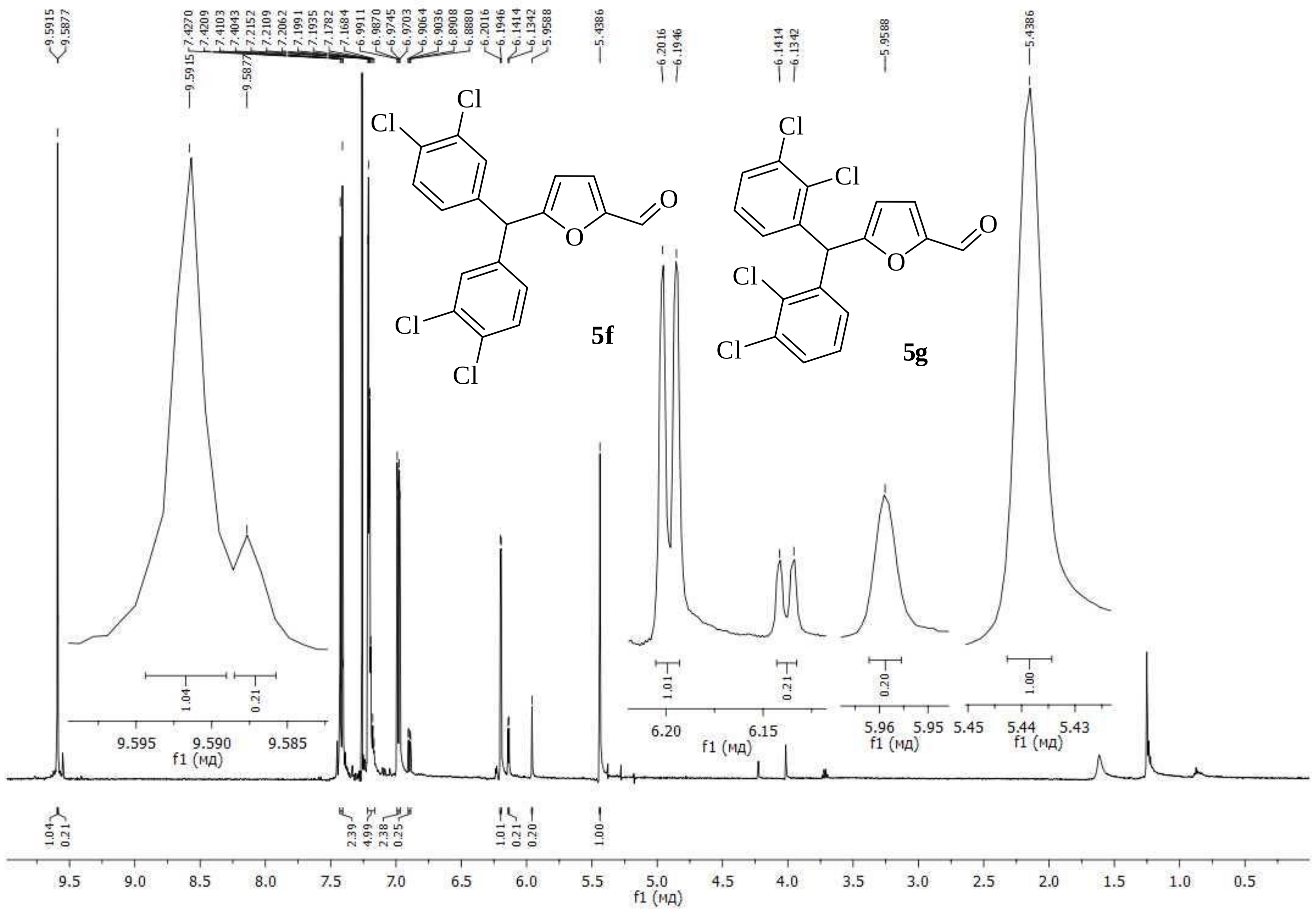

Figure S41. ^{1}H NMR spectrum of mixture of compounds **5f** and **5g** (500 MHz, $CDCl_3$).

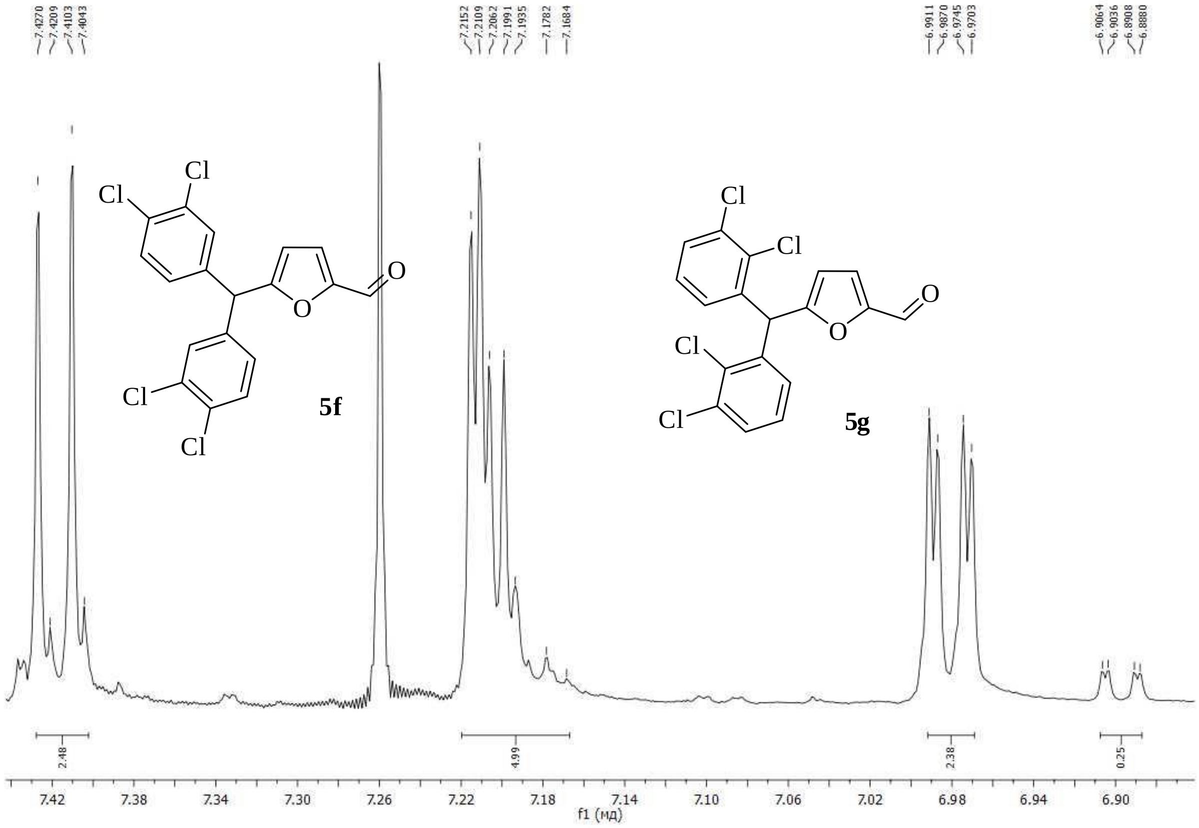

Figure S42. Fragment of ^{1}H NMR spectrum of mixture of compounds **5f** and **5g** (500 MHz, $CDCl_3$).

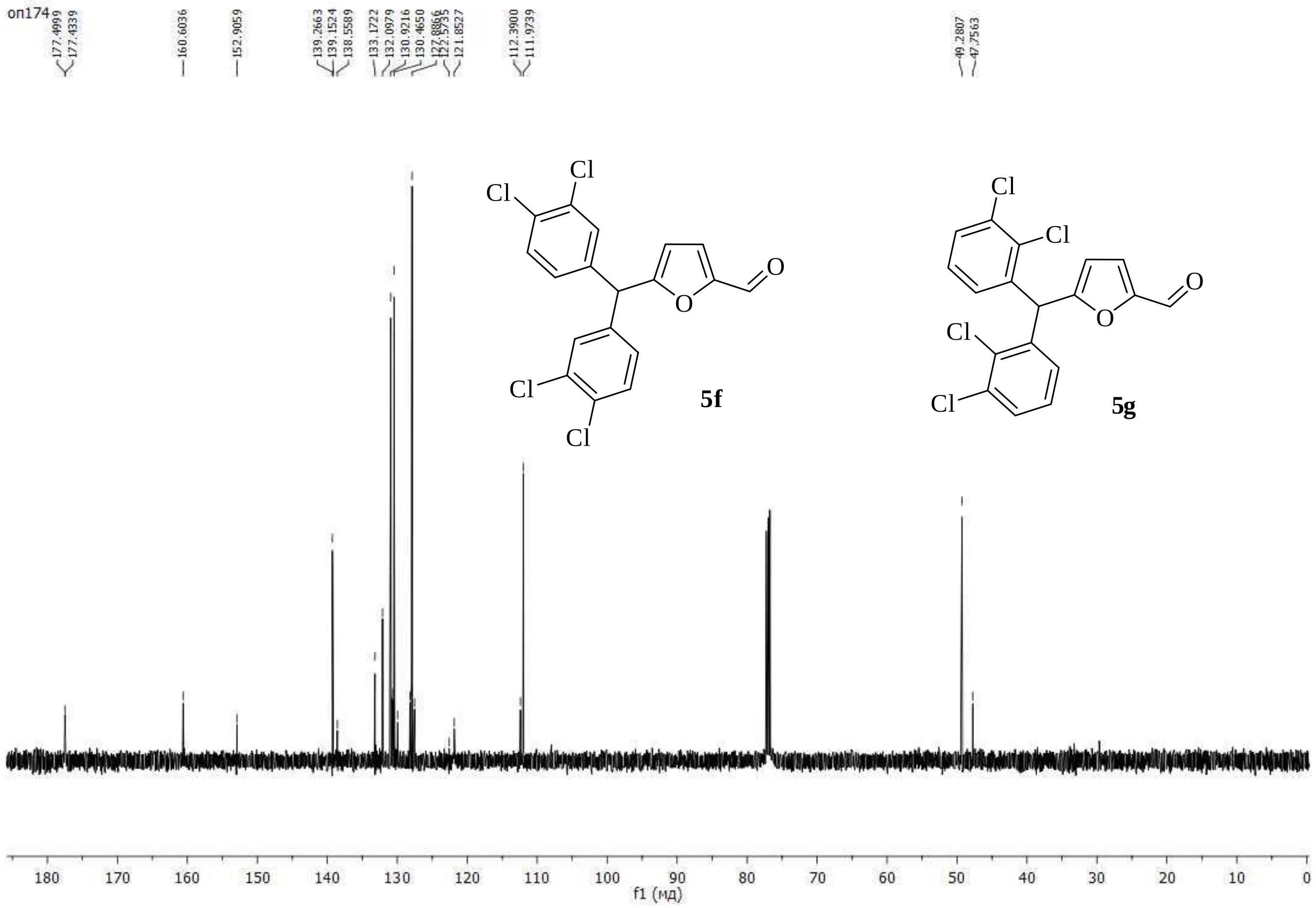

Figure S43. ^{13}C NMR spectrum of mixture of compounds **5f** and **5g** (125 MHz, $CDCl_3$).

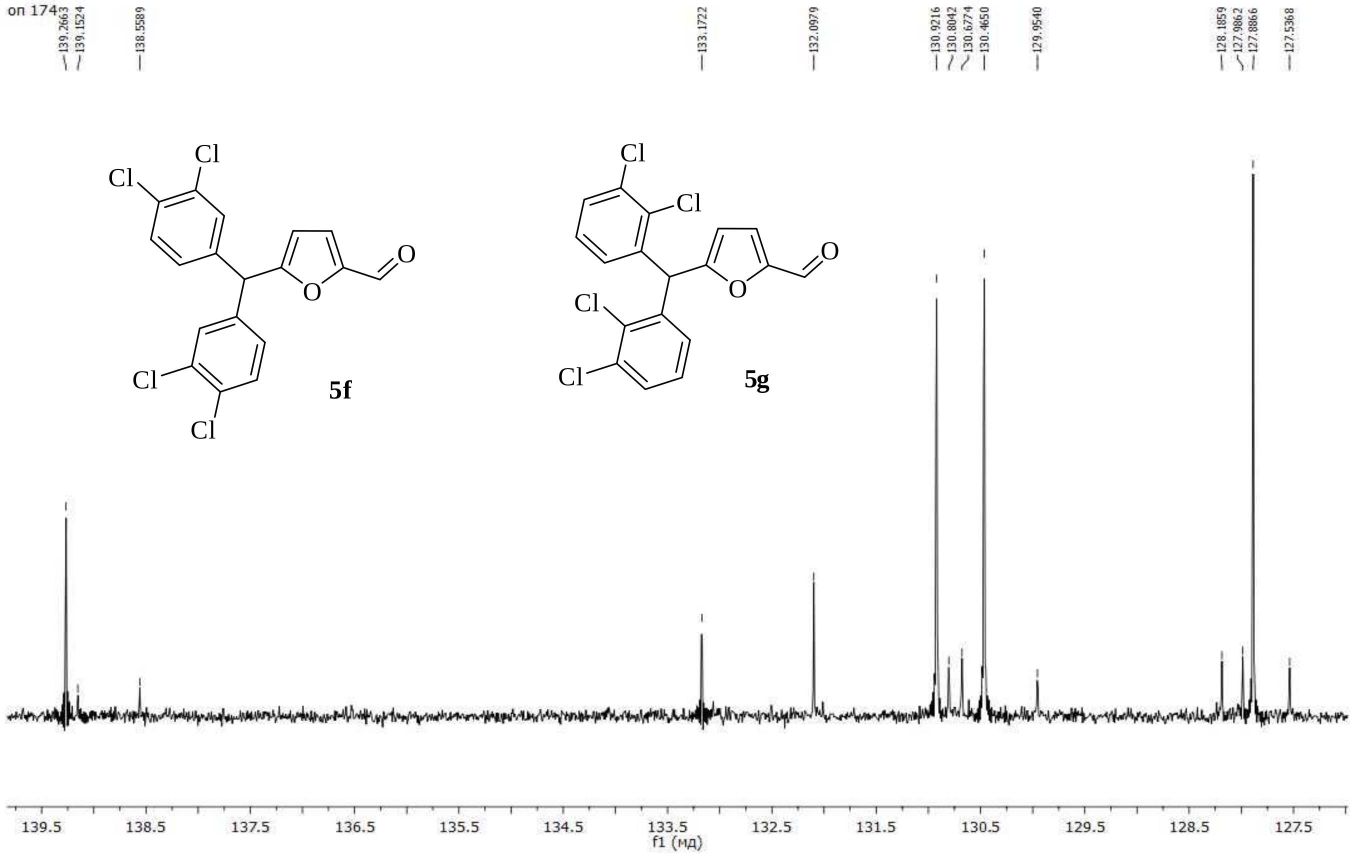

Figure S44. Fragment of ^{13}C NMR spectrum of mixture of compounds **5f** and **5g** (125 MHz, $CDCl_3$).

^{1}H and ^{13}C NMR Spectra of cations A, D in TfOH and their comparison with the spectra of neutral initial compounds 1a and 2

^{1}H NMR spectrum of cation **A** (CF_3SO_3H, 400 MHz, r.t.).

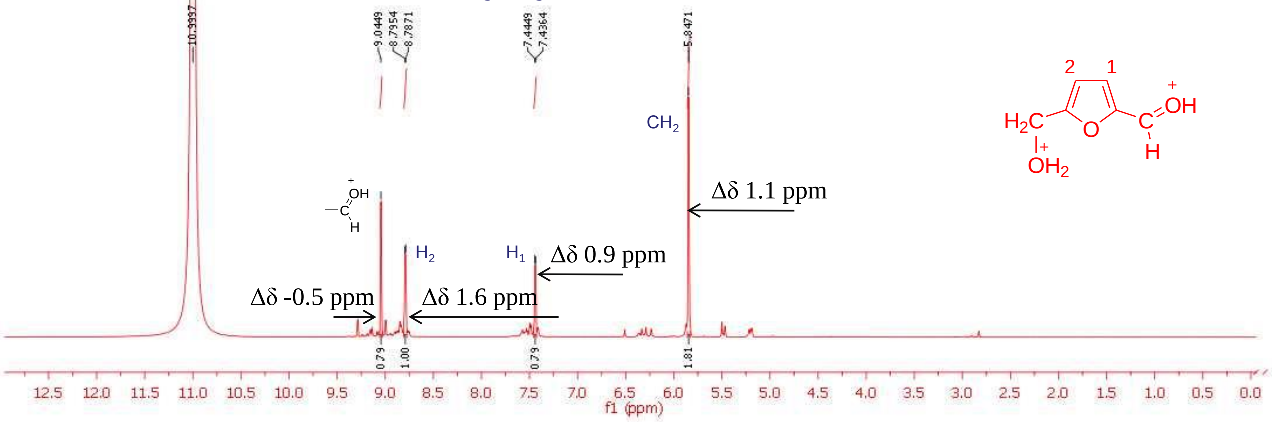

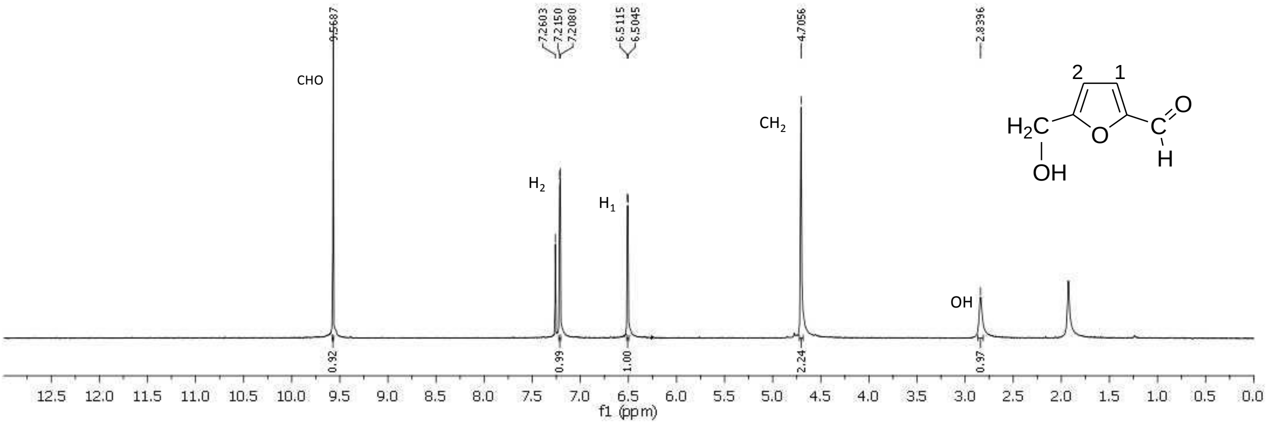

^{1}H NMR spectrum of compound 1 ($CDCl_3$, 500 MHz, r.t.).

Figure S45. Comparison of ^{1}H NMR spectra of **A** and **1a** (arrows show shifts of the corresponding signals).

^{13}C NMR spectrum of cation A (CF_3SO_3H , 100 MHz, r.t.).

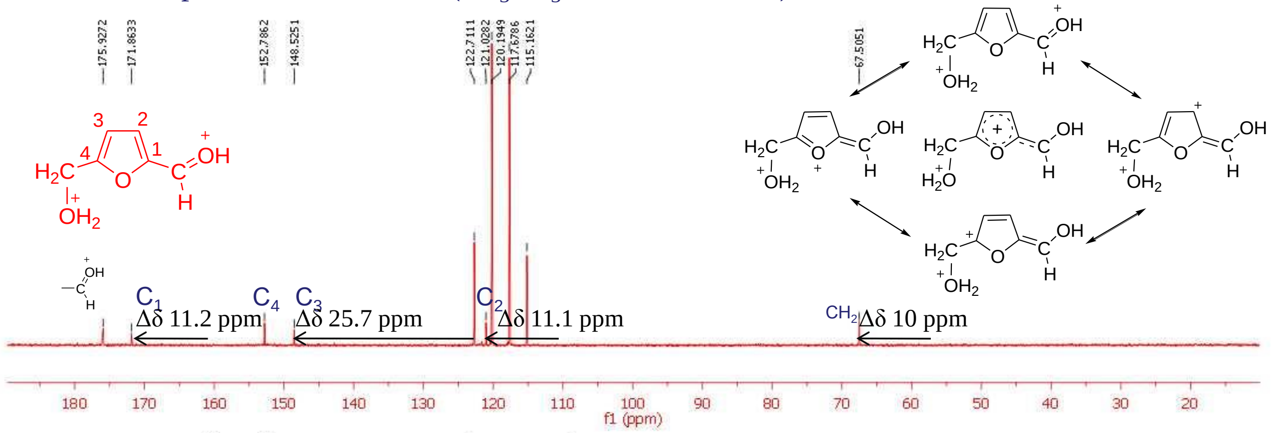

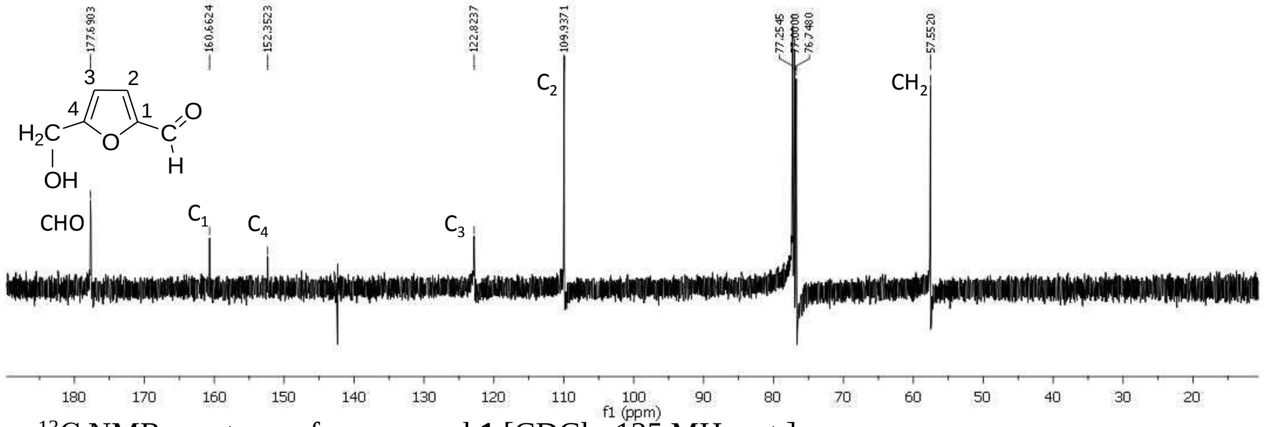

^{13}C NMR spectrum of compound **1** [$CDCl_3$, 125 MHz r.t.].

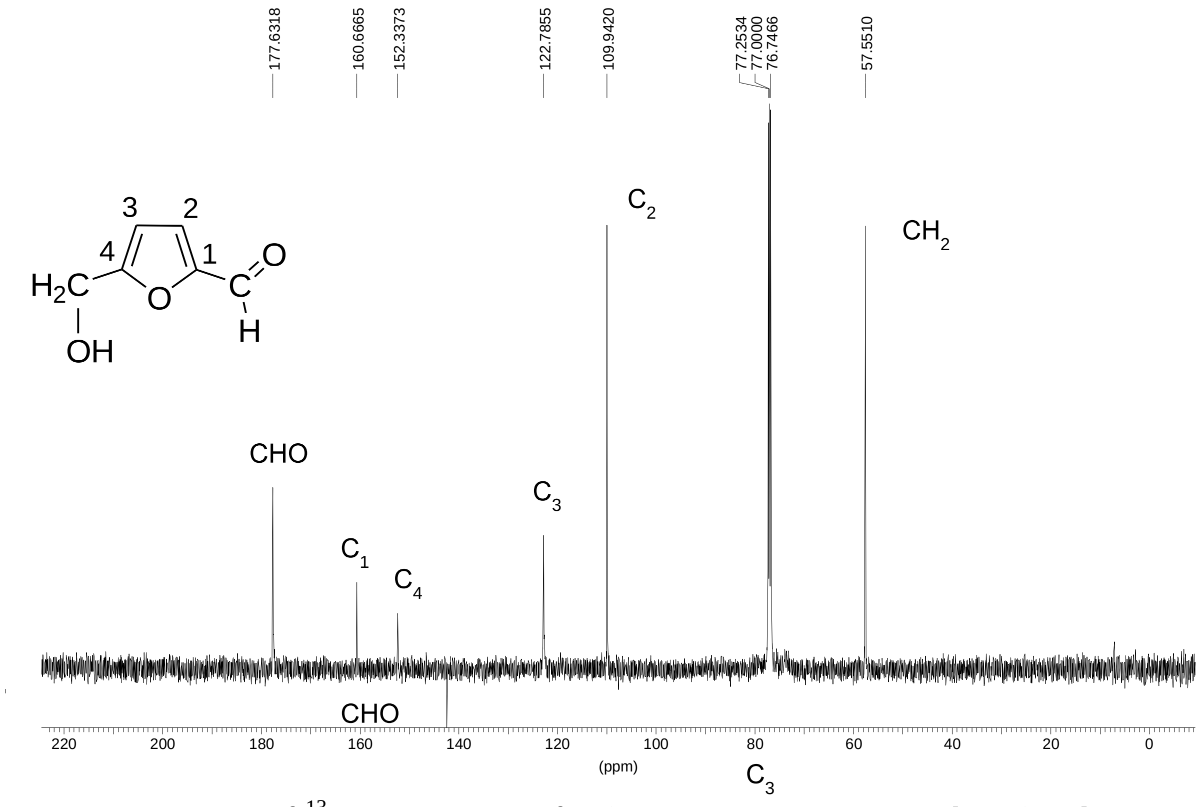

Figure S46. Comparison of ^{13}C NMR spectra of **A** (mesomeric structures are shown) and **1a** (arrows show shifts of the corresponding signals). At the bottom ^{13}C NMR spectra of **1a** ($CDCl_3$, 125 MHz).

^{1}H NMR spectrum of cation **D** [CF_3SO_3H, 500 MHz, r.t.].

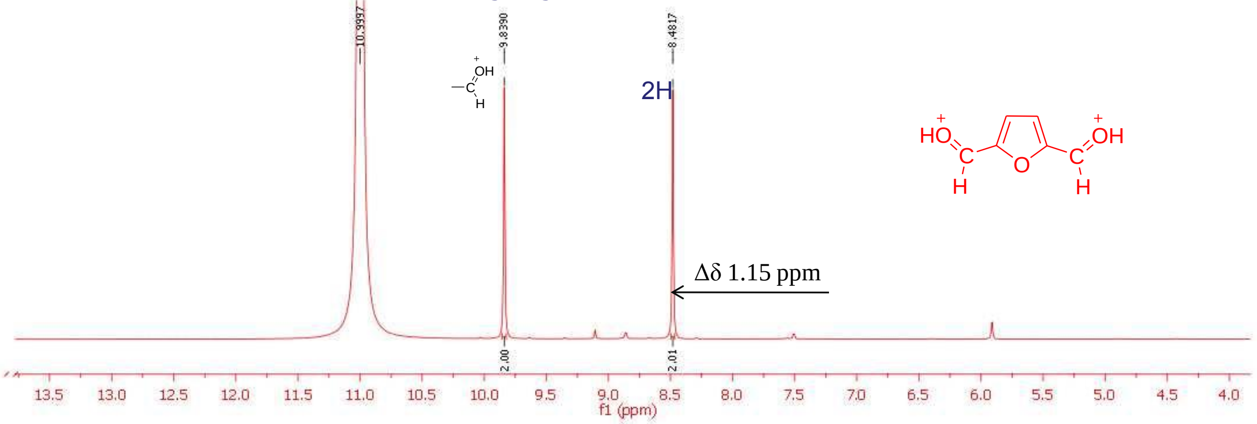

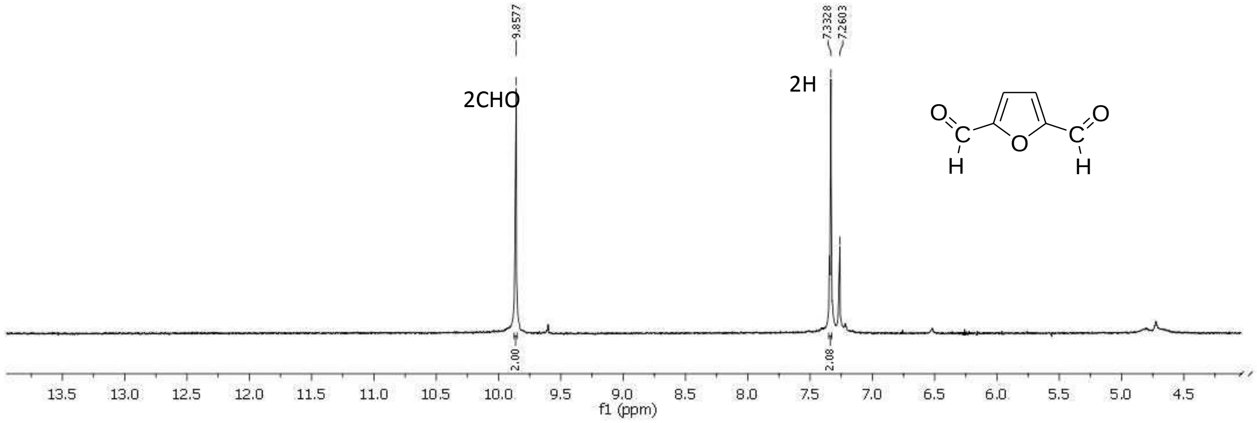

^{1}H NMR spectrum of compound **2** [$CDCl_3$, 500 MHz, r.t.].

Figure S47. Comparison of ^{1}H NMR spectra of **D** and **2** (arrows show shifts of the corresponding signals).

^{13}C NMR spectrum of cation **D** [CF_3SO_3H, 100 MHz, r.t.].

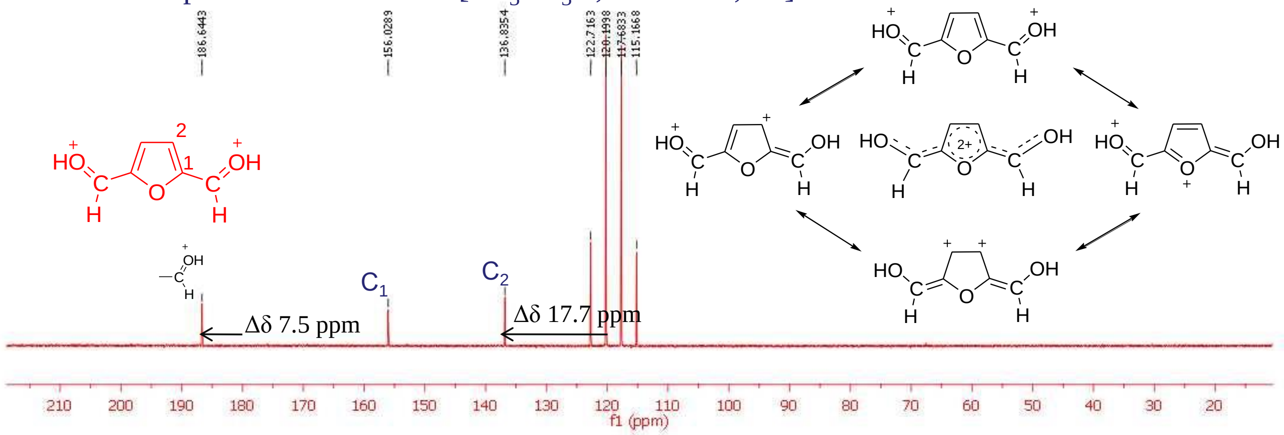

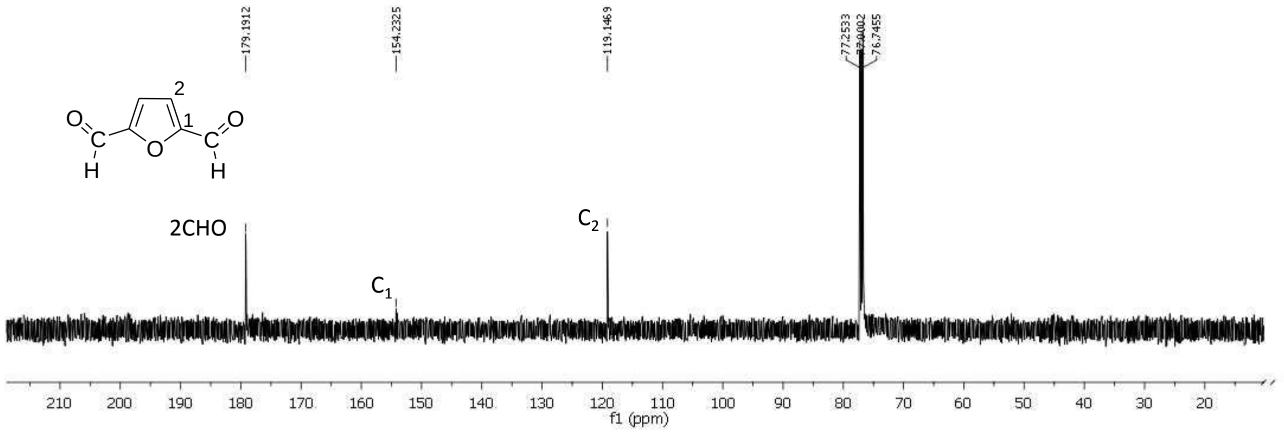

^{13}C NMR spectrum of compound **2** [$CDCl_3$, 125 MHz, r.t.].

Figure S48. Comparison of ^{13}C NMR spectra of **D** (mesomeric structures are shown) and **2** (arrows show shifts of the corresponding signals).

MALDI-MS data for oligomers obtained under the reaction of 2,5-DFF with *o*- and *m*-xylenes in TfOH

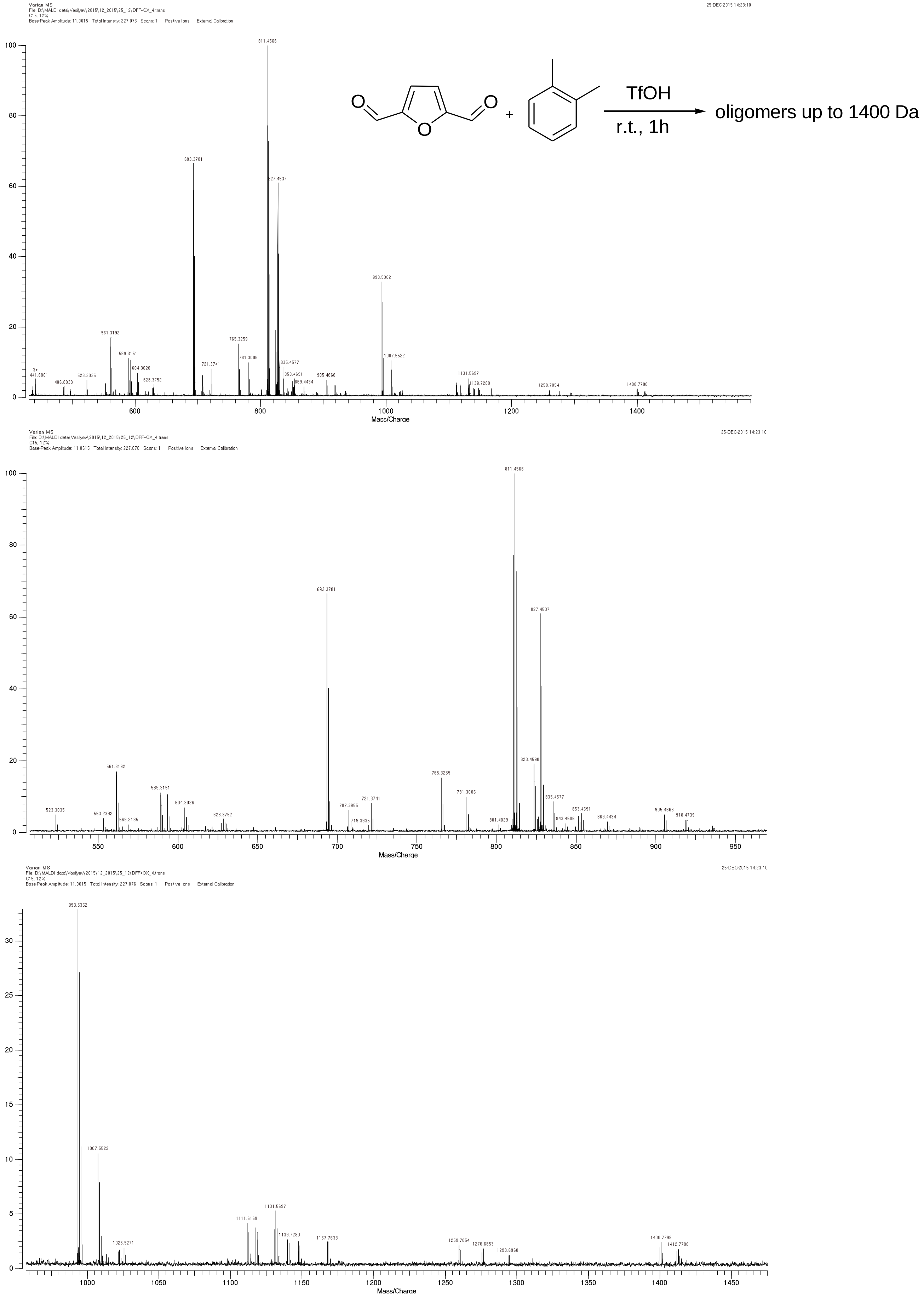

Figure S49. MALDI-MS spectrum of oligomers obtained at the reaction of **2** with *ortho*-xylene.

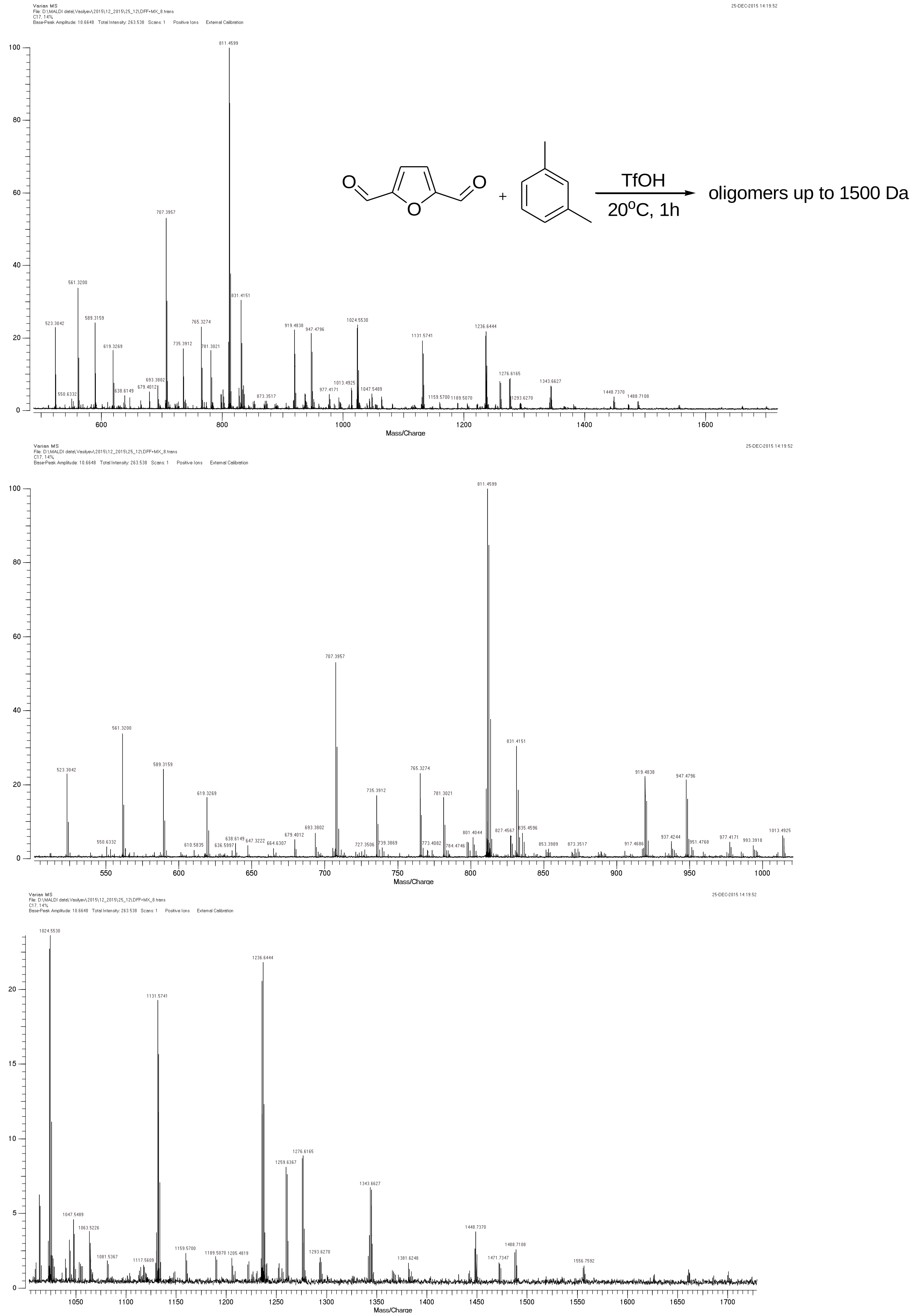

Figure S50. MALDI-MS spectrum of oligomers obtained at the reaction of **2** with *meta*-xylene.

Details of DFT-calculations of compounds 1, 2 and cations A, B, C, D

Compound 1a

Energy **E= -458.041834074 h,** **G^{298}= -457.964668 h,** **μ=6.61 D**

Cartesian coordinates, Å

N	atom	x	y	z
1	O	0.000000	0.864407	0.000000
2	C	-0.986483	-0.055234	0.000000
3	C	-0.465924	-1.320824	0.000000
4	C	0.941784	-1.170256	0.000000
5	C	1.197128	0.176033	0.000000
6	C	-2.379881	0.468972	0.000000
7	O	-3.267098	-0.641692	0.000000
8	C	2.398652	0.969177	0.000000
9	O	3.531129	0.505984	0.000000
10	H	-1.029505	-2.235689	0.000000
11	H	1.683763	-1.949320	0.000000
12	H	-2.529668	1.098175	0.883123
13	H	-2.529668	1.098175	-0.883123
14	H	-4.168596	-0.306828	0.000000
15	H	2.229774	2.058686	0.000000

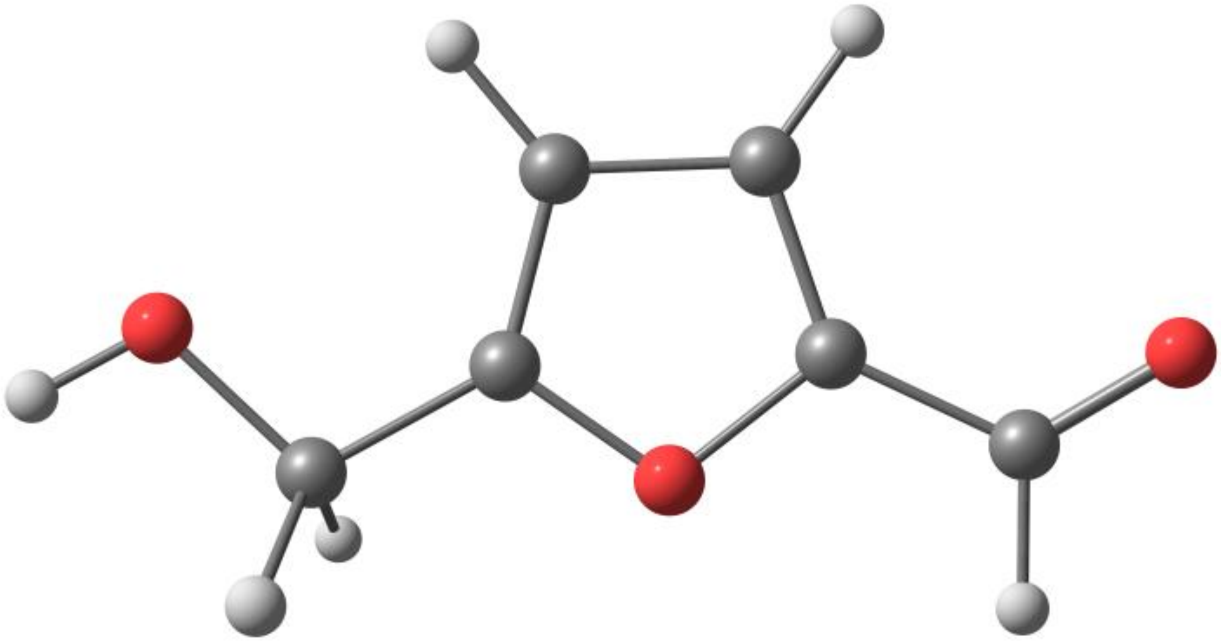

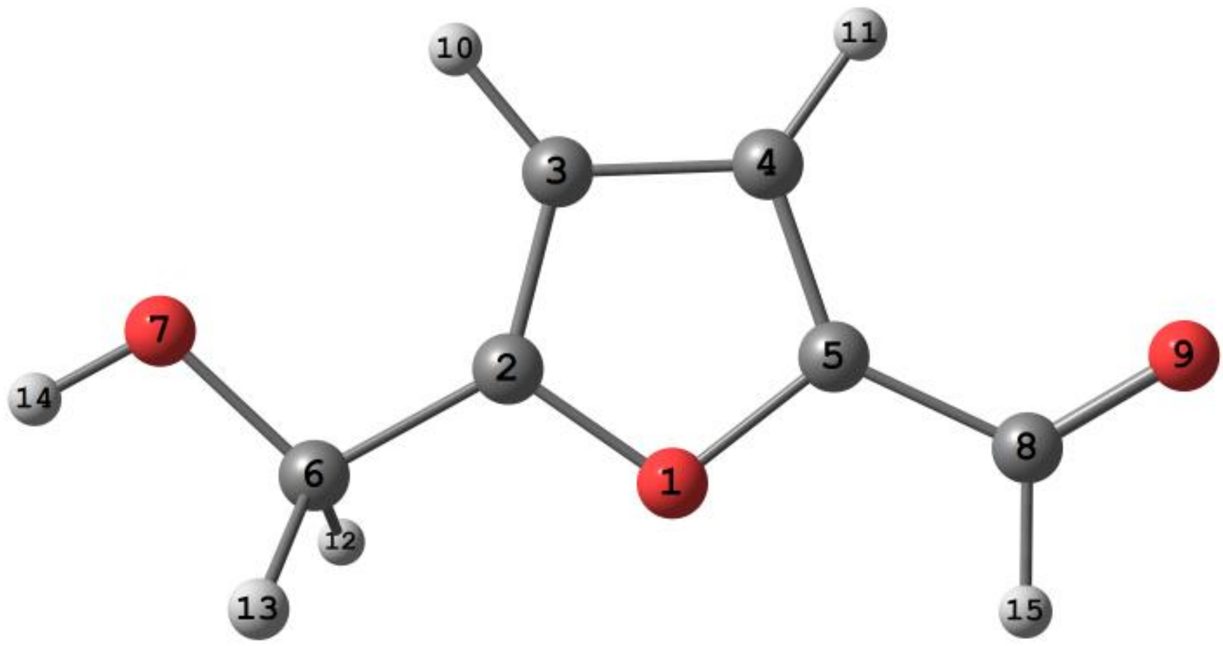

Summary of Natural Population Analysis:

Atom	No	Natural Charge	Core	Valence	Rydberg	Total
O	1	-0.46421	1.99969	6.43866	0.02586	8.46421
C	2	0.35201	1.99898	3.62440	0.02462	5.64799
C	3	-0.30165	1.99902	4.28329	0.01934	6.30165
C	4	-0.19225	1.99904	4.17480	0.01840	6.19225
C	5	0.15211	1.99891	3.82465	0.02434	5.84789
C	6	-0.06574	1.99911	4.04446	0.02218	6.06574
O	7	-0.75019	1.99978	6.73865	0.01176	8.75019
C	8	0.37870	1.99931	3.58987	0.03212	5.62130
O	9	-0.60820	1.99977	6.58343	0.02500	8.60820
H	10	0.24446	0.00000	0.75387	0.00167	0.75554
H	11	0.24186	0.00000	0.75638	0.00176	0.75814
H	12	0.19463	0.00000	0.80291	0.00246	0.80537
H	13	0.19463	0.00000	0.80291	0.00246	0.80537
H	14	0.48723	0.00000	0.50815	0.00463	0.51277
H	15	0.13661	0.00000	0.86030	0.00308	0.86339
* Total *		0.00000	17.99361	47.78673	0.21966	66.00000

(Core, Valence, Rydberg and Total are grouped under "Natural Population".)

Bond lengths, Å

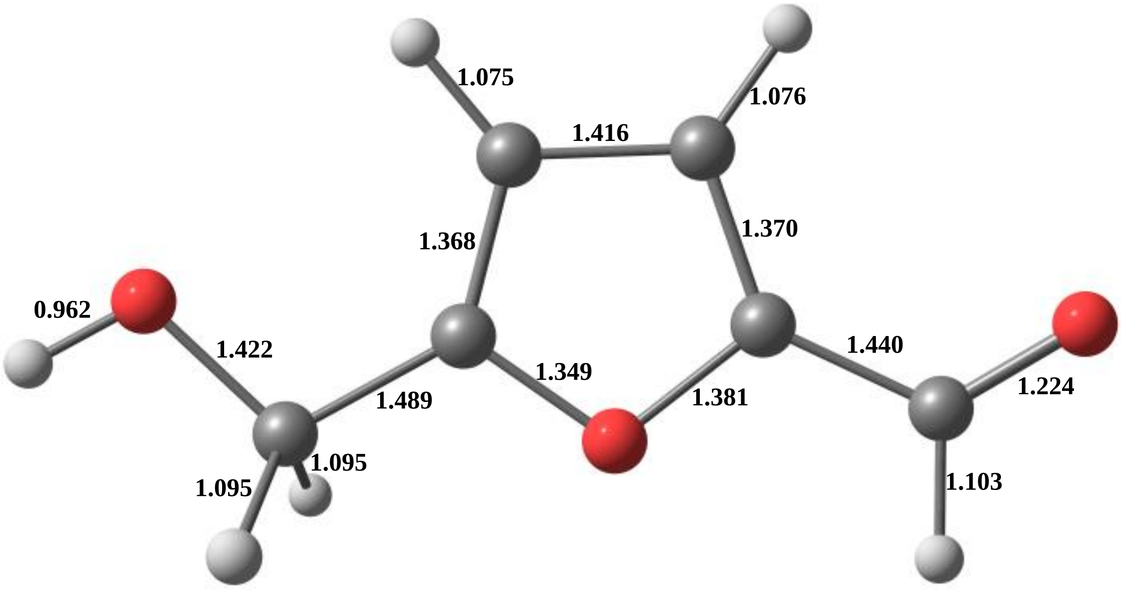
1.075
1.076
1.416
1.368
1.370
0.962
1.422
1.440
1.349
1.224
1.489
1.381
1.095
1.095
1.103

Cation A

Energy **E= -458.83480078 h,** **G^{298}= -458.72961 h,** **μ=8.11 D**

Cartesian coordinates, Å

N	atom	x	y	z
1	O	-0.034647	-0.664499	-0.329771
2	C	-0.996473	0.260455	-0.293952
3	C	-0.487065	1.509913	-0.000781
4	C	0.887770	1.340062	0.159221
5	C	1.144682	-0.013442	-0.048386
6	C	-2.374739	-0.186572	-0.588529
7	O	-3.048099	-0.572566	0.720438
8	C	2.279331	-0.812097	-0.040912
9	O	3.483550	-0.435385	0.204686
10	H	-1.050448	2.422100	0.080969
11	H	1.609190	2.104541	0.390482
12	H	-2.984046	0.624438	-0.965188
13	H	-2.412685	-1.053176	-1.237058
14	H	-4.018369	-0.469969	0.683095
15	H	2.184463	-1.868826	-0.258565
16	H	3.585496	0.510239	0.407099
17	H	-2.841074	-1.479667	1.016374

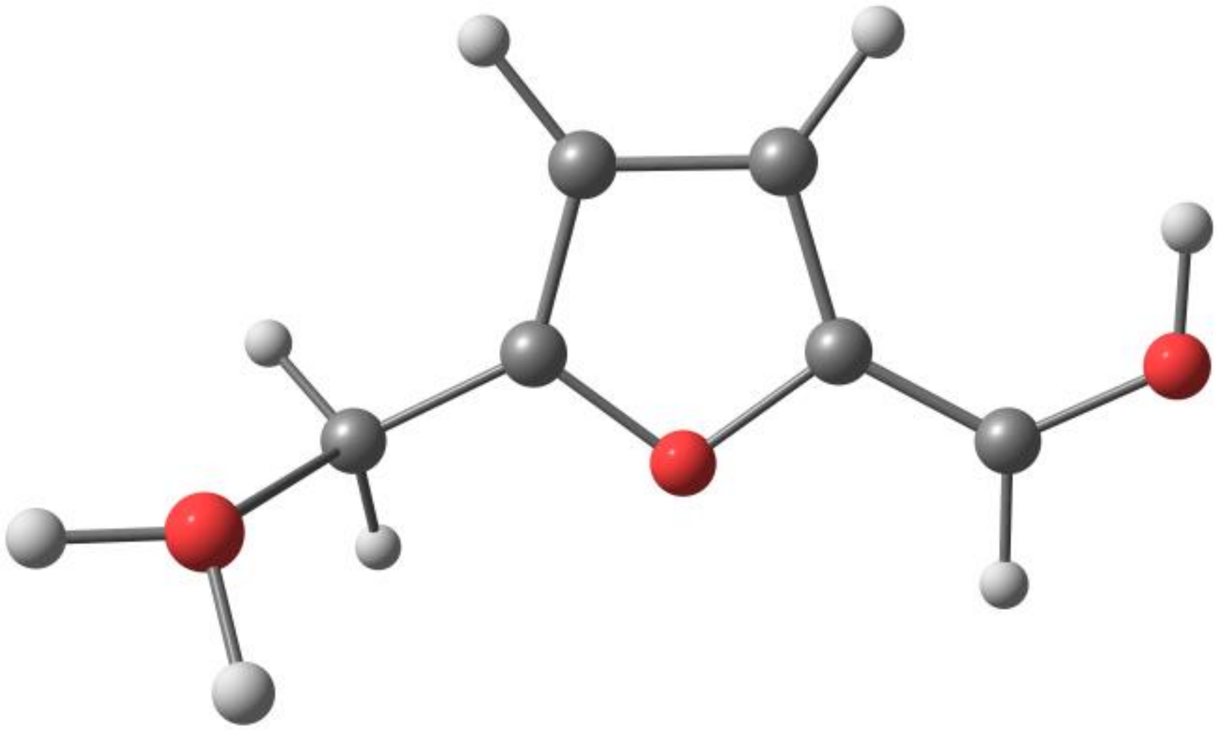

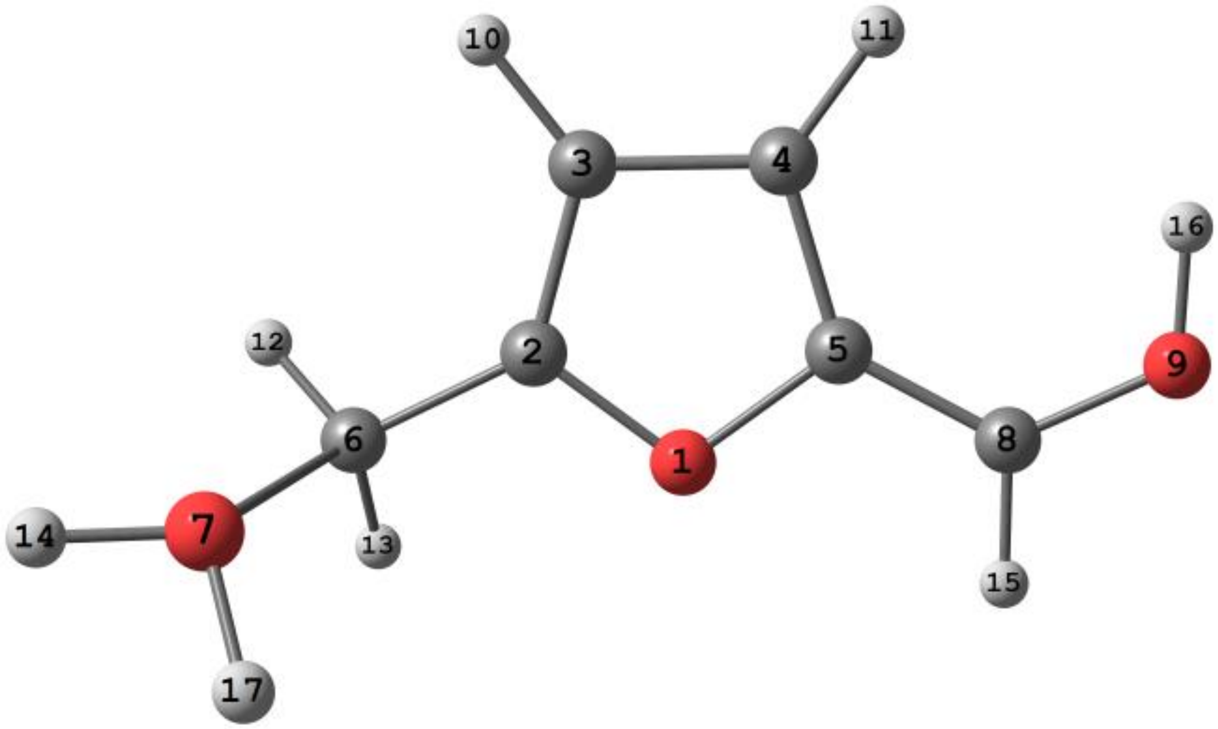

Summary of Natural Population Analysis:

Atom	No	Natural Charge	Natural Population: Core	Valence	Rydberg	Total
O	1	-0.40565	1.99968	6.38072	0.02524	8.40565
C	2	0.37106	1.99902	3.60605	0.02386	5.62894
C	3	-0.21975	1.99904	4.20222	0.01849	6.21975
C	4	-0.09663	1.99908	4.08050	0.01705	6.09663
C	5	0.15832	1.99895	3.82204	0.02069	5.84168
C	6	-0.06003	1.99899	4.04175	0.01929	6.06003
O	7	-0.62124	1.99973	6.61222	0.00929	8.62124
C	8	0.42789	1.99910	3.54737	0.02564	5.57211
O	9	-0.53880	1.99972	6.51636	0.02273	8.53880
H	10	0.27072	0.00000	0.72767	0.00161	0.72928
H	11	0.26700	0.00000	0.73150	0.00150	0.73300
H	12	0.26228	0.00000	0.73626	0.00145	0.73772
H	13	0.25756	0.00000	0.74065	0.00179	0.74244
H	14	0.57891	0.00000	0.41813	0.00295	0.42109
H	15	0.23780	0.00000	0.76064	0.00156	0.76220
H	16	0.53445	0.00000	0.46299	0.00256	0.46555
H	17	0.57608	0.00000	0.42097	0.00294	0.42392
* Total *		2.00000	17.99331	47.80805	0.19864	66.00000

Bond lengths, Å

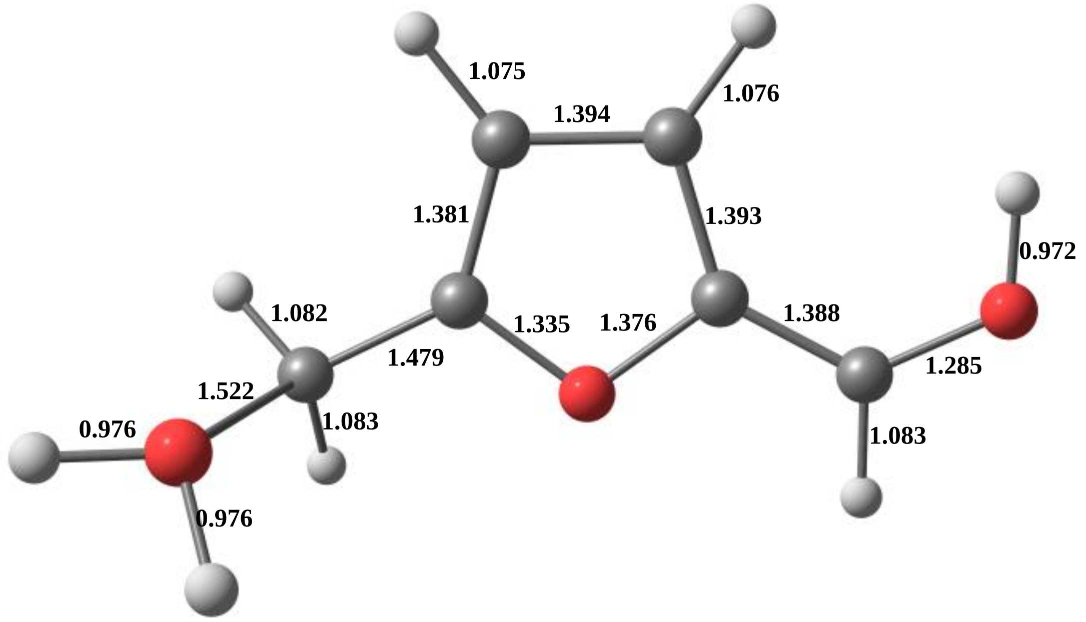
1.075
1.394
1.076
1.381
1.393
0.972
1.082
1.335
1.376
1.388
1.479
1.285
1.522
0.976
1.083
1.083
0.976

Cation B

Energy **E= -382.322457055 h, G^{298}= -382.24397 h, μ=1.69 D**

Cartesian coordinates, Å

N	atom	x	y	z
1	O	-0.443138	-0.875729	0.000657
2	C	-1.579324	-0.097073	0.000080
3	C	-1.205667	1.265717	-0.000139
4	C	0.181127	1.305297	0.000159
5	C	0.583835	-0.029310	0.000587
6	C	-2.798187	-0.697398	-0.000418
7	C	1.876294	-0.681916	-0.000158
8	O	2.992995	-0.112648	-0.000394
9	H	-1.886556	2.100022	-0.000482
10	H	0.803960	2.182990	0.000156
11	H	-3.693034	-0.091669	-0.001234
12	H	-2.885630	-1.775051	-0.000139
13	H	1.909434	-1.767012	-0.001161
14	H	3.004495	0.865838	0.000089

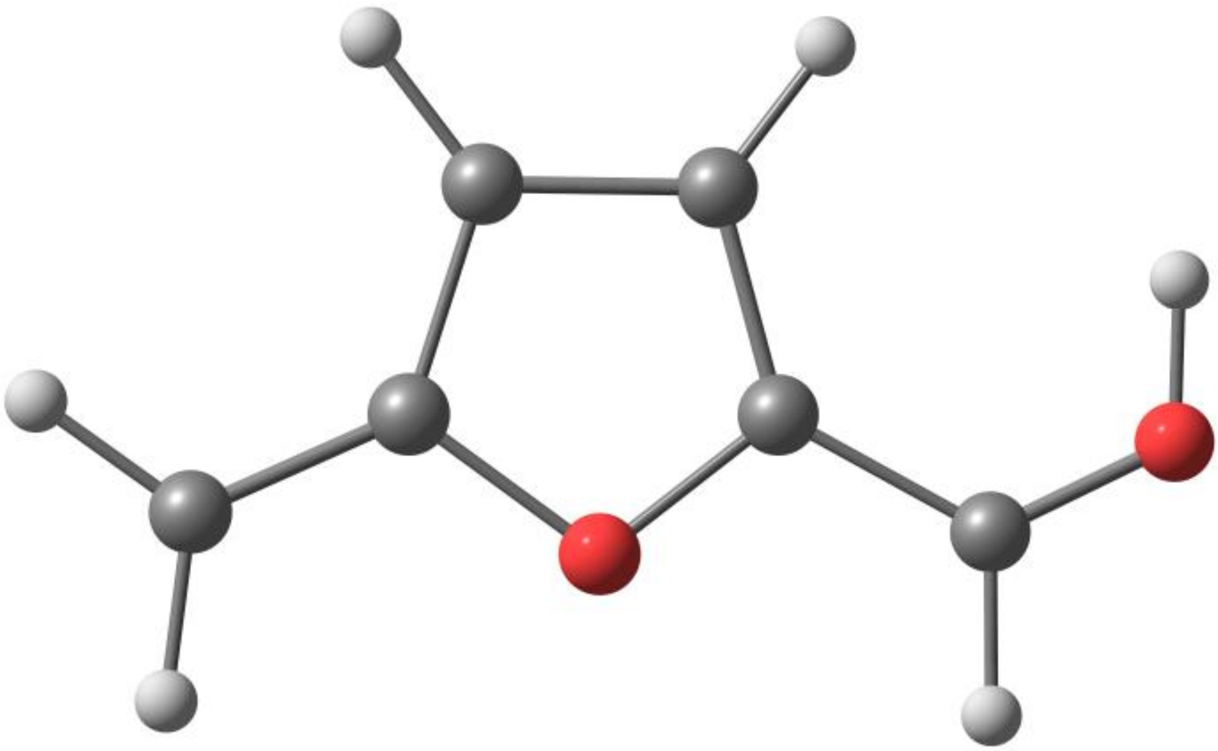

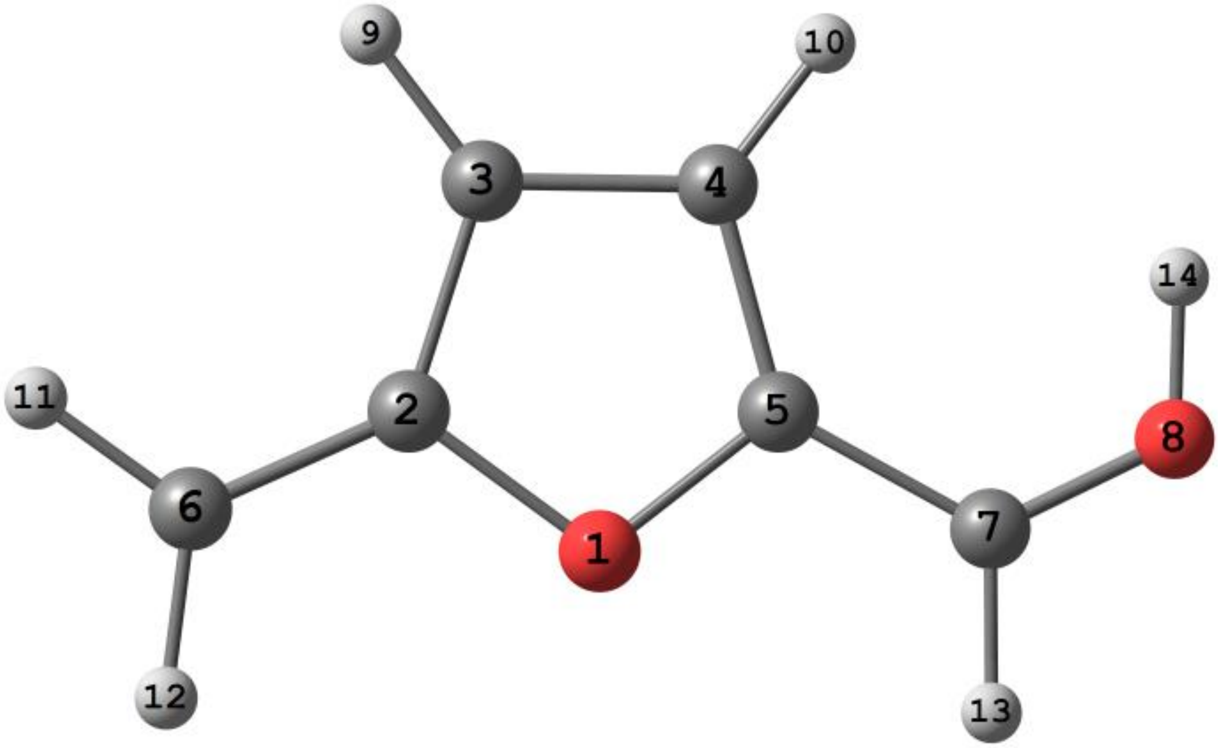

Summary of Natural Population Analysis:

Atom	No	Natural Charge	Natural Population Core	Valence	Rydberg	Total
O	1	-0.37376	1.99968	6.34862	0.02545	8.37376
C	2	0.20914	1.99901	3.77025	0.02160	5.79086
C	3	-0.01031	1.99909	3.99406	0.01716	6.01031
C	4	-0.16214	1.99907	4.14528	0.01779	6.16214
C	5	0.31326	1.99904	3.66681	0.02089	5.68674
C	6	0.05403	1.99915	3.93465	0.01217	5.94597
C	7	0.52780	1.99927	3.44718	0.02575	5.47220
O	8	-0.45322	1.99969	6.42998	0.02356	8.45322
H	9	0.28457	0.00000	0.71402	0.00142	0.71543
H	10	0.28751	0.00000	0.71080	0.00169	0.71249
H	11	0.25309	0.00000	0.74581	0.00110	0.74691
H	12	0.25219	0.00000	0.74645	0.00136	0.74781
H	13	0.25731	0.00000	0.74120	0.00150	0.74269
H	14	0.56054	0.00000	0.43707	0.00239	0.43946
* Total *		2.00000	15.99401	39.83217	0.17382	56.00000

Bond lengths, Å

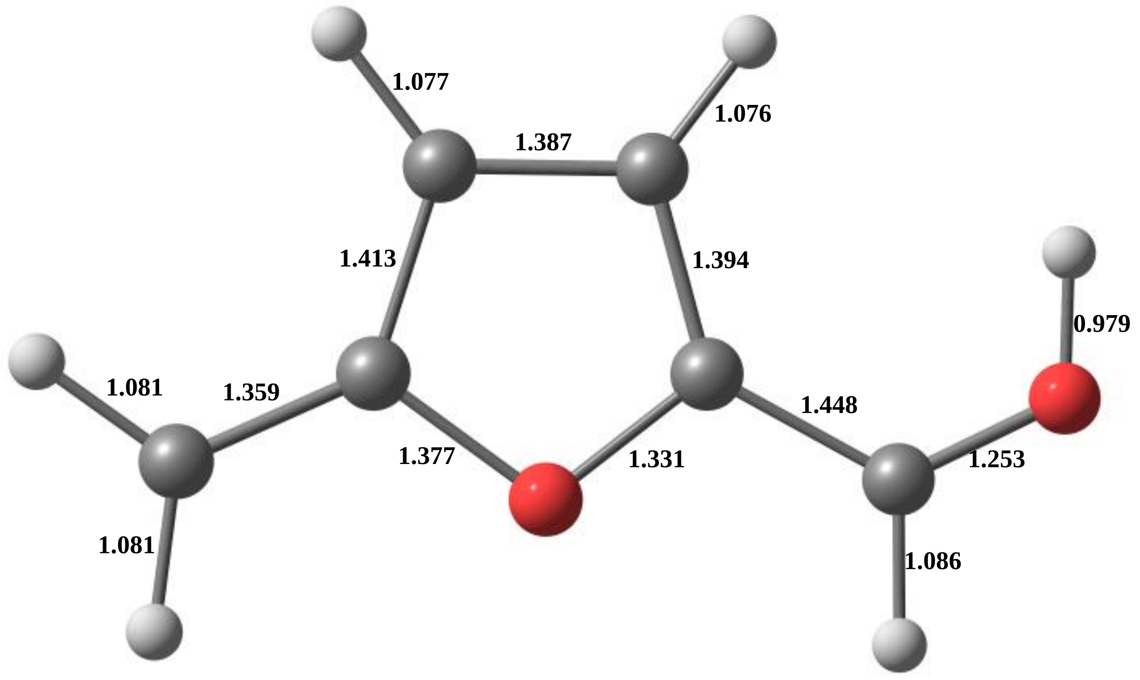
1.077
1.387
1.076
1.413
1.394
0.979
1.081
1.359
1.448
1.377
1.331
1.253
1.081
1.086

Compound 2

Energy **E= -456.829375906 h, G^{298}= -456.773016 h, μ=0.44 D**

Cartesian coordinates, Å

N	atom	x	y	z
1	C	0.000000	-1.092442	0.024938
2	C	0.000000	-0.704168	-1.293969
3	C	0.000000	0.704168	-1.293969
4	C	0.000000	1.092442	0.024938
5	C	0.000000	0.000000	0.835837
6	C	0.000000	2.392438	0.678584
7	O	0.000000	3.446190	0.070458
8	C	0.000000	-2.392438	0.678584
9	O	0.000000	-3.446190	0.070458
10	H	0.000000	-1.363436	-2.143574
11	H	0.000000	1.363436	-2.143574
12	H	0.000000	2.358377	1.779244
13	H	0.000000	-2.358377	1.779244

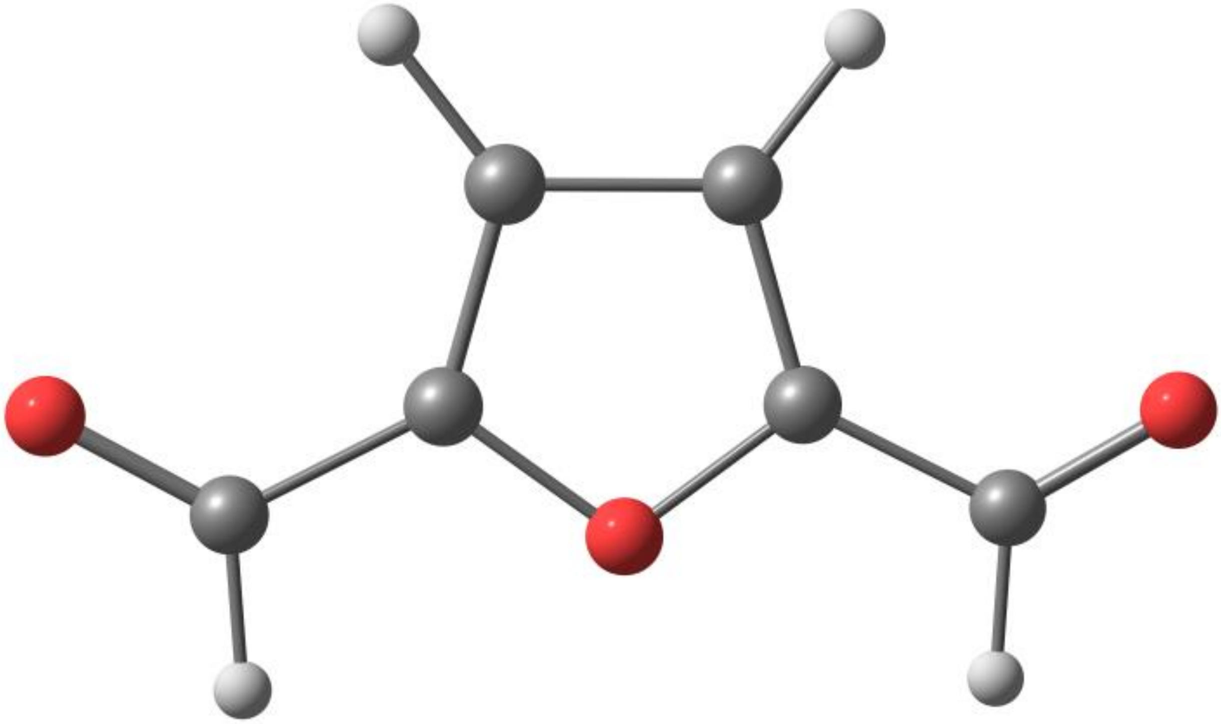

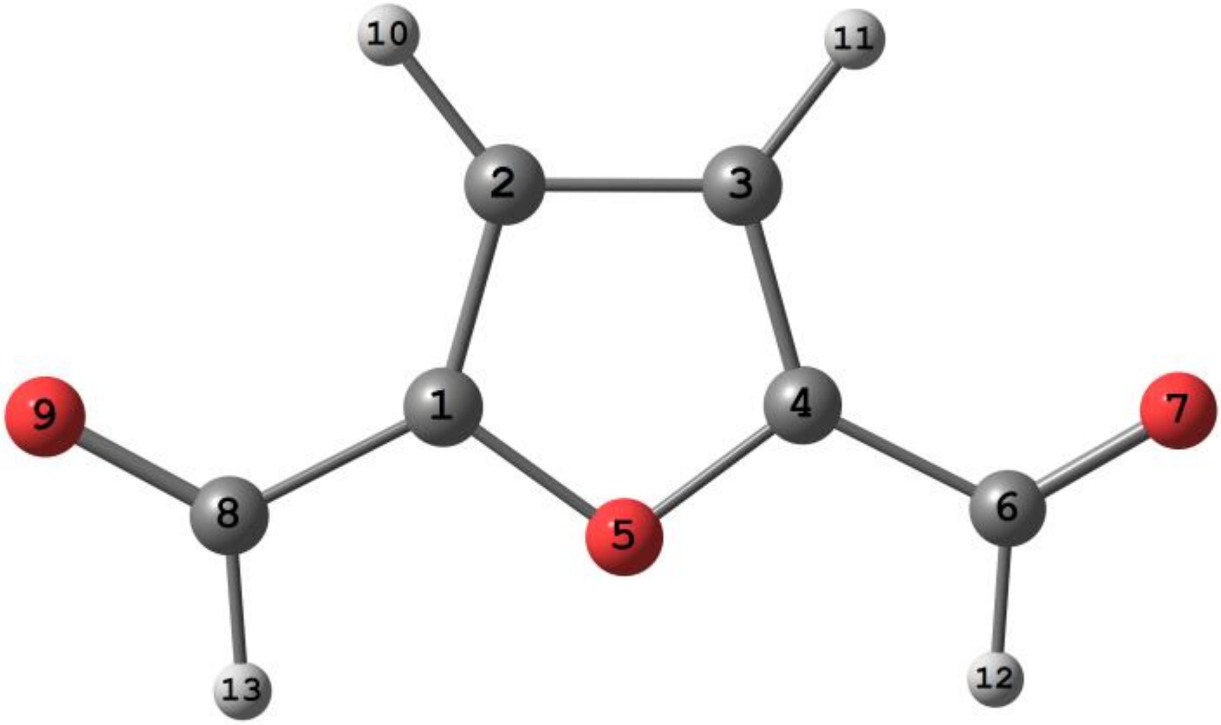

Summary of Natural Population Analysis:

Atom	No	Natural Charge	Core	Valence	Rydberg	Total
C	1	0.20968	1.99895	3.76720	0.02416	5.79032
C	2	-0.20918	1.99905	4.19188	0.01825	6.20918
C	3	-0.20918	1.99905	4.19188	0.01825	6.20918
C	4	0.20968	1.99895	3.76720	0.02416	5.79032
O	5	-0.43639	1.99970	6.41236	0.02433	8.43639
C	6	0.39144	1.99935	3.57741	0.03181	5.60856
O	7	-0.56826	1.99977	6.54363	0.02485	8.56826
C	8	0.39144	1.99935	3.57741	0.03181	5.60856
O	9	-0.56826	1.99977	6.54363	0.02485	8.56826
H	10	0.24957	0.00000	0.74869	0.00174	0.75043
H	11	0.24957	0.00000	0.74869	0.00174	0.75043
H	12	0.14493	0.00000	0.85216	0.00290	0.85507
H	13	0.14493	0.00000	0.85216	0.00290	0.85507
* Total *		0.00000	17.99394	45.77430	0.23176	64.00000

(Core, Valence, Rydberg and Total are grouped under "Natural Population".)

Bond lengths, Å

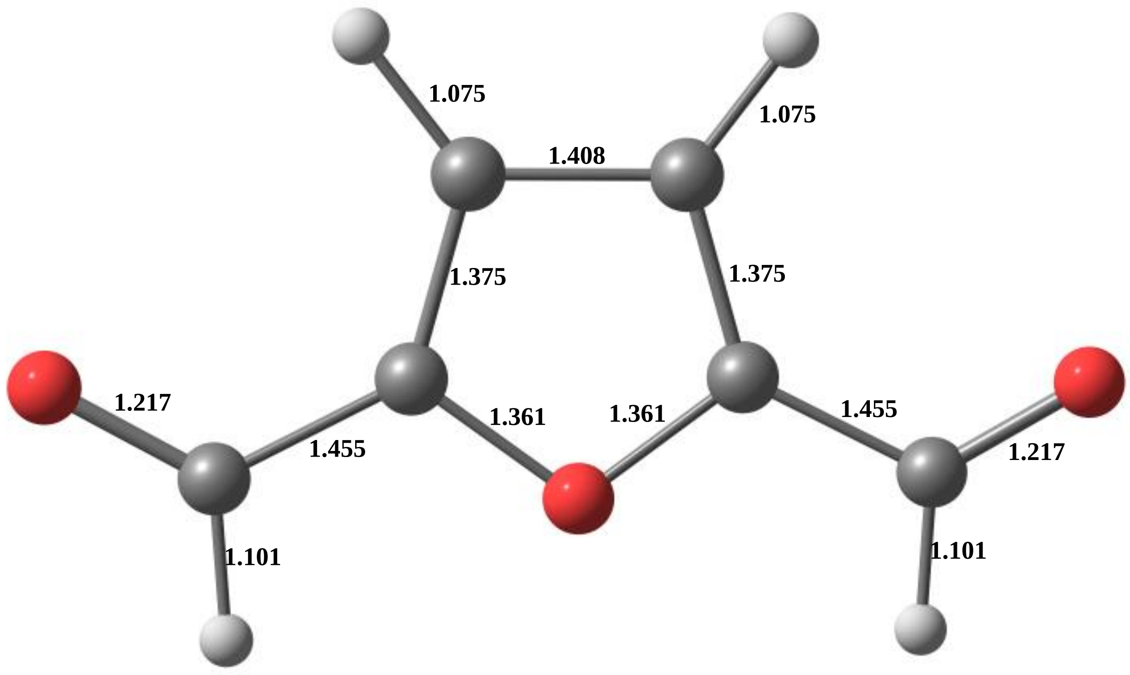
1.075
1.075
1.408
1.375
1.375
1.217
1.361
1.361
1.455
1.455
1.217
1.101
1.101

Cation C

Energy **E= -457.230436542 h, G^{298}= -457.161104 h, μ=8.27 D**

Cartesian coordinates, Å

N	atom	x	y	z
1	C	-1.120282	0.128944	0.000000
2	C	-0.847112	-1.229886	0.000000
3	C	0.538545	-1.344397	0.000000
4	C	1.045949	-0.042077	0.000000
5	O	0.000000	0.852180	0.000000
6	C	2.311716	0.521564	0.000000
7	O	3.437887	-0.103273	0.000000
8	C	-2.399780	0.871803	0.000000
9	O	-3.460135	0.294496	0.000000
10	H	-1.574695	-2.020974	0.000000
11	H	1.110430	-2.256356	0.000000
12	H	2.409071	1.600222	0.000000
13	H	-2.311546	1.966977	0.000000
14	H	3.370513	-1.072801	0.000000

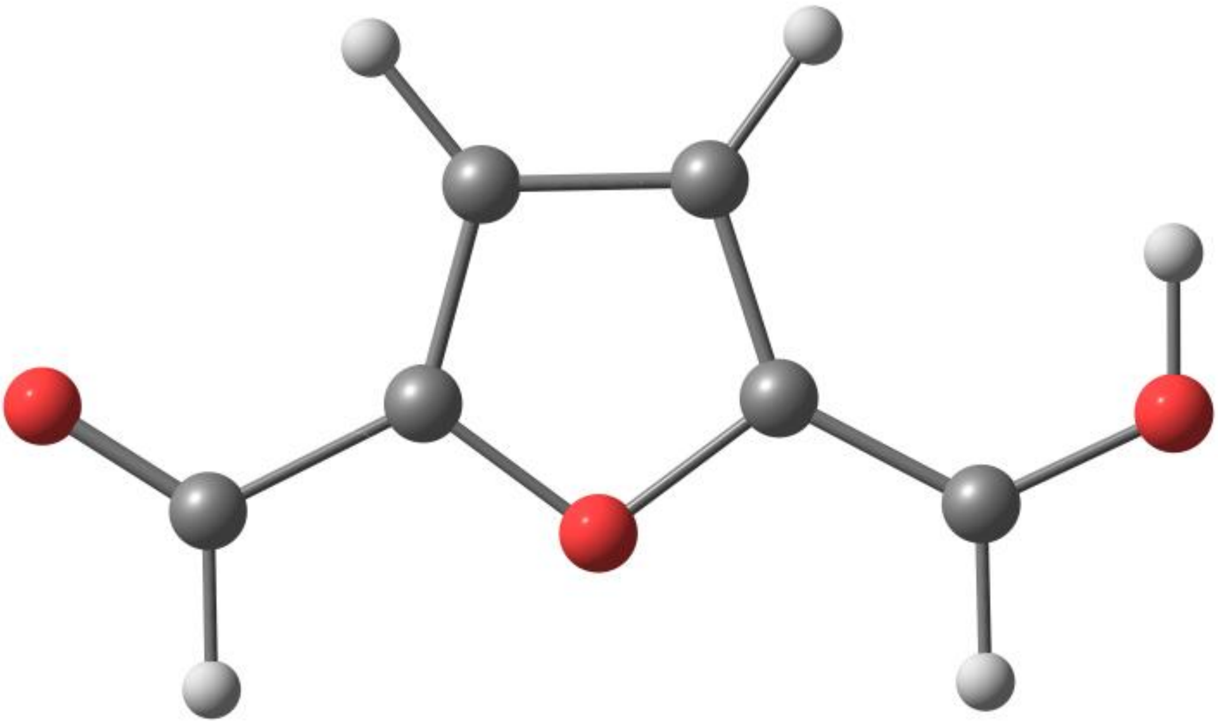

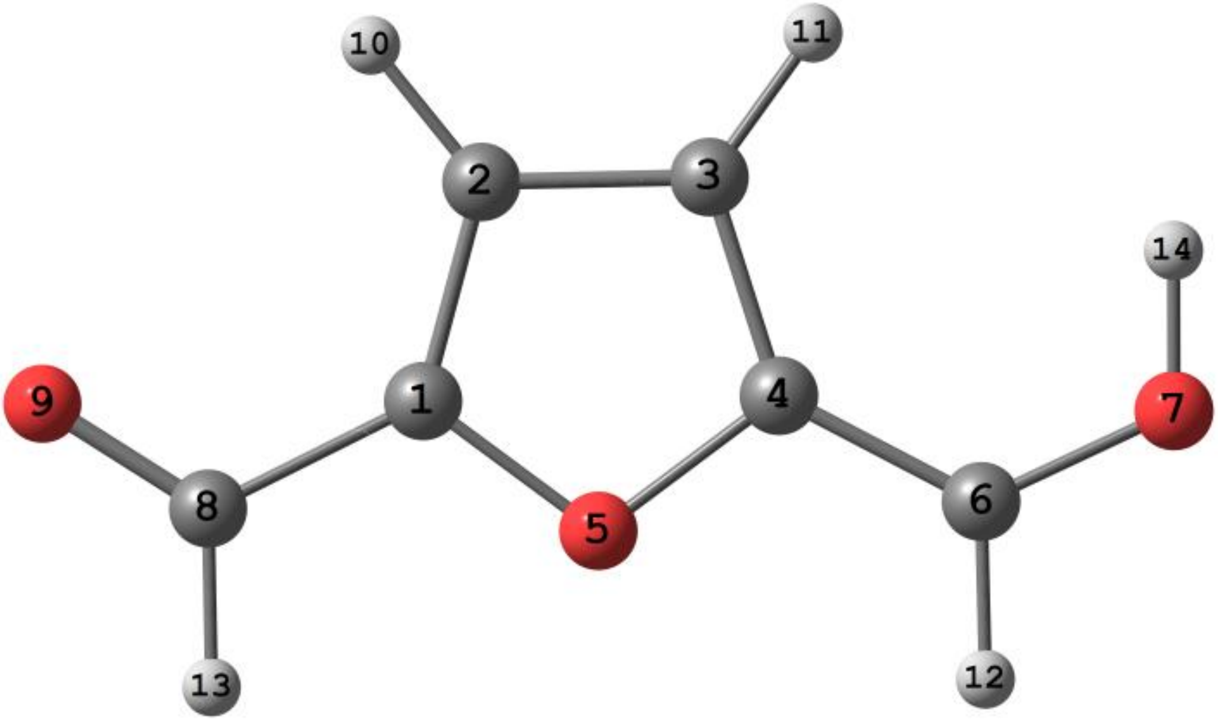

Summary of Natural Population Analysis:

Atom	No	Natural Charge	Core	Valence	Rydberg	Total
C	1	0.32980	1.99902	3.64685	0.02433	5.67020
C	2	-0.21737	1.99906	4.19928	0.01902	6.21737
C	3	-0.10552	1.99908	4.08938	0.01706	6.10552
C	4	0.16148	1.99896	3.81875	0.02081	5.83852
O	5	-0.40416	1.99969	6.37912	0.02535	8.40416
C	6	0.41692	1.99909	3.55823	0.02576	5.58308
O	7	-0.54471	1.99972	6.52237	0.02262	8.54471
C	8	0.40043	1.99939	3.56892	0.03127	5.59957
O	9	-0.50246	1.99977	6.47805	0.02464	8.50246
H	10	0.26824	0.00000	0.72988	0.00187	0.73176
H	11	0.26448	0.00000	0.73401	0.00151	0.73552
H	12	0.23609	0.00000	0.76234	0.00157	0.76391
H	13	0.16363	0.00000	0.83426	0.00211	0.83637
H	14	0.53315	0.00000	0.46427	0.00258	0.46685
* Total *		1.00000	17.99377	45.78571	0.22052	64.00000

Bond lengths, Å

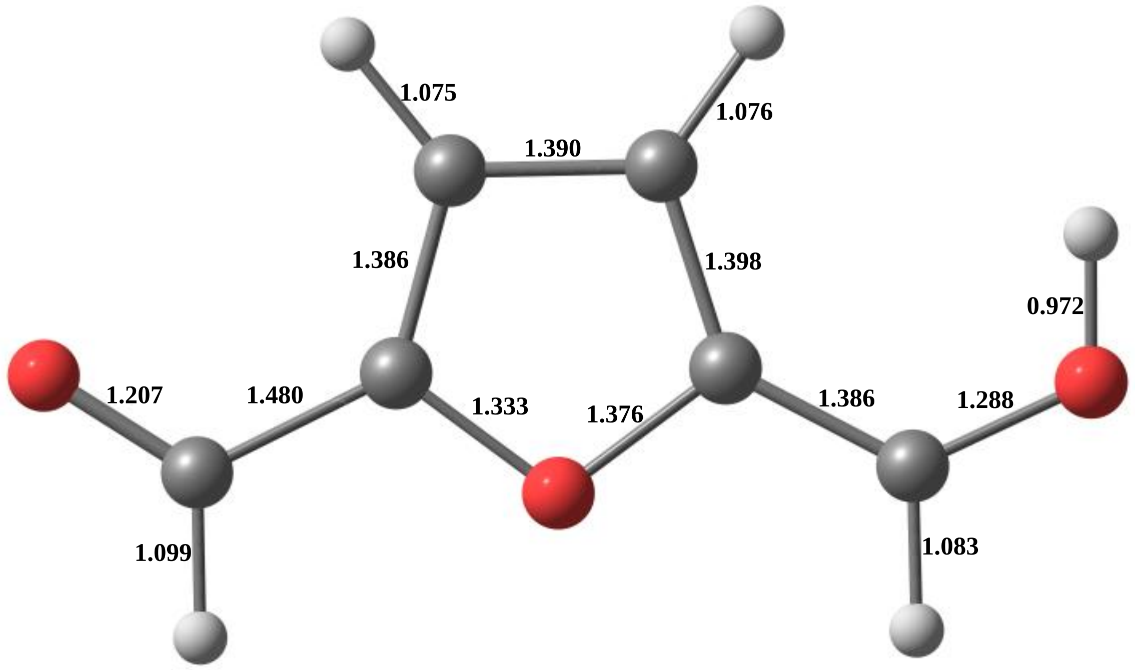
1.075
1.076
1.390
1.386
1.398
0.972
1.207
1.480
1.333
1.376
1.386
1.288
1.099
1.083

Cation D

Energy **E= -457.60669012 h, G^{298}= -457.523348 h, μ=2.80 D**

Cartesian coordinates, Å

N	atom	x	y	z
1	C	-0.015980	-1.081155	0.000000
2	C	1.319768	-0.697717	0.000000
3	C	1.319768	0.697717	0.000000
4	C	-0.015980	1.081155	0.000000
5	O	-0.828188	0.000000	0.000000
6	C	-0.703129	2.321798	0.000000
7	O	-0.184974	3.477155	0.000000
8	C	-0.703128	-2.321798	0.000000
9	O	-0.184974	-3.477155	0.000000
10	H	2.186249	-1.335940	0.000000
11	H	2.186249	1.335941	0.000000
12	H	-1.787533	2.313127	0.000000
13	H	-1.787533	-2.313128	0.000000
14	H	0.789872	3.520973	0.000000
15	H	0.789873	-3.520972	0.000000

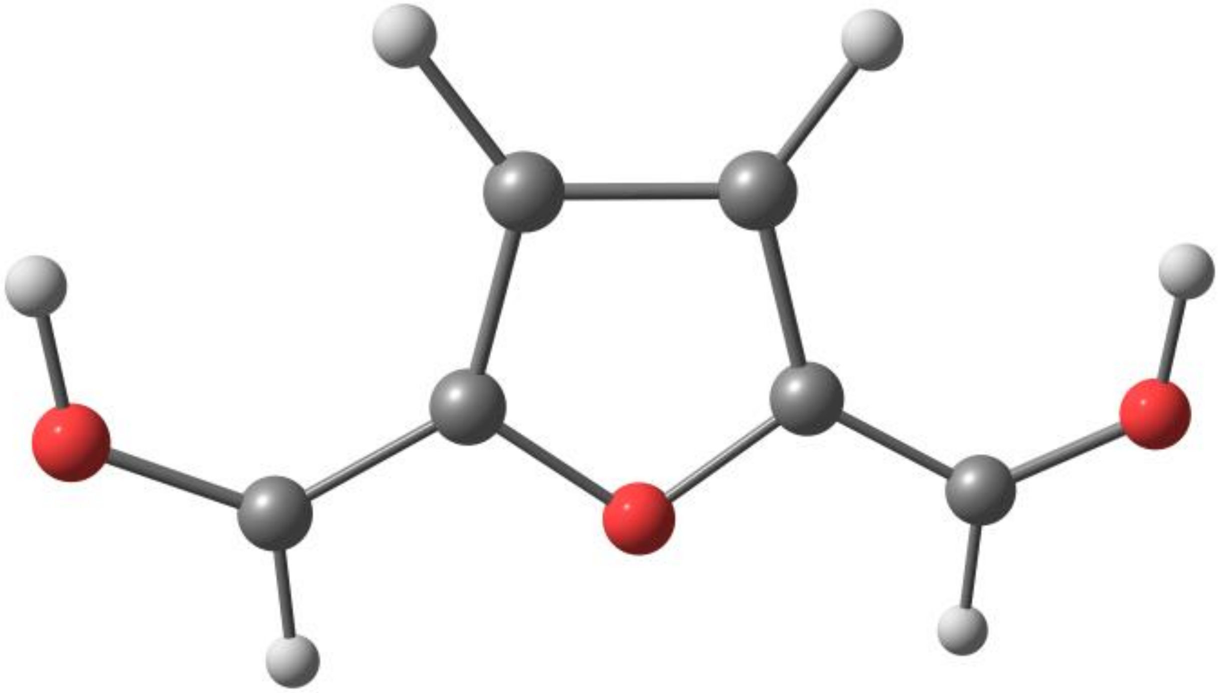

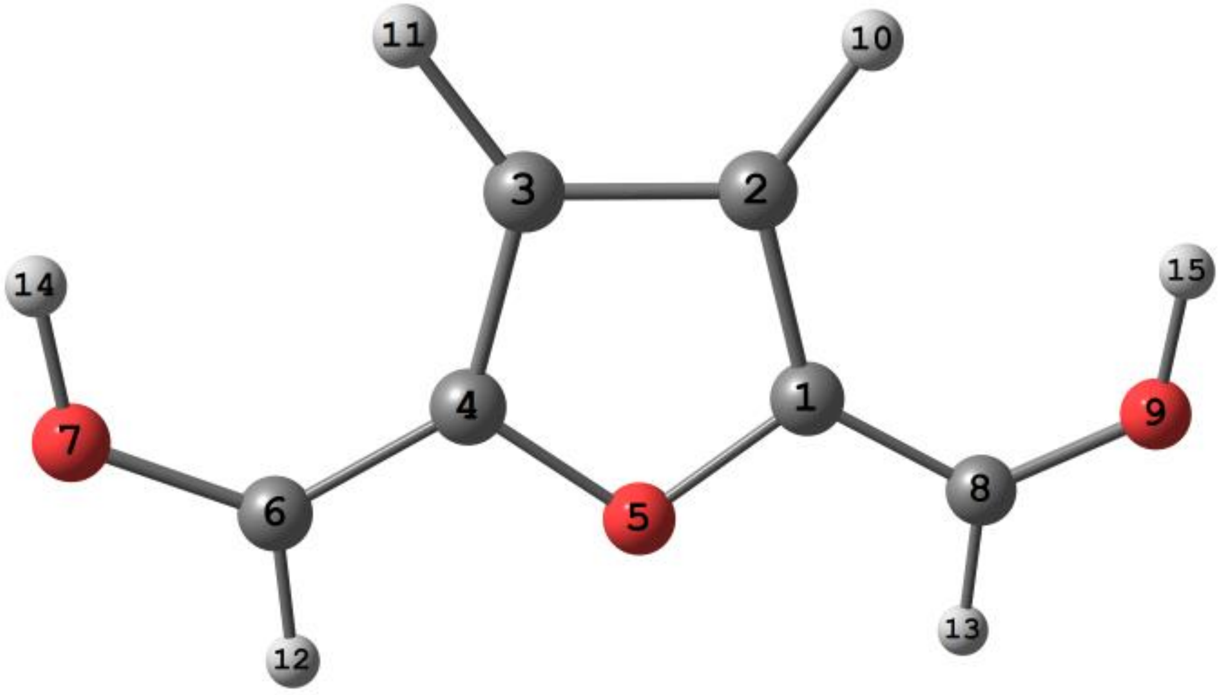

Summary of Natural Population Analysis:

Atom	No	Natural Charge	Natural Population Core	Valence	Rydberg	Total
C	1	0.22845	1.99901	3.75205	0.02050	5.77155
C	2	-0.11924	1.99908	4.10252	0.01764	6.11924
C	3	-0.11924	1.99908	4.10252	0.01764	6.11924
C	4	0.22845	1.99901	3.75205	0.02050	5.77155
O	5	-0.37561	1.99969	6.35168	0.02425	8.37561
C	6	0.48830	1.99920	3.48679	0.02571	5.51170
O	7	-0.48915	1.99970	6.46631	0.02313	8.48915
C	8	0.48830	1.99920	3.48679	0.02571	5.51170
O	9	-0.48915	1.99970	6.46631	0.02313	8.48915
H	10	0.28056	0.00000	0.71777	0.00167	0.71944
H	11	0.28056	0.00000	0.71777	0.00167	0.71944
H	12	0.24898	0.00000	0.74950	0.00152	0.75102
H	13	0.24898	0.00000	0.74950	0.00152	0.75102
H	14	0.54990	0.00000	0.44764	0.00246	0.45010
H	15	0.54990	0.00000	0.44764	0.00246	0.45010
* Total *		2.00000	17.99368	45.79682	0.20950	64.00000

Bond lengths, Å

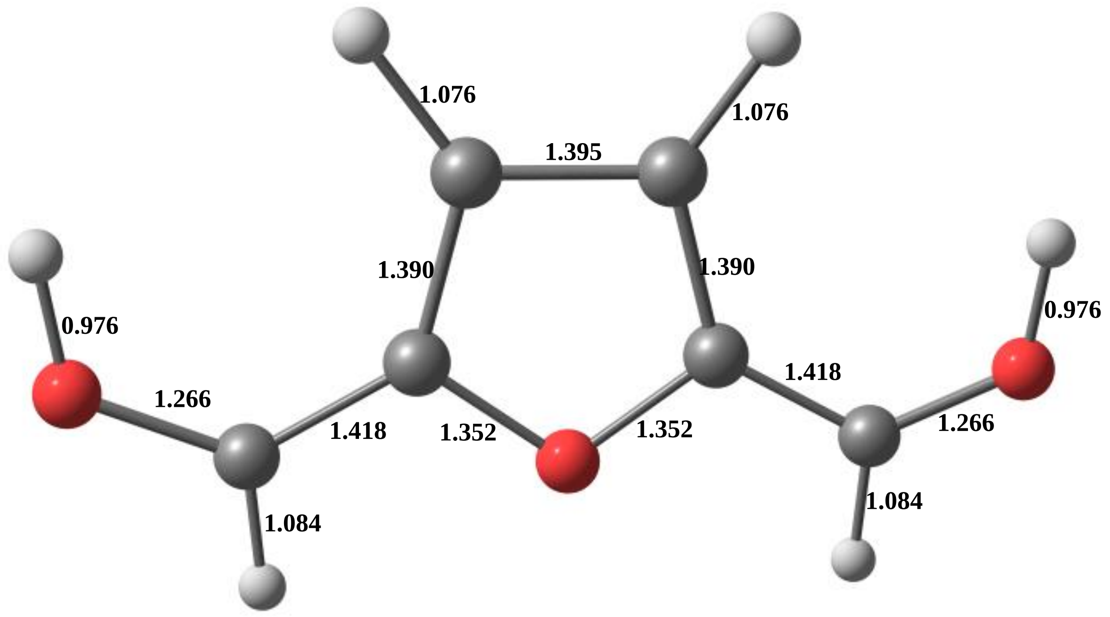
1.076
1.076
1.395
1.390
1.390
0.976
0.976
1.266
1.418
1.418
1.352
1.352
1.266
1.084
1.084
